# Assessing Weak Anion Binding to Small Peptides

**DOI:** 10.1021/acs.jpcb.4c00657

**Published:** 2024-04-09

**Authors:** Corinne L. D. Gibb, Thien H. Tran, Bruce C. Gibb

**Affiliations:** Department of Chemistry, Tulane University School of Science and Engineering, New Orleans, Louisiana 70118, United States

## Abstract

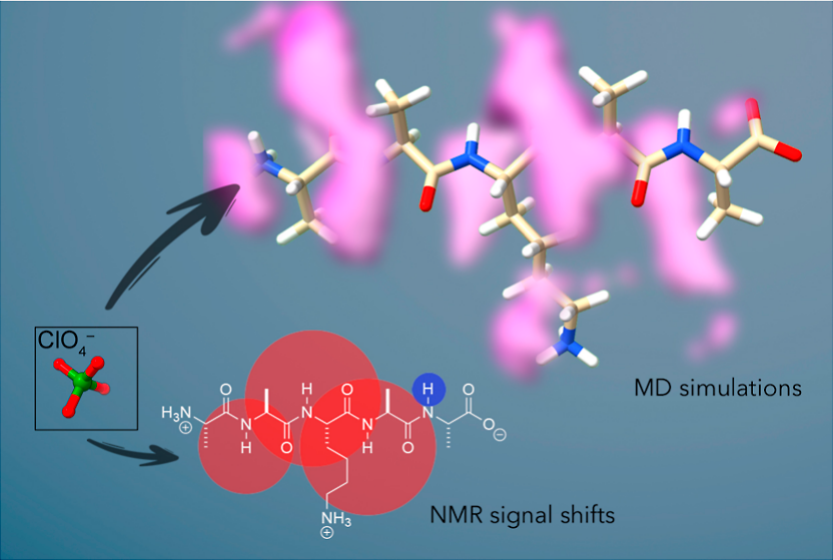

Since Hofmeister’s seminal studies in the late 19th century, it has been known that salts and buffers can drastically affect the properties of peptides and proteins. These Hofmeister effects can be conceived of in terms of three distinct phenomena/mechanisms: water–salt interactions that indirectly induce the salting-out of a protein by water sequestration by the salt, and direct salt–protein interactions that can either salt-in or salt-out the protein. Unfortunately, direct salt–protein interactions responsible for Hofmeister effects are weak and difficult to quantify. As such, they are frequently construed of as being nonspecific. Nevertheless, there has been considerable effort to better specify these interactions. Here, we use pentapeptides to demonstrate the utility of the H-dimension of nuclear magnetic resonance (NMR) spectroscopy to assess anion binding using N–H signal shifts. We qualify binding using these, demonstrating the upfield shifts induced by anion association and revealing how they are much larger than the corresponding downfield shifts induced by magnetic susceptibility and other ionic strength change effects. We also qualify binding in terms of how the pattern of signal shifts changes with point mutations. In general, we find that the observed upfield shifts are small compared with those induced by anion binding to amide-based hosts, and MD simulations suggest that this is so. Thus, charge-diffuse anions associate mostly with the nonpolar regions of the peptide rather than directly interacting with the amide N–H groups. These findings reveal the utility of ^1^H NMR spectroscopy for qualifying affinity to peptides—even when affinity constants are very low—and serve as a benchmark for using NMR spectroscopy to study anion binding to more complex systems.

## Introduction

Salts and buffers can drastically affect the properties of peptides and proteins. Such Hofmeister effects have been recognized since the 1880/90s^1−4^ and can be conceived of in terms of three distinct phenomena/mechanisms: water–salt interactions that indirectly induce the salting-out of a protein by water sequestration by the salt, and direct salt–protein interactions that can either salt-in or salt-out the protein. Whether direct salt–protein interactions increase or decrease the solubility of a protein is controlled by a complex relationship between the (salt) cation and anion affinities for the peptide or protein and depends on whether ion binding increases the net charge of the protein or reduces it toward net zero. For example, anion binding to a positively charged protein, i.e., one at a pH below its pI, induces its precipitation by charge attenuation. This phenomenon is known as the reverse or inverse Hofmeister effect and is perhaps most graphically demonstrated by highly basic lysozyme.^5−10^ The balance between the reverse Hofmeister effect and direct ion–protein interactions that salt-in the protein is not only controlled by pH and the very nature of the protein but also made more complex by structural changes to the protein induced by ion-binding.

For all facets of the Hofmeister conundrum, there are open questions: about the precise supramolecular interactions between the cations and anions of a salt and the functionality of a protein; about the relative affinities of these; and how ion-binding changes the protein structure.

Arguably the most obvious noncovalent interactions (NCIs) are Coulombic interactions between charges. However, even this conceptually straightforward NCI is made more complex by the asymmetric hydration of charges (M^+^···OH_2_ versus X^–^···H–OH), the nonpoint-charge nature of many of the ions/charged groups involved,^11^ and the possibility of ion-pairing.^12−15^ The first two of these factors mean, for example, that the anionic groups in proteins are more strongly solvated than the cationic ones,^16^ which feeds into whether the Coulombic interactions between (salt) ions and charged groups of proteins involve contact pairs, solvent separated pairs, or no pairing at all.^12,17,18^

A considerable amount of research directed toward answering some of these questions has been carried out. For example, a combination of vibrational sum frequency spectroscopy and lower critical solution temperature (LCST) studies examining the association of cations with carboxylated elastin-like polypeptides reveals a relatively strong association of divalent metals: Zn^2+^ > Ca^2+^ > Ba^2+^ > Sr^2+^ > Mg^2+^ (*K*_a_ = 100–1000 M^–1^, *K*_d_ = 1–10 mM). In contrast, monovalent cations bound more weakly: NH_4_^+^ > Li^+^ > Na^+^ > NMe_4_^+^ > K^+^ > Rb^+^ ≥ Cs^+^ (*K*_a_ = 2.9–13 M^–1^, *K*_d_ = 77–345 mM). These results are in general agreement with the notion that strongly hydrated cations bind more tightly to carboxylates than do weakly hydrated ones.^19^ Using glycine and acetate as models, a slightly different affinity order was observed with X-ray absorption (Na^+^ > Li^+^ > K^+^ > NH_4_^+^).^20^ Interestingly, infrared (IR) studies do however suggest that different metal cations bind to the carboxylate group via different modes,^21^ and the idea of different modes of binding may explain why the cation-triggered assembly of deep-cavity cavitands suggests a different affinity for carboxylates: Cs^+^ > Rb^+^ > Li^+^ > K^+^ > Na^+^.^22^

Cations also form ion-dipole interactions with amide carbonyl dipoles. Thus, using a simple amide as a surrogate, the Cremer group has identified an affinity ordering of Ca^2+^ > Mg^2+^ > Li^+^ > Na^+^ ≈ K^+^, but even the strongest of these interactions is apparently weak: approximately 0.12 M^–1^ (*K*_d_ = 8.5 M).^23^ Other work suggests more substantial ion-dipole NCIs. For example, a computational evaluation by the Jungwirth group concluded that Na^+^ bound more strongly to the amide carbonyl of *N*-methylacetamide than K^+^,^24^ while a combination of molecular dynamics (MD) simulations and conductivity measurements suggests that Na^+^ binds at least twice as strongly to a protein surface than K^+^.^25^ Context does appear to be key here; on the surface of a protein, chelation by two or more amide carbonyls is a strong possibility. Moreover, a combination of IR spectroscopy and computational studies suggest that, at least for higher valent metal ions, the counteranion plays a key role in the cation-carbonyl NCI.^26^

Anions tend to display much stronger Hofmeister effects,^2−4^ an observation that meshes with the wider supramolecular repertoire for anions binding to proteins. Thus, three general NCI types can be envisioned: their Coulombic interactions with cationic groups; their ability to function as hydrogen bond acceptors for amide N–H and C_α_–H groups; and their proclivity to bind to nonpolar patches. Because of the nature of lysine, arginine, and histidine residues, Coulombic NCIs with anions inevitably also involve hydrogen bonding,^27^ and MD simulations employing both nonpolarizable and polarizable force fields indicate that small anions such as fluoride exhibits strong affinity to the charge groups in the order guanidinium > imidazolium > ammonium and that any affinity of larger halides for each cation is marginal. However, for guanidinium, there are many open questions about the preference for anion binding to the face or edge of the cation^28^ and how the bidentate binding of monatomic anions to the edge of guanidiniums energetically compares to paired hydrogen bonds in, for example, guanidinium–perchlorate interactions.^29^ While MD studies suggest that smaller halides preferentially bind to cationic residues, similar MD studies suggest that larger halides preferentially bind to *tetra*-alkylammonium cations.^30^

In regard to amide N–H and C_α_–H groups as hydrogen bond donors for anions, MD studies suggest that there is little, if any, appreciable interaction with small models such as *N*-isopropylacrylamide and *N*-methylacetamide.^24,31^ Again, however, this is apparently strongly context dependent. Thus, Nest and C_α_NN motifs are common anion binding sites in solid-state protein structures, emphasizing that chelation by multiple amide N–H leads to more significant affinity.^32−34^ Hence, in contrast to small amide models, anion hydrogen bonding to the amide N–H groups of peptides is apparently stronger^35^ and also contributes to the multiple mechanisms by which physical properties such as the LCST of polyamides can be affected.^36,37^

Analogous studies with poly(*N*,*N*-diethylacrylamide) devoid of N–H moieties reveal that formal hydrogen bond donors are not a requirement to observe anion-induced changes in LCST^38^ and point to the importance of anion binding to nonpolar patches in Hofmeister effects. For example, while little, if any, interactions were noted between anions and amide N–H groups of small models such as *N*-isopropylacrylamide and *N*-methylacetamide,^24^ charge-diffuse anions do bind to the nonpolar groups of these small molecules. Furthermore, anion binding to the nonpolar patches in proteins has also been observed,^39^ and there is a growing body of work reporting anion complexation to synthetic hosts possessing nonpolar pockets.^40−47^ There is much to learn here about these nearly ubiquitous NCIs. However, it is evident that key to these interactions is the weak hydration of nonpolar surfaces,^48−52^ hydration that weakens further with increasing surface area^53^ and negative curvature (concavity).^54,55^

In most systems, multiple NCIs lie behind the observed Hofmeister effects. For example, a combination of anion hydrogen bonding to amide N–H groups and anion binding to nonpolar concavities leads to changes in LCST,^36,37^ and a combination of cation binding to amide carbonyls and anion binding to the apolar surface of model polypeptides can explain the experimentally observed salt effects on their activity.^56^ Multiple NCIs have also been shown to affect triglycine^35^ and in particular its salting-out constants.^57^ Consequently, it can be difficult to parse out and evaluate all of the different NCIs that contribute to Hofmeister effects.

**Figure 1:**
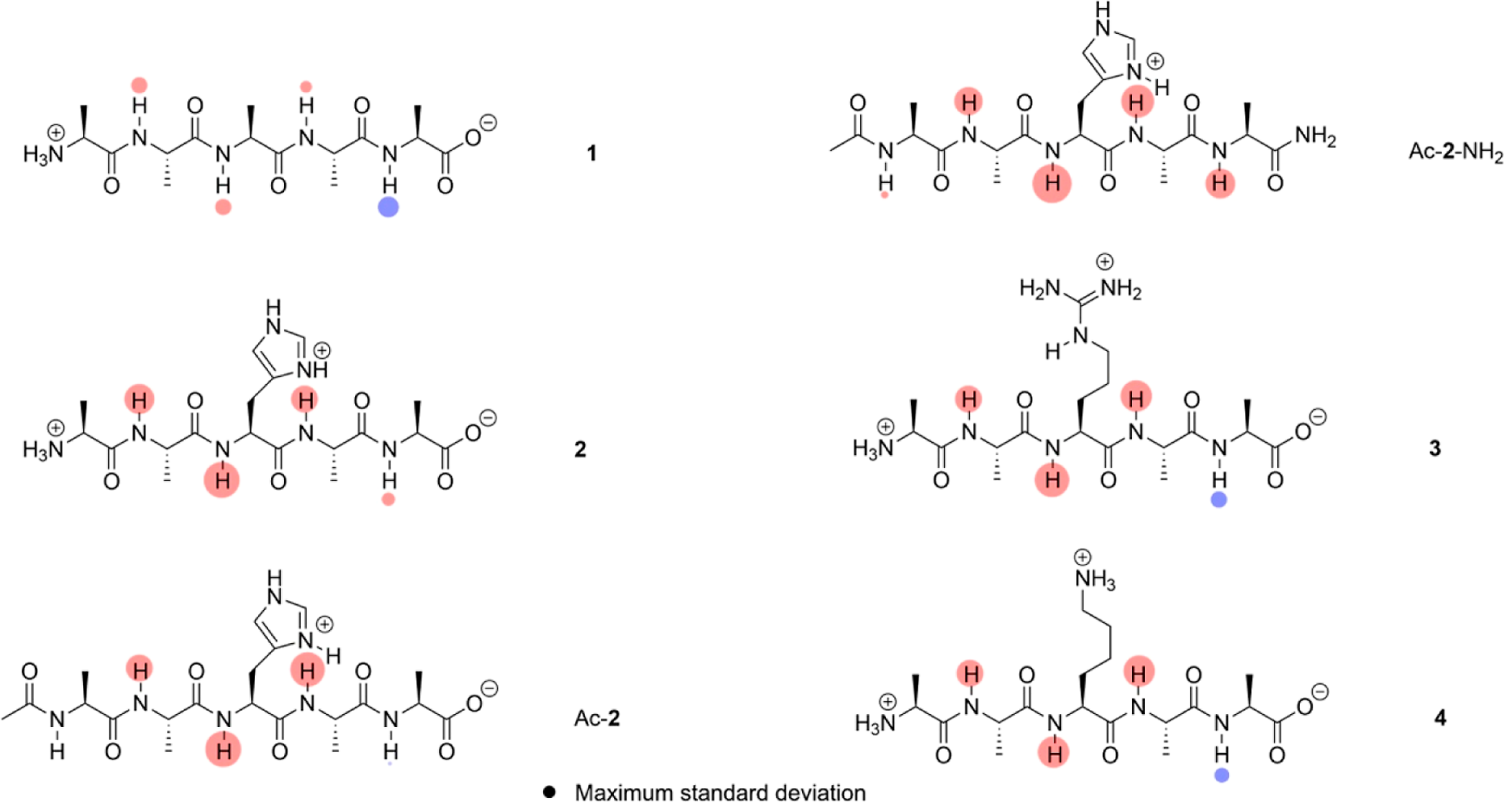
Unreferenced response (Δδ) of amide N–H ^1^H NMR signals upon the addition of NaClO_4_ to give a final salt concentration of 250 mM. The areas of the circles associated with each N–H group in structure **2**–**4** are proportional to the ^1^H NMR signal shift, with upfield signals shown in red and downfield shifts shown in blue. Where shifts are small, the circle is shown above or below the H atom. Represented at the same scale, the largest standard deviation derived from data triplication is shown at the foot of the figure. Full (referenced) data are presented in Table S2 (see Supporting Information, Section S8).

Previously, we reported on anion binding to the protein ubiquitin (Ub), observing six binding sites at organized and exposed locations on the surface of the protein.^58^ These resulted in either a salting-in or a salting-out (reverse Hofmeister) effect depending on the pH. The binding sites were themselves located using ^1^H ^15^N HSQC nuclear magnetic resonance (NMR) spectroscopy experiments by tracking the amide N–H signals as a function of added anion. However, in subsequent further studies, we have noted complications with monitoring N–H signals in Ub. Indeed, NMR spectroscopy monitoring of anion binding to amide N–H groups in general is complicated by a number of factors. First, these signals cannot usually be monitored at pH ∼ 7 because the amide protons exchange with solvent on or near the NMR spectroscopy time frame. As a result, signals are frequently unobservable. Second, as Hofmeister effects involve weak NCIs, signal shifts are typically relatively small (around or less than 0.1 ppm). Third, in titration experiments with salts, the ionic strength of the solution changes. This can cause magnetic susceptibility signal shift difficulties^59^ and introduces screening effects, which may or may not affect affinity determinations.^60^ Fourth, to our knowledge, the cumulative effect of forming N–H···X^–^ hydrogen bonds at the expense of either N–H···OH_2_ or N–H···O=C hydrogen bonds (to water or amide carbonyl groups, respectively) has not been closely assessed. For these reasons, we sought a simple system that could act as a reference for any anion Hofmeister studies reliant on amide N–H signal tracking by NMR spectroscopy. Specifically, we report here NMR spectroscopy as a tool to assess the association of anions to a series of pentapeptides (Figure 1). These hosts were selected for three reasons. First, they are too short to fold and therefore avoid the complicating issue of N–H···X^–^ interactions breaking N–H···O=C hydrogen bonds (unfolding). Second, they are ostensibly large enough to display measurable anion affinities; i.e., they represent minimal structures where N–H···X^–^ hydrogen bonding and anion-binding to nonpolar sites should be observable. Third, we wished to explore how anion complexation varied as a function of changes to the central (third) amino acid and termini capping and hence changes in the net charge. Our results not only provide useful information about NMR spectroscopy as a tool for anion binding to peptidic systems and the subtle signal shifts that generally occur, but also suggest alternative ways to assess systems when associations are weak and difficult to quantify.

## Results and Discussion

Six pentapeptides (Figure 1) were selected: AAAAA (**1**), AAXAA [X = His (H), Arg (R), and Lys (K): respectively, **2**–**4**], Ac-AAHAA (Ac-**2**), and Ac-AAHAA-NH_2_ (Ac-**2**-NH_2_). These were chosen to determine: (1) if anion binding could be observed in a charge-neutral peptide (**1**); (2) the effects of adding a positive charge at residue-3—equidistant from the termini—and whether its nature induced any anion-binding selectivity (**2**–**4**); and (3) in the case of **2**, how removal of the *N*- or *C*-terminal charge affected anion association (Ac-**2** and Ac-**2**-NH_2_). The natural abundance peptides were obtained commercially (GenScript), with mass analysis used as a quality control. All received peptides were passed through a chloride DOWEX resin to ensure uniform counterion, a Biogel P2 desalting column for final purification, and were fully characterized by NMR spectroscopy (^1^H, TOCSY, and ROESY) using solutions of pH = 5.2 (sodium acetate buffer). All spectral characterization data are provided in the Supporting Information (Sections S1 and S2 and Figures S2–S19).

NMR spectroscopic analysis of the peptides at pH = 5.2 allowed not only the identification of all C_α_–H and side-chain signals but also each individual amide N–H signal (Figures S2–S19). To assess anion binding to the different peptides, we focused on these amide signals and the sodium salts of Cl^–^, I^–^, and ClO_4_^–^. These anions were selected to cover a range of charge diffusivities, from “Hofmeister neutral” Cl^–^ to charge diffuse ClO_4_^–^. Each titration experiment involved either 10 or 50 mM acetate as buffer, depending on whether the peptide concentration was 1 mM, or, for two-dimensional (2D) NMR spectroscopy work, 5 mM. These differences in peptide and buffer concentrations led to no perceptible spectroscopic changes.

Because we observed occasional signal overlap when carrying out preliminary ^1^H NMR spectroscopy titration experiments, we performed parallel sets of studies: (1) formal ^1^H ^15^N HSQC NMR titration experiments involving **2** and incremental additions of presumptively relatively strongly binding ClO_4_^–^ (up to 250 mM salt); (2) single-point, ^1^H ^15^N HSQC NMR spectroscopy “titrations”, where the spectra of peptides **1**, **3**, **4**, Ac-**2**, and Ac-**2**-NH_2_ were recorded at 0 and 250 mM ClO_4_^–^; and (3) formal ^1^H NMR spectroscopy titration experiments involving various combinations of all peptides and Cl^–^, I^–^, and/or ClO_4_^–^ (Supporting Information, Sections S3–S5). This combination allowed unambiguous tracking of all amide N–H signals as a function of salt while minimizing time-consuming (and sample consuming) 2D titration experiments where each data point on a titration curve required a ^1^H ^15^N HSQC spectroscopic experiment.

In all cases, it was the amide N–H signals that underwent the largest shifts (Δδ). Figure 1 shows the unreferenced ^1^H NMR response of each amide N–H signal upon the addition of 250 mM ClO_4_^–^. Referenced data for all anions and salts are provided in the Supporting Information (Section S8 and Table S2 and the accompanying figures). In these “bubble diagrams”, the area of each red (upfield shift) and blue (downfield shift) circle is proportional to the Δδ for the signal of the N–H in question. In contrast, the small downfield shifts observed for the terminal Ala5 amide N–H of each peptide are attributed to magnetic susceptibility and other ionic strength change effects.

As the bubble patterns for the different peptides show, the Δδ values indicate that replacing N–H···OH_2_ hydrogen bonds with N–H···X^–^ interactions induces small but significant (cf., e.g., ref (53)) upfield shifts in an amide N–H signal (maximal Δδ = 0.062 ppm). Using either an external reference (3-(trimethylsilyl)propane-1-sulfonate) or the Ala5 methyl signal, these upfield signals were larger (maximum Δδ = 0.103 ppm). However, in terms of qualification, the unreferenced data prove to be the most insightful, demonstrating that even the small upfield shifts induced by anion association are relatively large compared to the downfield shifts induced by magnetic susceptibility/ionic strength effects.

**Figure 2:**
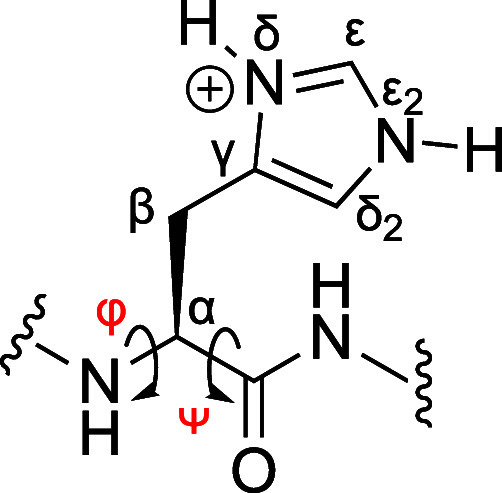
His3 φ and ψ dihedrals (red) and atom labeling in the side chain.

For peptide **1**, the data reveal minimal association of ClO_4_^–^ but an association that was biased toward the *N*-terminus. The addition of a second positive charge (e.g., **2**) enhances the anion association considerably, with anion binding focused on the amide groups either side of His3 but again biased toward the *N*-terminus. Capping the *N*-terminus and removing its charge (**2** versus Ac-**2**) shifts the association away from the *N*-terminus toward His3 and Ala4. This is perhaps to be expected considering the change at the *N*-terminus; however, the fact that there is considerable anion affinity at Ala4, despite the presence of the negative charge on the *C*-terminus, is somewhat surprising. One interpretation of this (but see below) is that it is a reflection of the ease of rotating the His3 ψ dihedral (Figure 2) to allow interaction with both the imidazolium N^+^–H and the amide N–H of Ala4, relative to rotating the His3 Φ dihedral to allow interaction with the imidazolium N^+^–H and the amide N–H of His3, despite the former involving a nine-membered chelate with the anions and the latter involving an eight-membered chelate. A second important point to note is the difference between binding of the anion to **1** and Ac-**2**, both of which have a net zero charge. There is significantly more anion association with Ac-**2**, where the positive charge is in the center of the main chain (and nearer the negative charge) than when the positive charge is at the *N*-terminus (**1**). We attribute the more extensive anion binding in the case of Ac-**2** to two reasons. First, because of the His3 side chain, Ac-**2** has a more extensive nonpolar surface with which charge-diffuse anions interact (see below). Second is the overall position of the positive charge. Thus, the Cremer group has reported how polymers are more weakly solvated in their midsections compared to their termini, which leads to stronger anion association.^53^ This weaker solvation, plus the increased probability of anions being able to simultaneously form multiple NCIs with different parts of the peptide, undoubtedly contributes to the greater anion association to the amide N–H groups of Ac-**2**.

When the negative charge at the *C*-terminus is removed (Ac-**2**-NH_2_ versus Ac-**2**), there is the expected rebiasing of the anion association toward the *C*-terminus. Here, the maximal observed Δδ is at His3, which could be interpreted as suggesting that in this case, the adjustment of the His3 Φ dihedral, to allow association with the anion via an eight-membered chelate, predominates. Overall, however, the differences in the nature of ClO_4_^–^ association to **2**, Ac-**2**, and Ac-**2**-NH_2_ are small and may largely result from subtle (de)shielding effects induced by the His3 side chain (see below). However, comparing these three peptides to **1** strongly supports the concept that anion association with central regions of peptides is preferred over binding near the terminus.

Looking at the overall quality of the binding of an anion to peptides **3** and **4** and comparing this to the data for **2**, it is apparent that the nature of the positive charge at residue-3 does not greatly affect the nature of ClO_4_^–^ association. To investigate this further and assess the signal (de)shielding role of the aromatic ring in **2**, we examined the predicted ^1^H NMR signals of the amide N–H groups as a function of the side chain conformation of His3 (Supporting Information, Section S9). Briefly, NMR tensors and magnetic susceptibilities of the N–H signals of different conformations of **2** were calculated with the Gauge-Independent Atomic Orbital (GIAO) method^61,62^ at the B3LYP/6-311+G(2d,p) level (Gaussian 16 package).^63,64^ In these calculations, a polarizable continuum water model was applied, and tetramethylsilane was used as a reference. We examined two dihedrals of His3 in these calculations: N_amide_–C_α_-C_β_–C_γ_ and the C_α_–C_β_–C_γ_–N_δ_ (Figure 2). Varying the former incrementally showed the effects of swinging the imidazole side chain from pointing to the *N*-terminus to pointing at the *C*-terminus. Subsequently, using these two extrema, we also incrementally changed the C_α_–C_β_–C_γ_–N_δ_ dihedral to examine the effect of rotating the imidazole ring relative to the main chain (Supporting Information Section S9 and Figures S60–S63). Variations in the N_amide_–C_α_–C_β_–C_γ_ dihedral led to the largest shifts for the N–H signal of Ala4 (up to ∼1.4 ppm), smaller shifts for Ala2 and His3 (∼0.5 ppm), and smaller shifts for Ala5 (0.3 ppm). Similarly, when the C_α_–C_β_–C_γ_–N_δ_ dihedral angle was changed so that the imidazole side chain pointed at the *N*-terminus, a 1.2 ppm shift was calculated for His3, a 0.6 ppm shift for Ala2, and small shifts for Ala4 and Ala5. Correspondingly, when the imidazole side chain pointed at the *N*-terminus, large shifts were seen for His4 (some unrealistically large because of energetically infeasible conformations) and to a lesser degree for Ala5, and small shifts were calculated for Ala2 and Ala3. Our conclusion here is that if imidazole chelation was important to anion association, then the signal shifts in His3 or Ala4 could be up to 25 times larger than that actually observed. Thus, in line with the similar signal shifts observed when ClO_4_^–^ associates with **2**, **3**, or **4**, the calculated NMR signal shift data suggest that chelation is not a dominant factor assisting anion binding the amide N–H groups. Rather, the association at residues 3 and 4 is best described as being controlled Coulombically; i.e., the electrostatic potential field generated by the side chain of His3/Arg3/Lys3, the increased nonpolar surfaces of these peptides, and the weak solvation of the midsection of the mainchain drive ClO_4_^–^ association.

**Figure 3:**
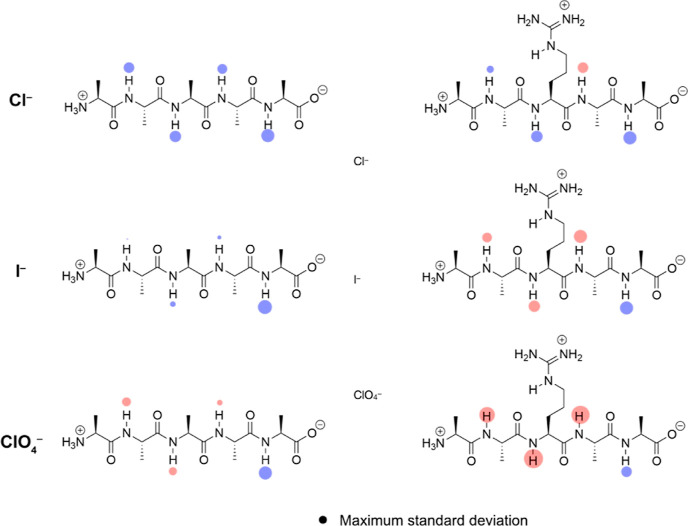
Unreferenced response (Δδ) of the amide N–H ^1^H NMR signals of peptides **1** and **3** upon the addition of Cl^–^, I^–^ or ClO_4_^–^ to give a final salt concentration of 250 mM. The areas of the circles associated with each N–H group are proportional to the ^1^H NMR shift, with upfield signals shown in red and downfield shifts shown in blue. Where shifts are small, the circle is shown above or below the H atom. Represented on the same scale, the largest standard deviation is shown at the foot of the figure. Full referenced data are presented in the Supporting Information (Section S8 and Table S2).

To confirm that ClO_4_^–^ was indeed the strongest-binding guest of the anions studied, we also examined the binding of Cl^–^ and I^–^ to peptides **1** and **3**. Figure 3 shows the corresponding bubble diagrams (the same scale as that used in Figure 1). In all experiments, the magnitude of Δδ for each amide N–H signal followed the order: ClO_4_^–^ > I^–^ ≫ Cl^–^. These data confirm that the association follows the Hofmeister series, with more charge-diffuse, weakly solvated anions associating the most strongly. Thus, in the case of peptide **1** and Cl^–^, only magnetic susceptibility and ionic strength effects are evident in the (downfield) shifts of the amide N–H signals. Even in the case of positively charged **3**, there is only the suggestion of Cl^–^ association with the upfield shift in Ala4. However, with the more charge-diffuse anions, these downfield shifts are replaced with upfield ones induced by anion binding. Thus, in peptide **1**, only ClO_4_^–^ demonstrates any upfield signal shifts suggestive of association, whereas for peptide **3**, there is evidence of I^–^ complexation, and even some suggestion of Cl^–^ complexation to the Ala4 amide.

The aforementioned ^1^H NMR spectroscopic titration experiments involving various combinations of the peptides and salts allowed us to assess whether the observed small signal shifts could be used to quantify anion association. To ensure optimal data fitting, in each determination, the raw data were referenced using the Ala5 methyl signal, which proved to be as suitable as an external reference as 3-(trimethylsilyl)propane-1-sulfonate. The affinities were expected to be low, and fitting the titration data to a 1:1 model proved that this was indeed the case (Sections S5–S8). Thus, for more strongly associating ClO_4_^–^, affinities range from 0.12 to 1.92 M^–1^, with errors ranging from 12 to 150% (Supporting Information, Section S8 and Table S1). The problem here is the tension between keeping ionic strength effects as low as possible and the necessary large excesses of salt added because of the weak intrinsic affinity. This meant that most titrations were terminated at 30–40% host complexation, far below the ideal >80% level required to minimize fitting errors. This highlights the fact that affinity constant determinations can be of limited utility when examining Hofmeister effects.

**Figure 4:**
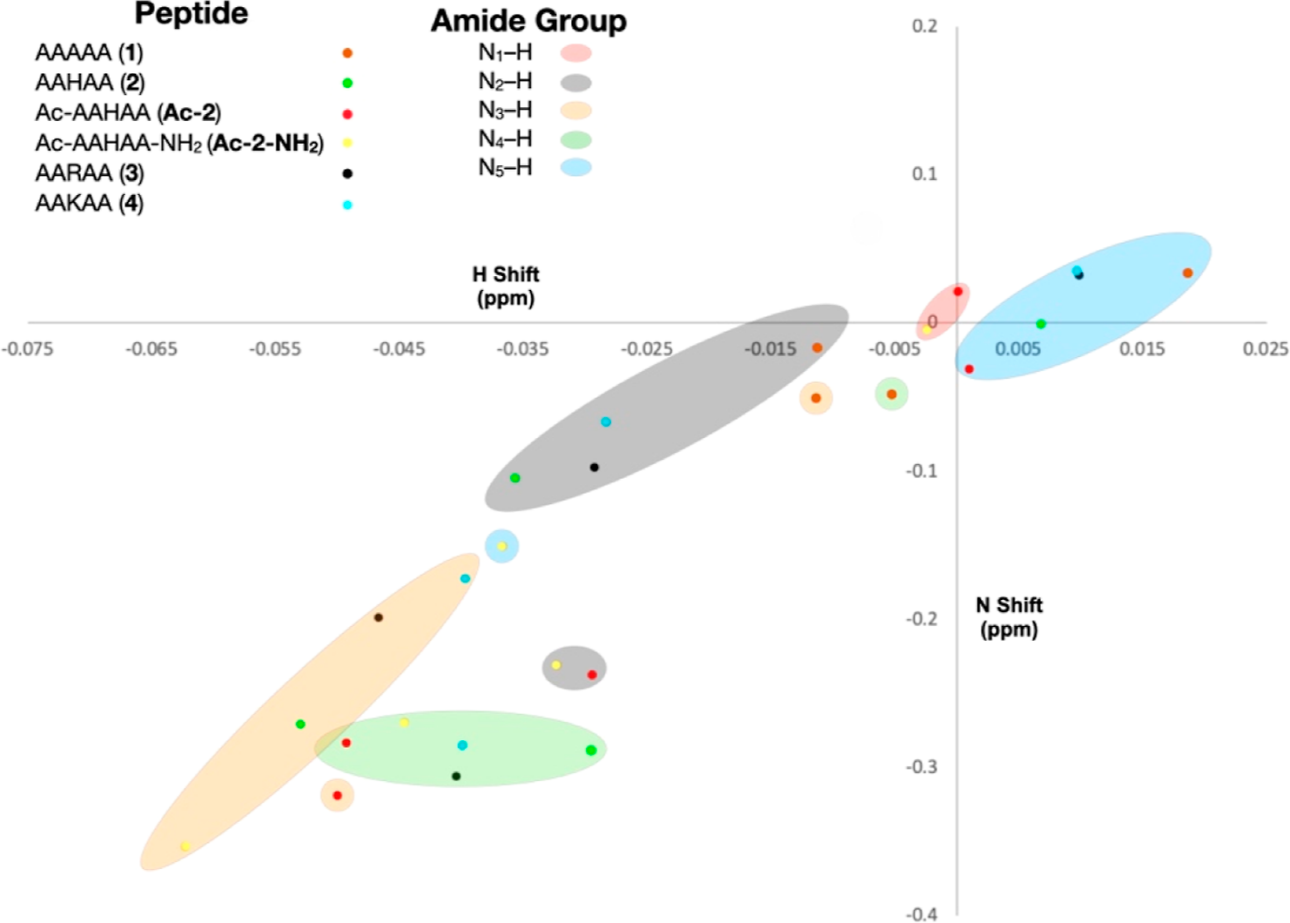
Unreferenced response (Δδ) of the amide N–H signals of the six peptides using ^1^H ^15^N HSQC NMR upon the addition of ClO_4_^–^ to give a final salt concentration of 250 mM. Full data (with errors) are presented in the Supporting Information (Section S4 and Figure S26–S31).

Figures 1 and 3 show binding of the anion to the six peptides, as reported by the individual amide N–H groups along the H-dimension. We were also interested in assessing anion association using the N-dimension. Figure 4 shows the Δδ values from the amide N–H groups of each peptide using ^1^H ^15^N HSQC NMR spectroscopy. This plot reveals a clear correlation between signal shifts in the H- and N-dimensions, how the amide N–H of Ala5 generally undergoes a distinctive downfield shift because of magnetic susceptibility and ionic strength effects, and the much larger upfield shifts of the amides of resides 3 and 4. However, the percentage shifts along the N-dimension are small, and correspondingly, the errors are relatively large (Supporting Information, Section S4 and Figures S26–S31). This suggests that the changes in the electron density around the N atoms upon anion association are small compared to those of the corresponding H atoms. In other words, although ^1^H ^15^N HSQC NMR experiments are often necessary for the characterization of the peptides themselves, at least in the case of simple peptides, the N-dimension is not the best reporter for the characterization of anion complexation.

Although the H-dimension is more informative in assessing the anion association with peptides, it is important to note that the observed shifts (Δδ) are relatively small compared to macrocyclic hosts that bind anions via arrays of amide groups. To pick one example from the Hamilton group,^65^ anion binding can induce signal shifts of up to 1.00 ppm, 16 times that reported here. However, large signal shifts do not necessarily correlate with strong binding, and considering previous small molecule studies suggesting charge-diffuse anions associate more with nonpolar groups than with amides N–H groups,^24^ we considered the possibility that the signal shifts in peptides **1**–**4** were small because their amides N–H groups indirectly interact with anions.

To examine anion binding in more detail, we turned to explicit water model MDs simulations of peptides **1**–**4** (AAXAA: X = A, H, R, and K) in the presence of excess NaCl or NaClO_4_. The simulations were performed in bulk water at 25 °C and 1 bar, with the peptides modeled using the Amber-ff03 all-atom force field,^66,67^ the ions modeled using the generalized Amber force field^68^ and their partial charges obtained from AM1-BCC calculations,^69^ and the water modeled using TIP4P2005.^70^ The net charge on the peptides containing a basic residue was set to +1 to represent their protonation state at pH 5.2, while **1** was modeled in its neutral zwitterionic form. Each simulation included 1 peptide and 33 anions in a bath of 3000 water molecules. All simulations were run in the isothermal–isobaric ensemble, with the temperature and pressure maintained using the Nosé–Hoover thermostat^71,72^ and the Parrinello–Rahman barostat.^73^

**Figure 5:**
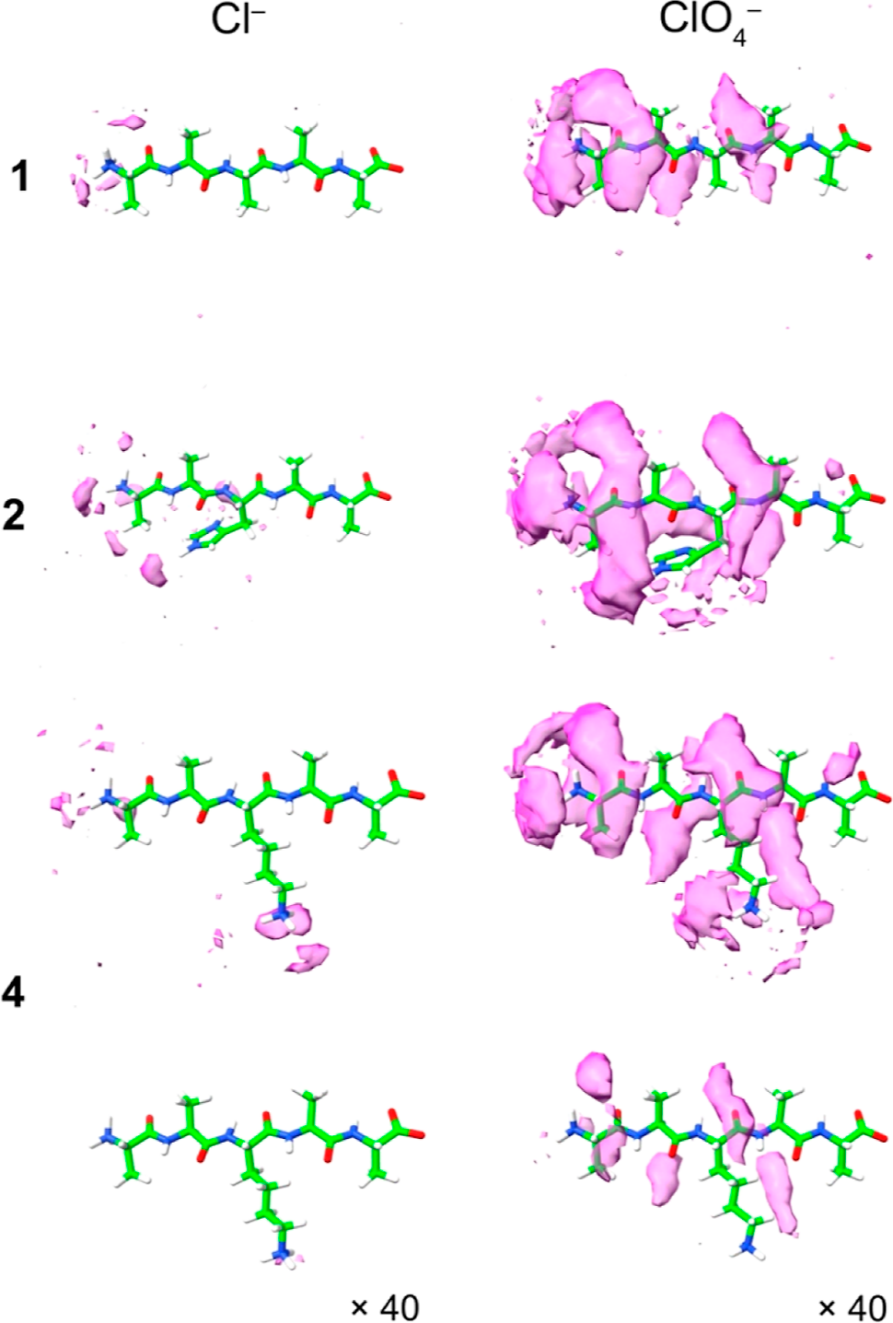
Spatial distribution functions revealing anion association to peptides **1**, **2**, and **4**. All anionic “clouds” (magenta) correspond to probability thresholds set to 12× (or, if indicated, 40×) bulk density. The left-hand and right-hand columns show the zones of high probability for finding Cl^–^ and ClO_4_^–^, respectively. Details of the simulations and data for a second conformation of **2** (with the imidazole ring pointing toward the *C*-terminus) and peptides Ac-**1**, Ac-**2**, and **3** are presented in the Supporting Information (Section S9).

Figure 5 shows the anion trajectories from the simulations, extracted and rendered as volumes—spatial distribution functions—using TRAVIS^74,75^ and visualized using ChimeraX.^76^ Specifically, the anionic “clouds” (magenta) correspond to probability thresholds set to 12 times bulk anion density (or 40 times in the indicated lower two images). These figures reveal enhanced anion accumulation on the surface of the peptide when it contains a second positive charge, the depletion of anion at the C-terminus of each peptide, and, most graphically, the significantly greater anion accumulation in the case of charge-diffuse ClO_4_^–^. Importantly, the lower two figures showing the probability thresholds set to 40 times the bulk anion distribution emphasize that ClO_4_^–^ association is focused on the “channels” between either adjacent methyl groups (Ala1–Ala2) or a methyl group and the adjacent amino-butyl side chain of Lys (Ala2–Lys3 or Lys3–Ala4). Thus, as is observed with simple amides, the calculations suggest that charge-diffuse anions preferentially bind to the nonpolar moieties of peptides rather than function as hydrogen bond acceptors for the amide N–H groups. This idea ties with the relatively small shifts in amide N–H NMR signals observed (Figure 1) as well as the limitations of using the N-dimension; the amide groups are indirect reporters of anion association.

## Conclusions

Although the anion affinity to the studied pentapeptides is low and susceptible to large errors, the H-dimension of NMR spectroscopy proves to be a useful tool to qualify the nature of anion affinity. This is much less so in the case of the N-dimension. As the bubble diagrams demonstrate (Figures 1 and 3), anion affinity is generally focused on the center of the peptides and, intuitively, is enhanced by the addition of positive charge. However, no ion–ion specificity is observed. Signal shifts associated with anion binding to these unfolded peptides are upfield and considerably larger than the downfield shifts associated with magnetic susceptibility and other ionic strength change effects. Nevertheless, the observed upfield shifts are small compared to those induced by anion binding to amide-based hosts. MD simulations suggest why this is so. Thus, charge-diffuse anions associate mostly with the nonpolar regions of the peptide rather than directly interacting with the amide N–H groups.

These studies reveal the utility of ^1^H NMR spectroscopy to qualify anion-binding to peptides even when affinity constants are very low. The presented work also serves as a benchmark for using NMR spectroscopy to study anion binding to more complex peptides and proteins. Studies with such peptidic systems are ongoing and will be reported in due course.

## Supporting Information Available

Details of all peptide characterizations using NMR spectroscopy, spectra and data from the 1D and 2D NMR spectroscopic titration experiments, binding constant analysis, and simulation details (PDF)
