# Supplementary material for: Assessing Weak Anion Binding to Small Peptides
Source: J Phys Chem B. 2024 Apr 9;128(15):3605–13. doi: 10.1021/acs.jpcb.4c00657 (PMC11033870; doi:10.1021/acs.jpcb.4c00657)
Supplement: Supplementary file 1 — jp4c00657_si_001.pdf [file jp4c00657_si_001.pdf]

Supporting Information for:

Assessing Weak Anion-binding to Small Peptides

Corinne L. D. Gibb, Thien H. Tran, Bruce C. Gibb*

Department of Chemistry
Tulane University School of Science and Engineering
New Orleans, LA 70118, USA

Corinne L. D. Gibb orcid.org/0000-0002-2985-6799
Thien H. Tran orcid.org/0009-0001-9359-5829
Bruce C. Gibb orcid.org/0000-0002-4478-4084

*Corresponding author

Table of Contents

1) General NMR Procedures	S3
2) Peptide Characterization	S5
3) 2D NMR Titration Data for Peptide **2** with Perchlorate	S15
a) Analysis of 2D titration with peptide **2**	
b) Referencing the NMR data	
4) Single point 2D titrations for peptides **1**, Ac-**2**, Ac-**2**-NH_2, **3** and **4**	S21
5) 1D Titrations of all Peptides with Perchlorate	S24
6) Fitting of 1D Titration Data	S29
7) Other Anion Binding to Peptides **1** and **3**	S33
8) K_a and Δδ Data Summary and Analyses	S35
9) Pentapeptide spatial distribution function (SDF) calculations and visualization	S40
a) Spatial distribution functions	
b) NMR prediction for AAHAA	
c) Electrostatic potential calculations	
10) References	S48

1) General NMR Procedures

a) Material and instrumentation

All sodium salts were purchased from Aldrich (purity > 99%) and were used without further purification. Peptides were purchased from GenScript with mass analysis as quality control. All peptides were passed through a chloride DOWEX resin to assure uniform counter ion, and through a Biogel P2 desalting column for final purification. All solutions were prepared in ultra-pure water (resistivity of 18.2 MΩ·cm at 25 °C). Each peptide was structurally characterized by NMR spectroscopy (Bruker 700 MHz spectrometer, Louisiana State University). NMR was also used to confirm purity. Where possible, ^{1}H NMR titration experiments were performed on a Bruker 400 MHz instrument (Tulane University). For titrations where better resolution was required, or where ^{1}H-^{15}N- HSQC NMR titrations were necessary, the Bruker 700 MHz instrument was utilized. All data was analyzed using Microsoft Excel.

b) Solution preparation

All peptide solutions were prepared as a 10 mM stock in 9:1 H_2O-D_2O. Final peptide solutions were prepared at 5 mM (for 2D analyses) or 1 mM solution (1D work) in either 50 mM acetate buffer (pH 5.2), or 10 mM acetate buffer, and adjusted as necessary using HCl or NaOH. Stock solutions of 2 M sodium salts were freshly made in 50 mM acetate buffer or 10 mM acetate and pH adjusted to 5.2 as required.

c) Peptide characterization

All peptides were of natural abundance ^{15}N (unlabeled) and were characterized using a combination of ^{1}H (400 MHz), TOCSY, and ROESY (700 MHz) NMR. All experiments utilized a water suppression sequence (ZGESQG). TOCSY and ROESY NMR utilized the respective sequences DIPSI2ESGPPH and ROESYESGPPH.

d) Titration Experiments

1 mM solutions (450 µL) of peptides were titrated directly in an NMR tube using a solution of 2 M sodium salt. A typical titration consisted of 11 spectra, from 0 to 66 µL of salt solution added (final salt concentration typically up to 250 mM). Most 1 mM titrations were run on a 400 MHz. The sample was locked using the zero-salt sample, and the lock fixed during titration. After each aliquot the sample was tuned, the shims optimized, the pulse width calibrated, and the spectrum collected (32 scans). 2D titrations were performed by running ^{1}H-^{15}N- HSQC (sequence FHSQCF3GPPH) at 700 MHz; 16 × 128 scans at low salt concentrations, up to 64 × 128 at high salt concentration.

e) Binding Constant Determinations and NMR Referencing

Where binding constants were sought, spectra were referenced to remove the effect of ionic strength changes during the titration. In these cases, the lock of each sample was not adjusted after each salt addition. Initial studies used sodium 3-(trimethylsilyl)propane-1-sulfonate (DSS) as a reference (0.2 mM) revealing that the *C*-terminal alanine methyl shifted to the same extent as the DSS signal (max 0.001 ppm difference). Consequently, all studies utilized the *C*-terminal alanine methyl as an internal standard. Random checks in different titrations in the presence of DSS revealed no anomalies.

After data collection, all 1D titrations were thus analyzed with referenced to the *C*-terminal alanine methyl group (Eq. S1):

$$\Delta\delta_{(250\ mM)} = \delta_{(250mM)} - \delta_{(0mM)} - (\delta_{Me\text{-}5A(250mM)} - \delta_{Me\text{-}5A(0mM)}) \qquad \text{Eq. S1}$$

where $\Delta\delta_{(250\ mM)}$ is the quoted signal shift ($\Delta\delta$) at the highest salt concentration of 250 mM, $\delta_{(250mM)}$ and $\delta_{(0mM)}$ are the signal positions at 250 and 0 mM salt respectively, and $\delta_{Me\text{-}5A(250mM)}$ - $\delta_{Me\text{-}5A(0mM)}$ are the signal positions for the 5-Ala methyl sidechain at 250 and 0 mM salt respectively. For 2D titrations experiments, signal referencing was not utilized.

f) Binding Constant Determinations and Data Fitting

The plots of referenced proton shift ($\Delta\delta_{(250\ mM)}$) versus concentration of added salt were fitted according to a 1:1 binding model, using the exact solution of the quadratic.[1, 2] Solver in Excel was used for the fitting.

g) Binding Constant Determination Error analysis

For 1D titrations, the error in referenced single point amide proton chemical shift change ($\Delta\delta_{(250\ mM)}$) was obtained from data triplication (minimum), with standard deviations presented. For 2D titrations, experiments were performed in at least triplicated, the raw (unreferenced) signal shift data averaged, and the standard deviation calculated.

2) Peptide Characterization

Figure S1 shows the structures of the six peptides studied.

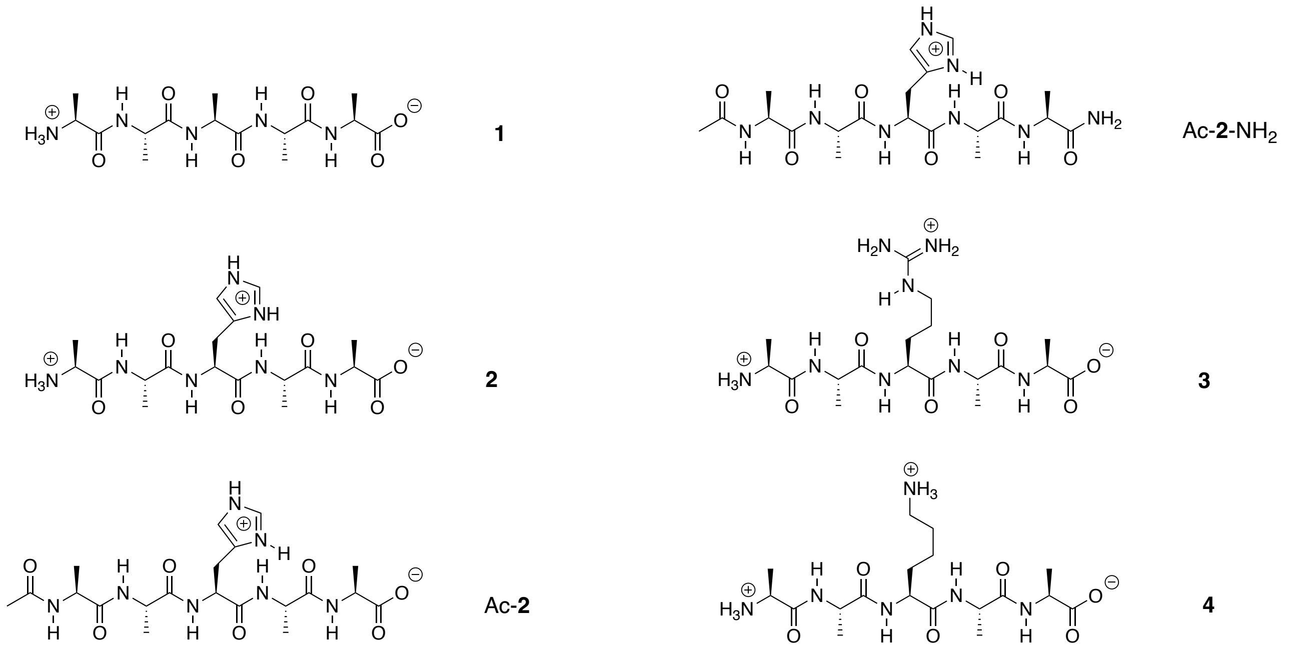

Figure S1: Peptide sequences under investigation.

Figures S2-S18 show the ^{1}H, TOCSY, and ROESY NMR spectra for the peptides shown in Figure S1. Figure S19 shows the amide N–H regions of the ^{1}H NMR spectrum of each peptide.

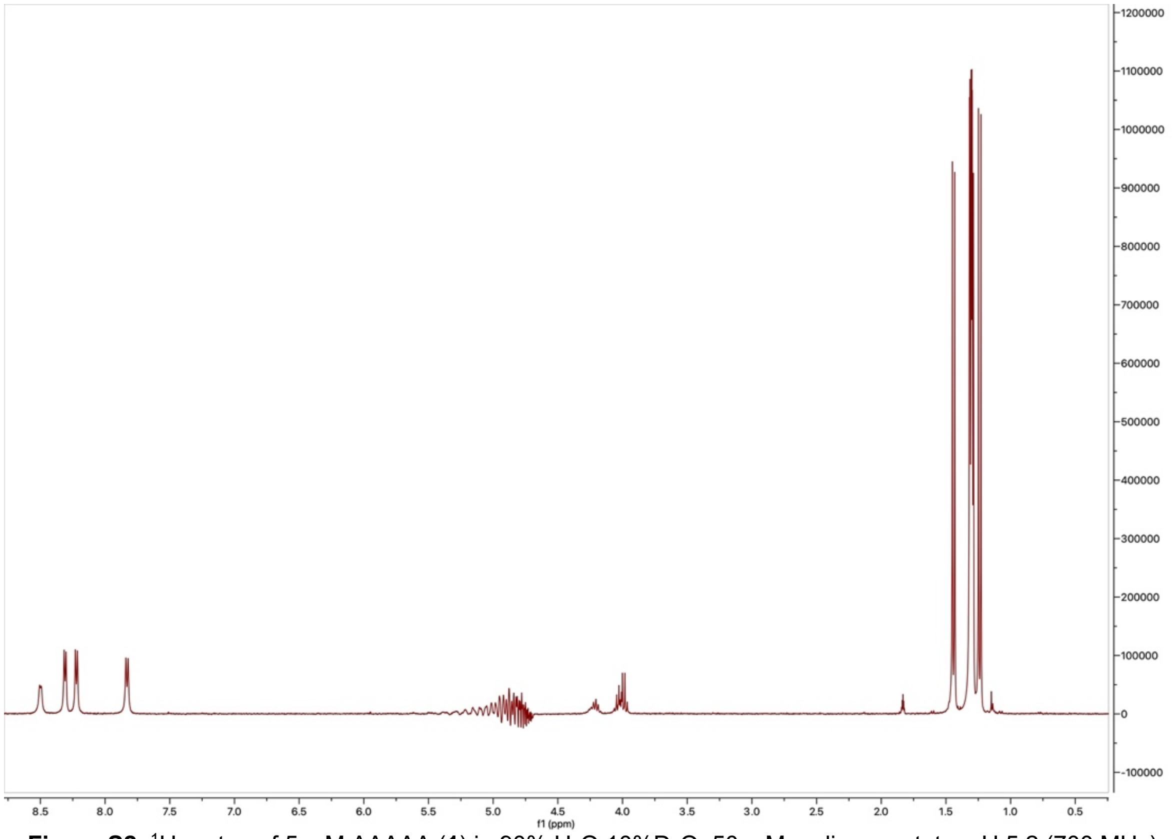

Figure S2: ^{1}H proton of 5 mM AAAAA (**1**) in 90% H_2O:10%D_2O, 50 mM sodium acetate, pH 5.2 (700 MHz).

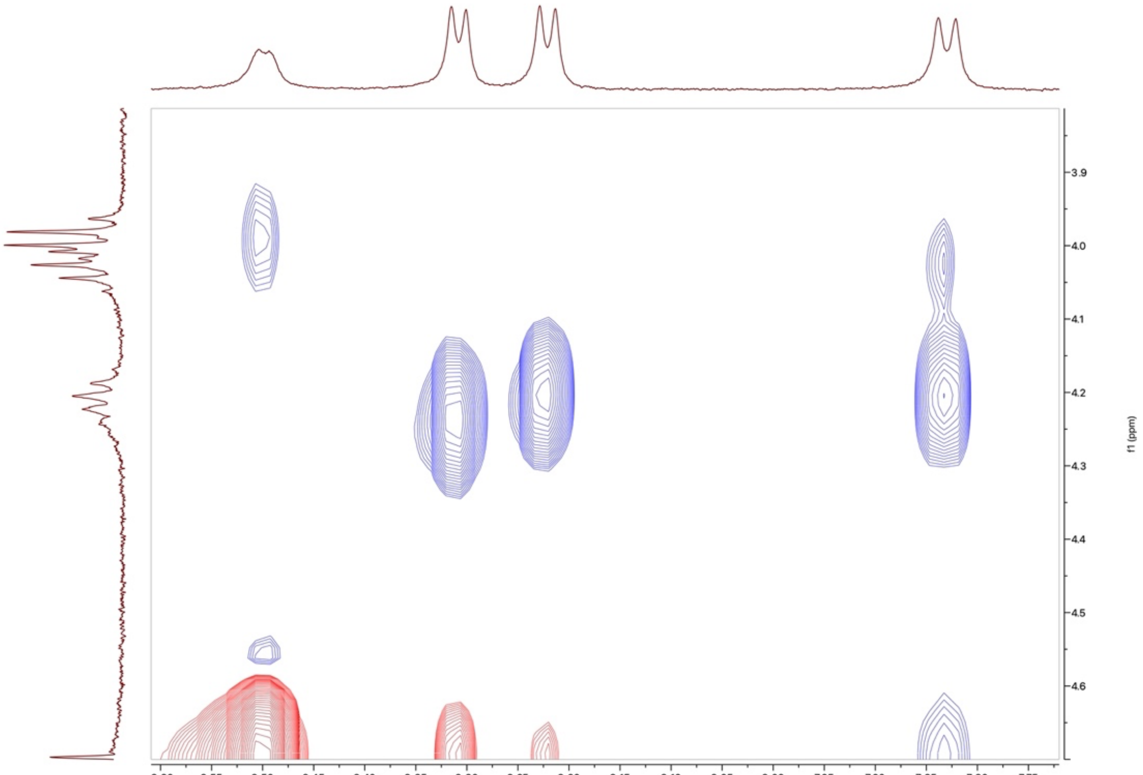

Figure S3: ROESY of 5 mM AAAAA (**1**) in 90% H_2O:10%D_2O, 50 mM sodium acetate, pH 5.2 (700 MHz).

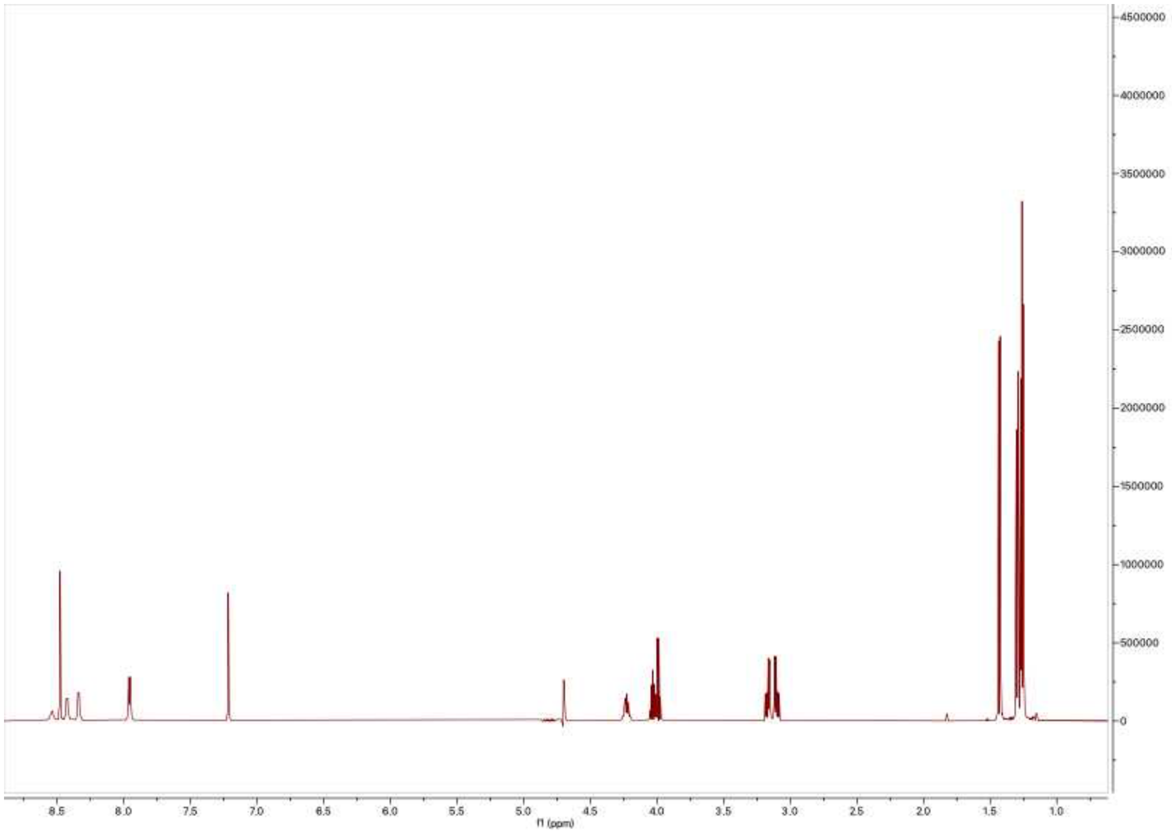

Figure S4: ^1H proton of 5 mM AAHAA (**2**) in 90% H_2O:10%D_2O, 50 mM sodium acetate, pH 5.2 (700 MHz).

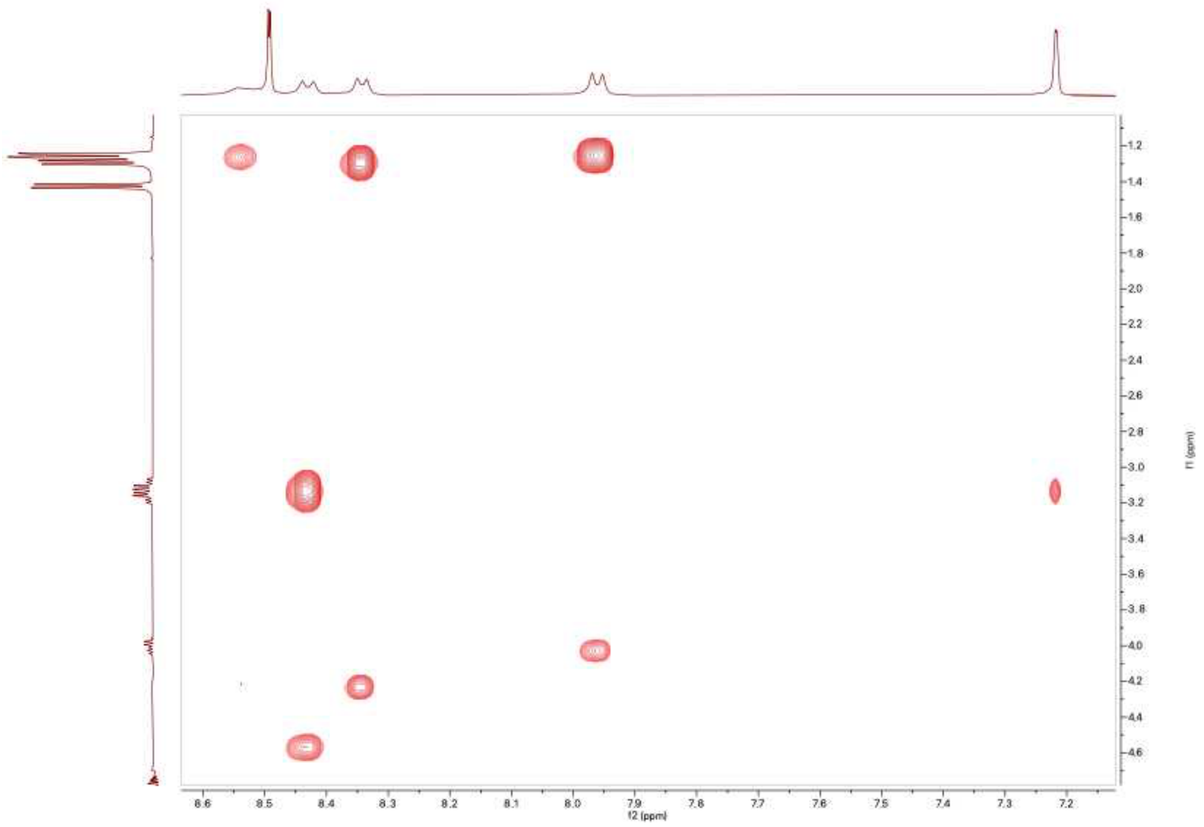

Figure S5: TOCSY of 5 mM AAHAA (**2**) in 90% H_2O:10%D_2O, 50 mM sodium acetate, pH 5.2 (700 MHz).

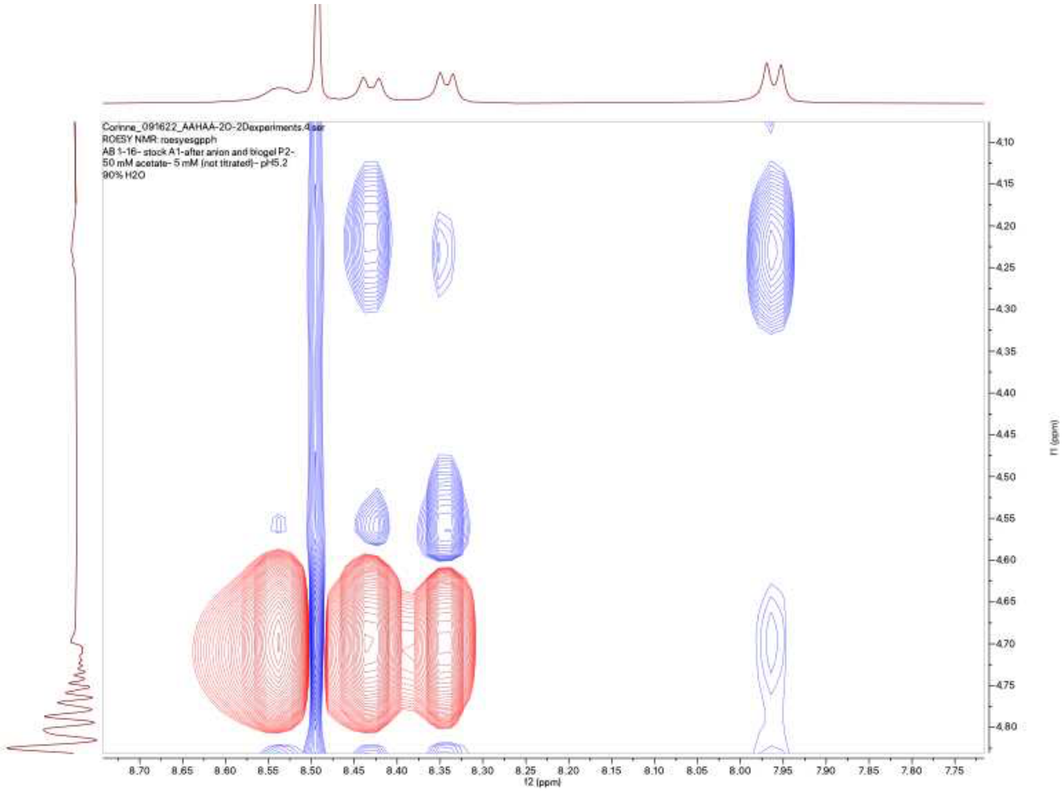

Figure S6: ROESY of 5 mM AAHAA (**2**) in 90% H_2O:10%D_2O, 50 mM sodium acetate, pH 5.2 (700 MHz).

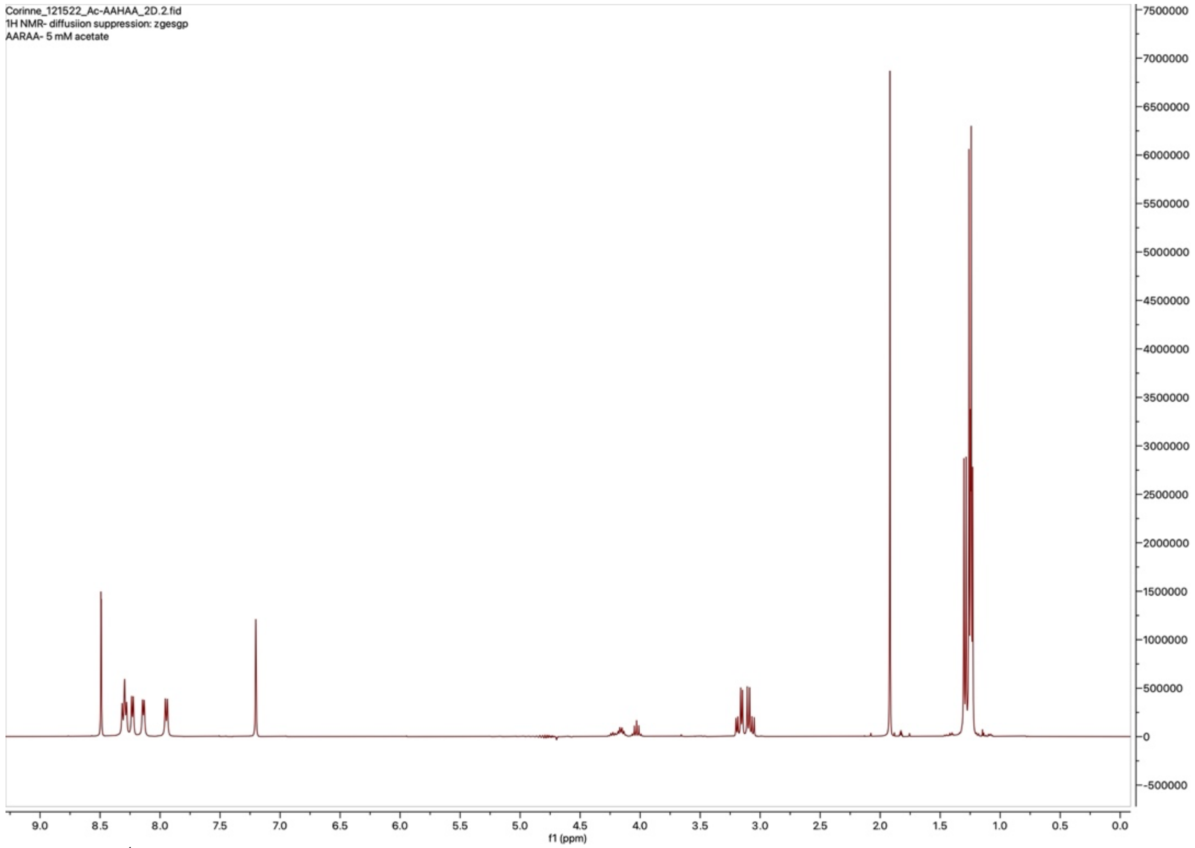

Figure S7: ^{1}H proton of 5 mM Ac-AAHAA (**Ac-2**) in 90% H_2O:10%D_2O, 50 mM sodium acetate, pH 5.2 (700 MHz).

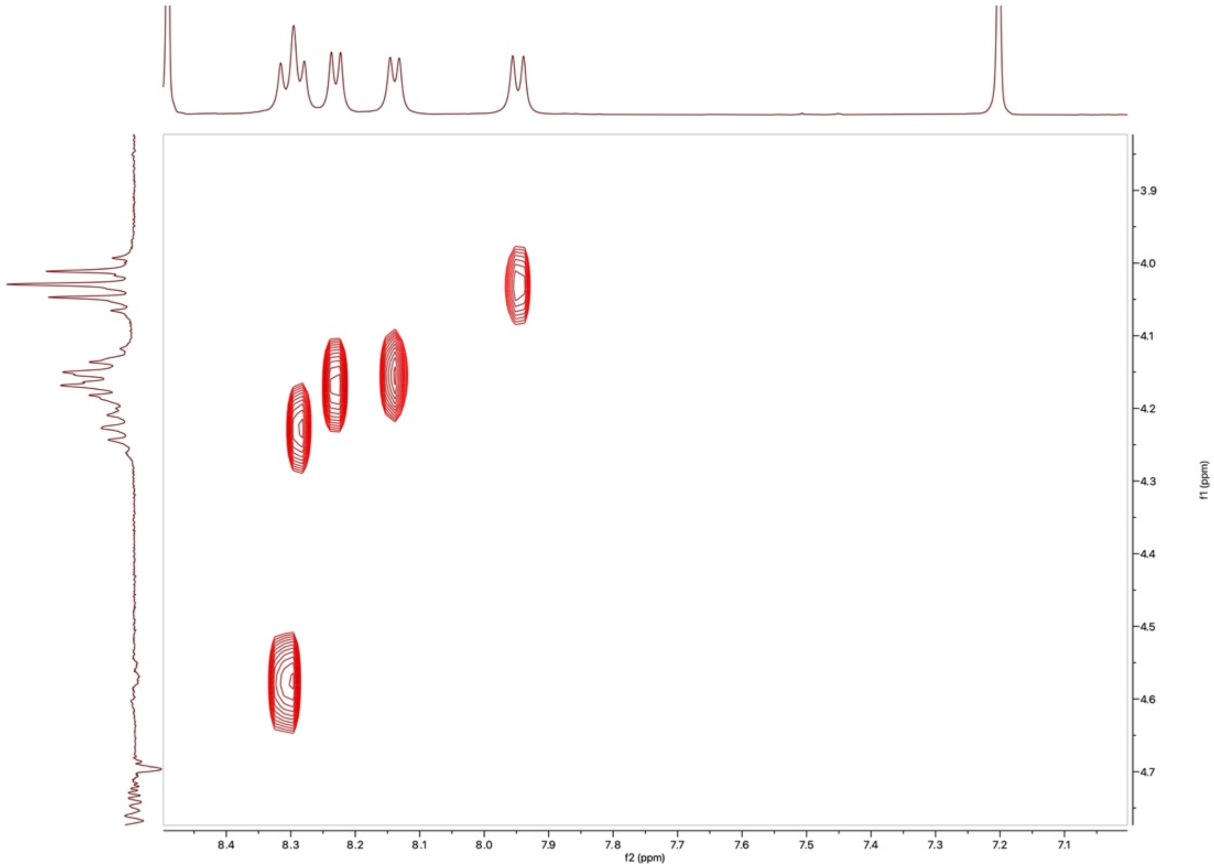

Figure S8: TOCSY of 5 mM Ac-AAHAA (**Ac-2**) in 90% H_2O:10%D_2O, 50 mM sodium acetate, pH 5.2 (700 MHz).

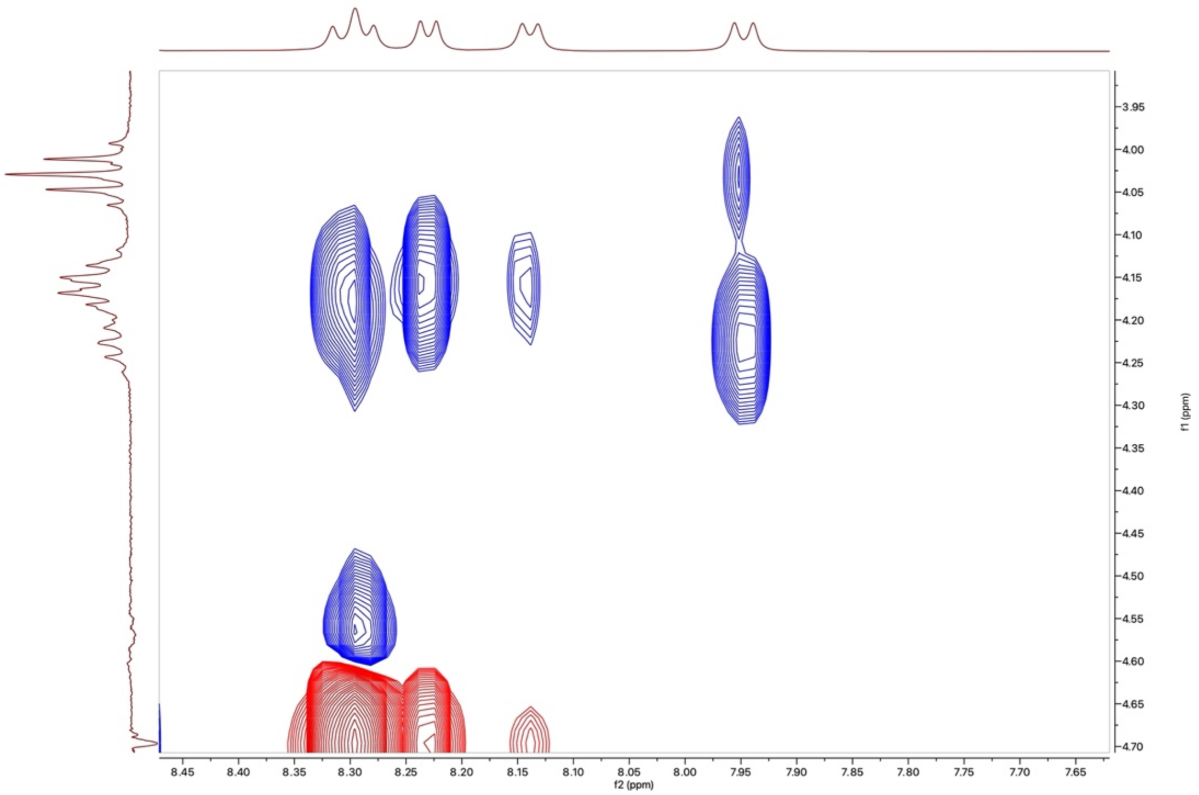

Figure S9: ROESY of 5 mM Ac-AAHAA (**Ac-2**) in 90% H_2O:10%D_2O, 50 mM sodium acetate, pH 5.2 (700 MHz).

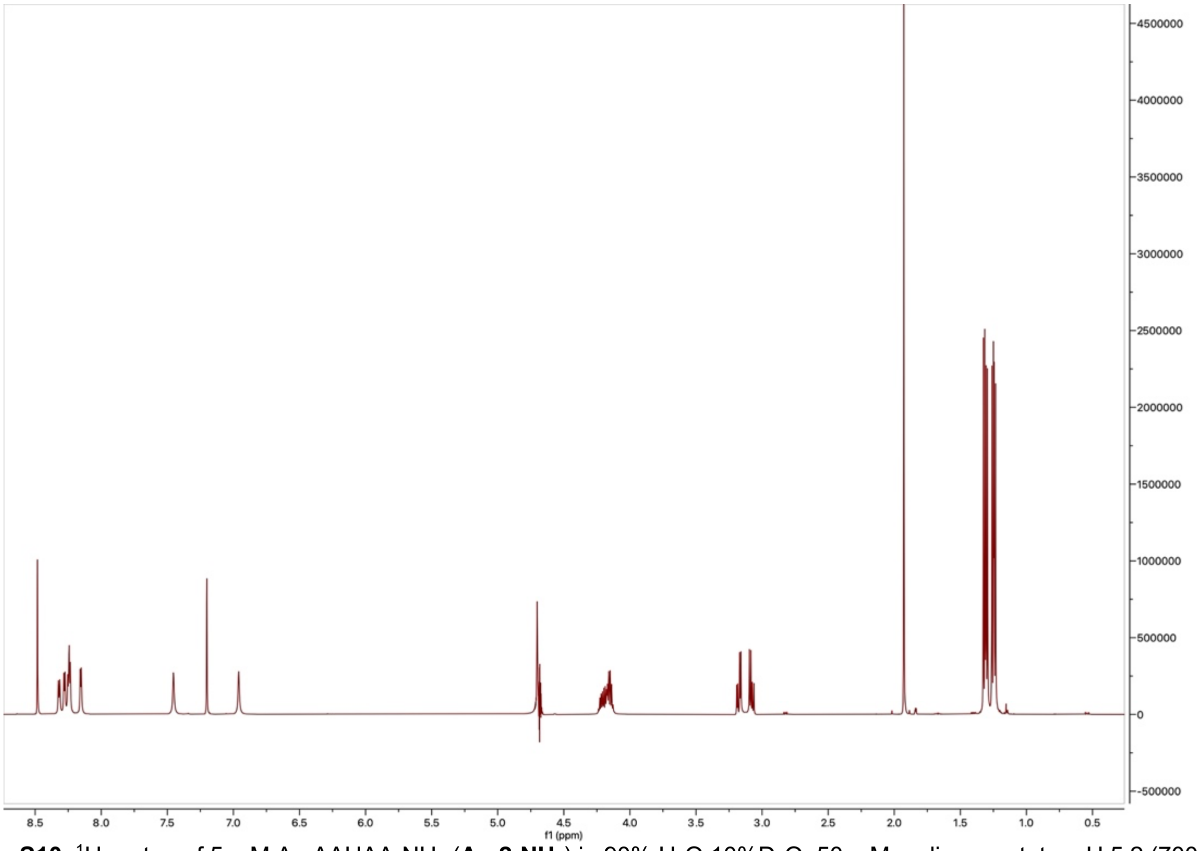

Figure S10: ^{1}H proton of 5 mM Ac-AAHAA-NH_2 (**Ac-2-NH$_2$**) in 90% H_2O:10%D_2O, 50 mM sodium acetate, pH 5.2 (700 MHz).

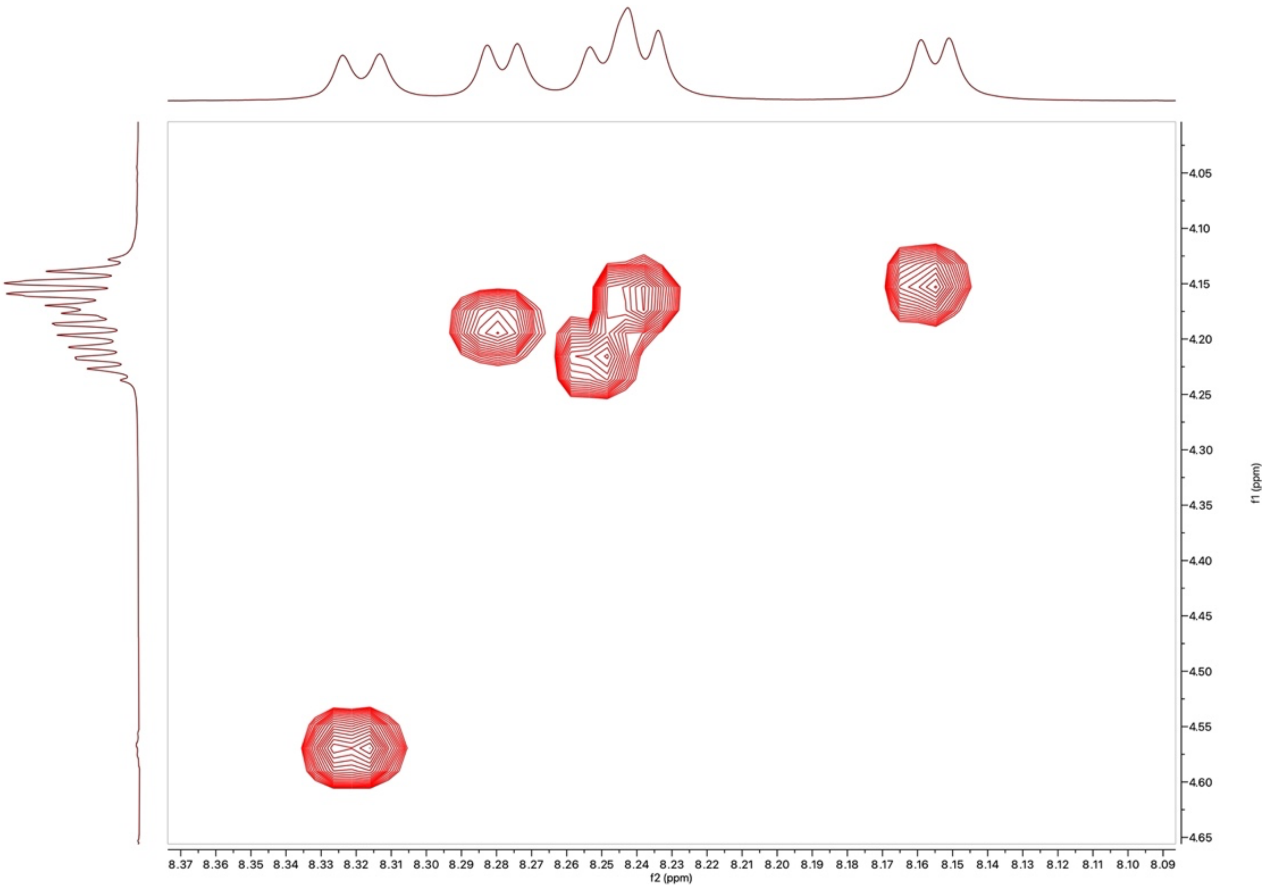

Figure S11: TOCSY of 5 mM Ac-AAHAA-NH_2 (**Ac-2-NH$_2$**) in 90% H_2O:10%D_2O, 50 mM sodium acetate, pH 5.2 (700 MHz).

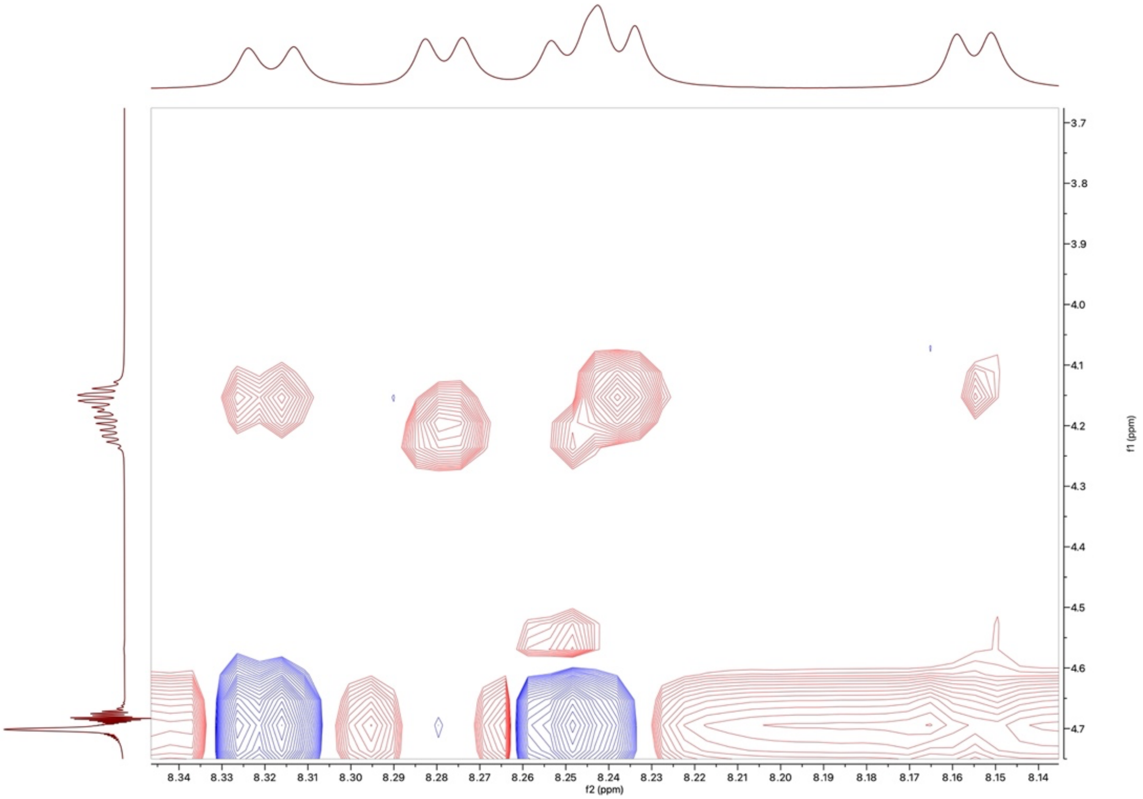

Figure S12: ROESY of 5 mM Ac-AAHAA-NH_2 (**Ac-2-NH$_2$**) in 90% H_2O:10%D_2O, 50 mM sodium acetate, pH 5.2 (700 MHz).

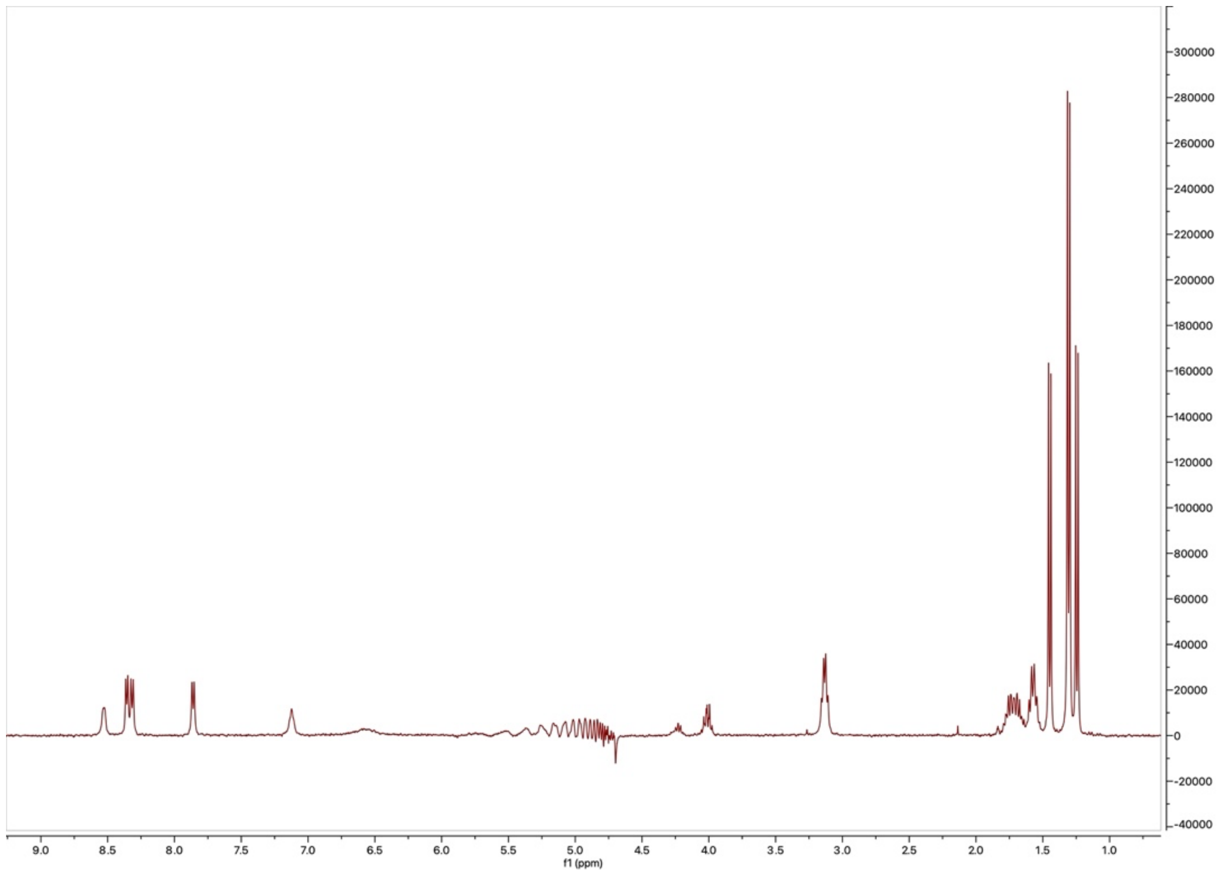

Figure S13: ^1H proton of 5 mM AARAA (**3**) in 90% H_2O:10%D_2O, 50 mM sodium acetate, pH 5.2 (700 MHz).

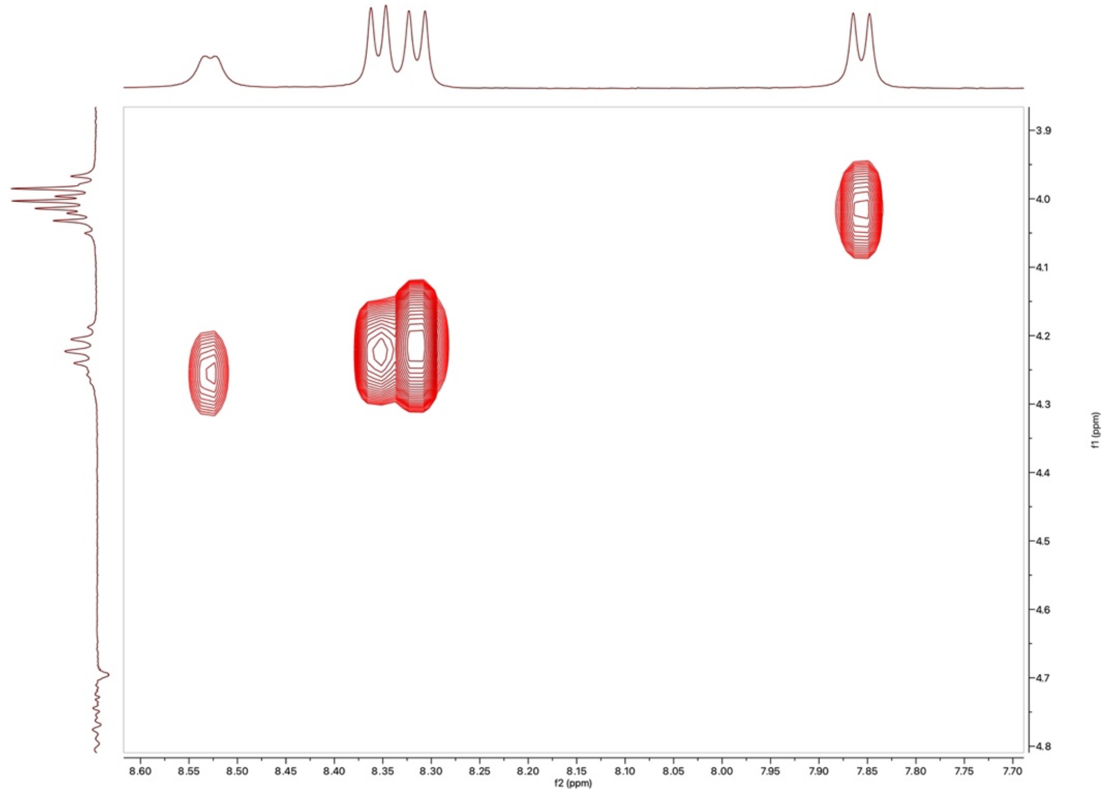

Figure S14: TOCSY of 5 mM AARAA (**3**) in 90% H_2O:10%D_2O, 50 mM sodium acetate, pH 5.2 (700 MHz).

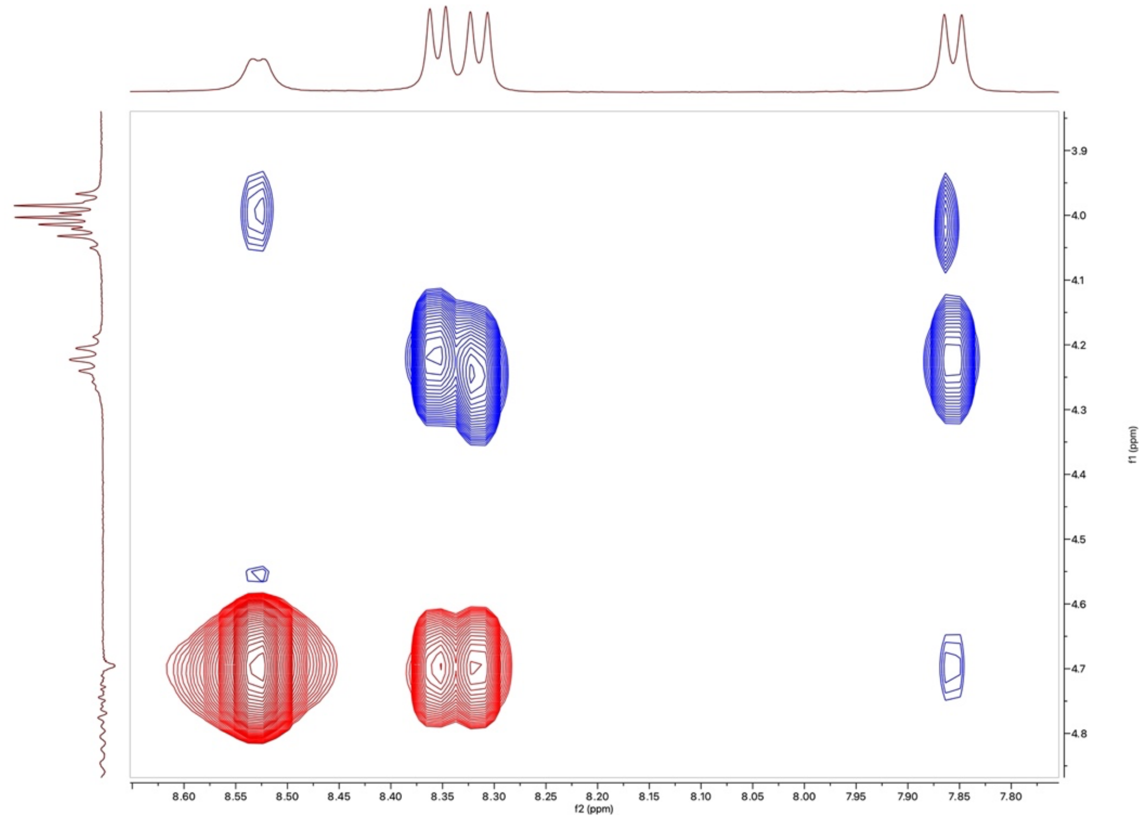

Figure S15: ROESY of 5 mM AARAA (**3**) in 90% H_2O:10%D_2O, 50 mM sodium acetate, pH 5.2 (700 MHz).

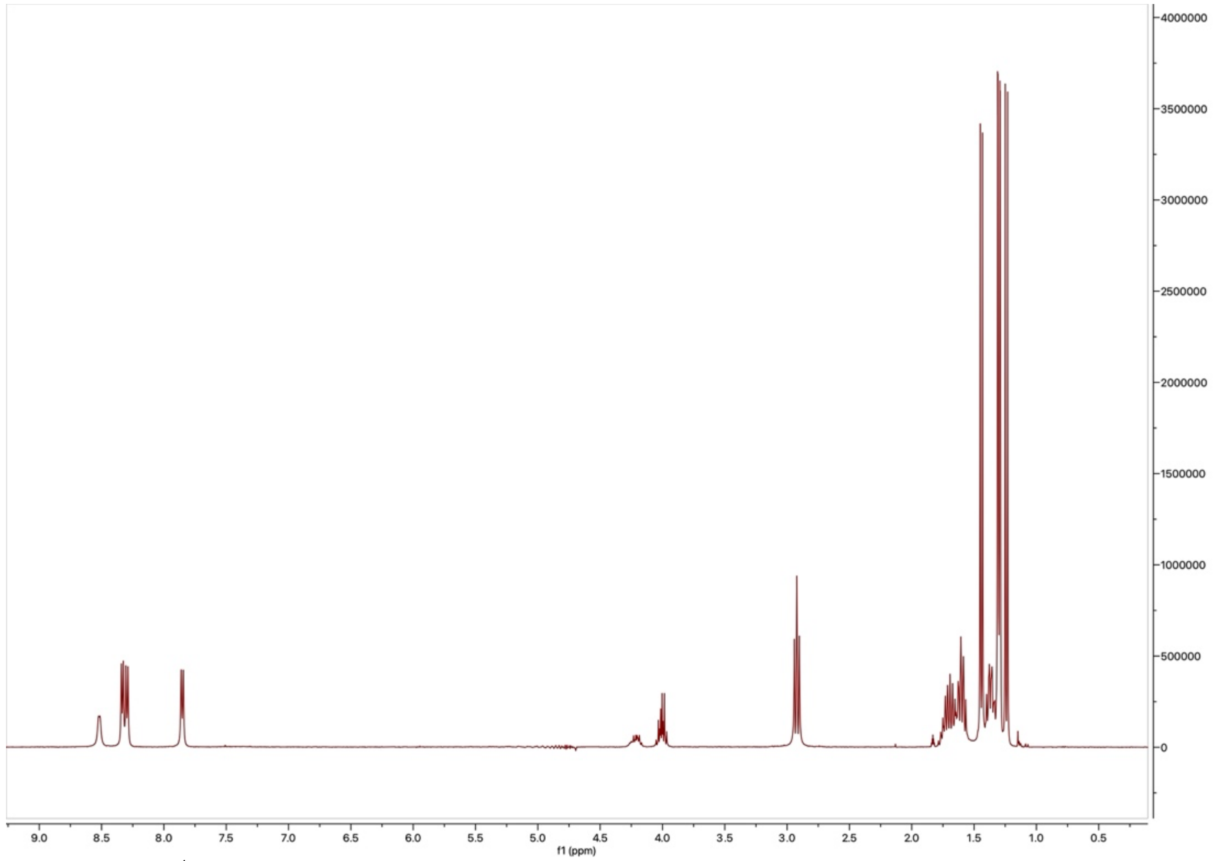

Figure S16: ^1H proton of 5 mM AAKAA (**4**) in 90% H_2O:10%D_2O, 50 mM sodium acetate, pH 5.2 (700 MHz).

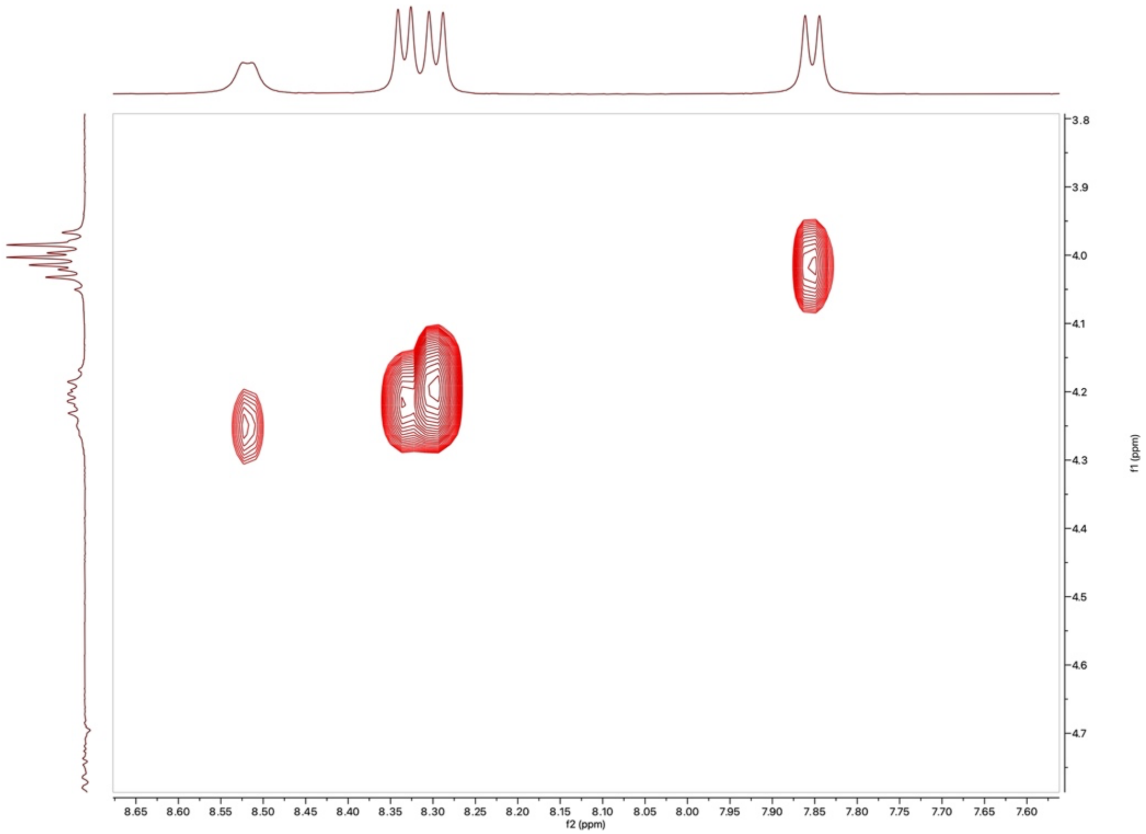

Figure S17: TOCSY of 5 mM AAKAA (**4**) in 90% H_2O:10%D_2O, 50 mM sodium acetate, pH 5.2 (700 MHz).

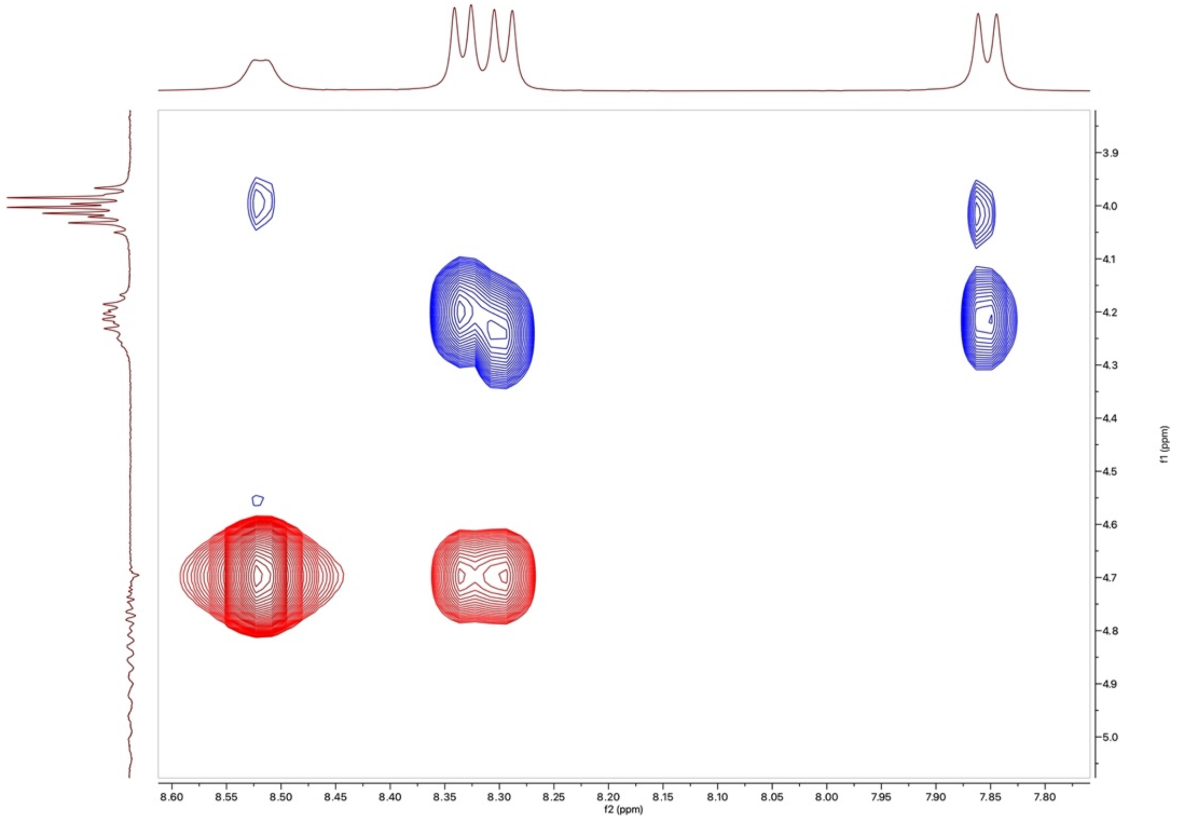

Figure S18: ROESY of 5 mM AAKAA (**4**) in 90% H_2O:10%D_2O, 50 mM sodium acetate, pH 5.2 (700 MHz).

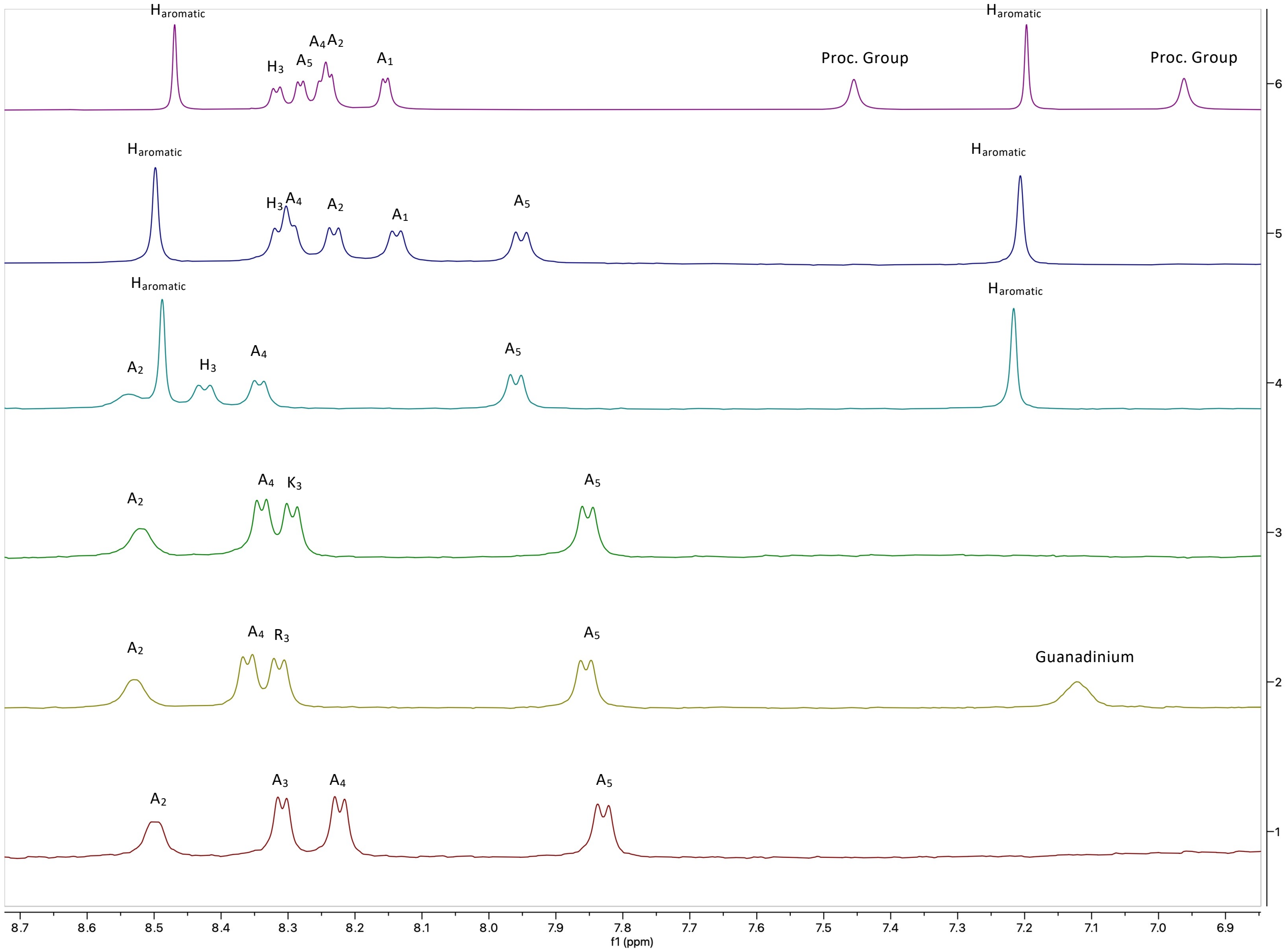

Figure S19: Peak assignment of the N-H region of the ^{1}H NMR spectra of each peptide. Spectra are: 1: AAAAA (**1**), 2: AARAA (**3**). 3: AAKAA (**4**). 4: AAHAA (**2**). 5: Ac-AAHAA (Ac-**2**). 6: Ac-AAHAA-NH$_2$ (Ac-**2**-NH$_2$). All solutions are 1 mM peptide in 10 mM acetate pH 5.2 in 90% H_2O:10% D_2O.

3) 2D NMR Titration Data for Peptide 2 with Perchlorate

Titrations involving data-points derived from 2D NMR experiments (2D NMR titrations) are typically time-consuming experiments. However, to ensure obtaining optimal data, a 2D NMR titration was initially performed with peptide **2** and perchlorate. Aliquots of 2 M $NaClO_4$ were added up to 500 mM salt to a 5 mM AAHAA in 50 mM acetate buffer pH 5.2. A total of ten HSQC spectra were collected. Figure S20 shows first the 1D NMR titration data for **2**. Figure S21 shows the amide N–H region from the 2D ^{1}H-^{15}N HSQC titration experiment, showing signal shifts in both the proton and nitrogen dimensions.

a)

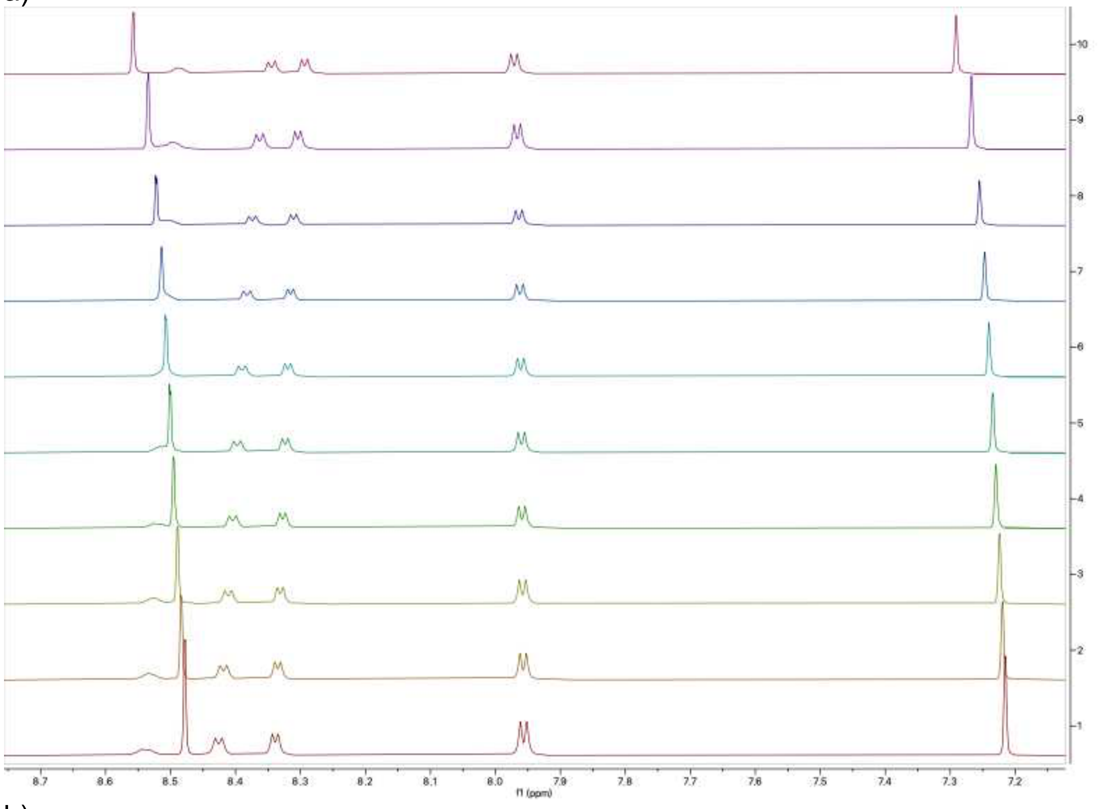

b)

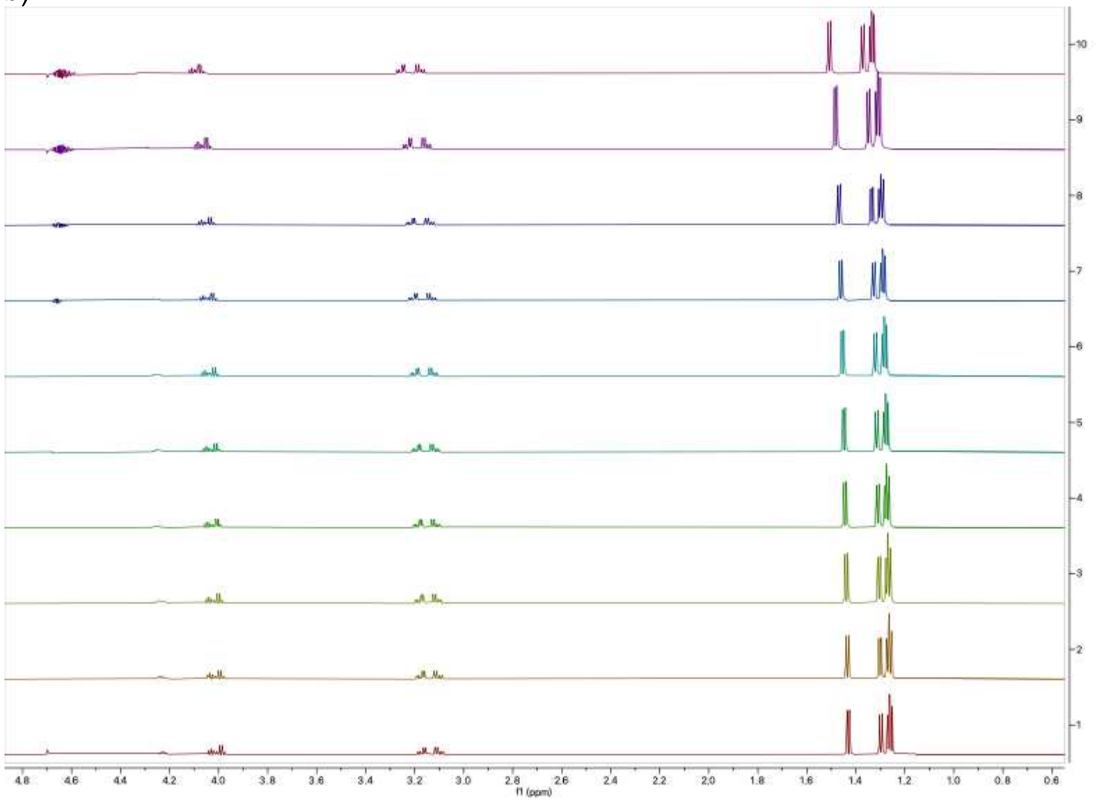

Figure S20: ^{1}H proton of 5 mM AAHAA (**2**) in 90% H_2O:10%D_2O, 50 mM sodium acetate, pH 5.2 (700 MHz) titrated with 2 M $NaClO_4$ from 0 (spectra-1) to 500 mM (spectra-10). a) Amide and aromatic proton region; b) side chain and C_α protons.

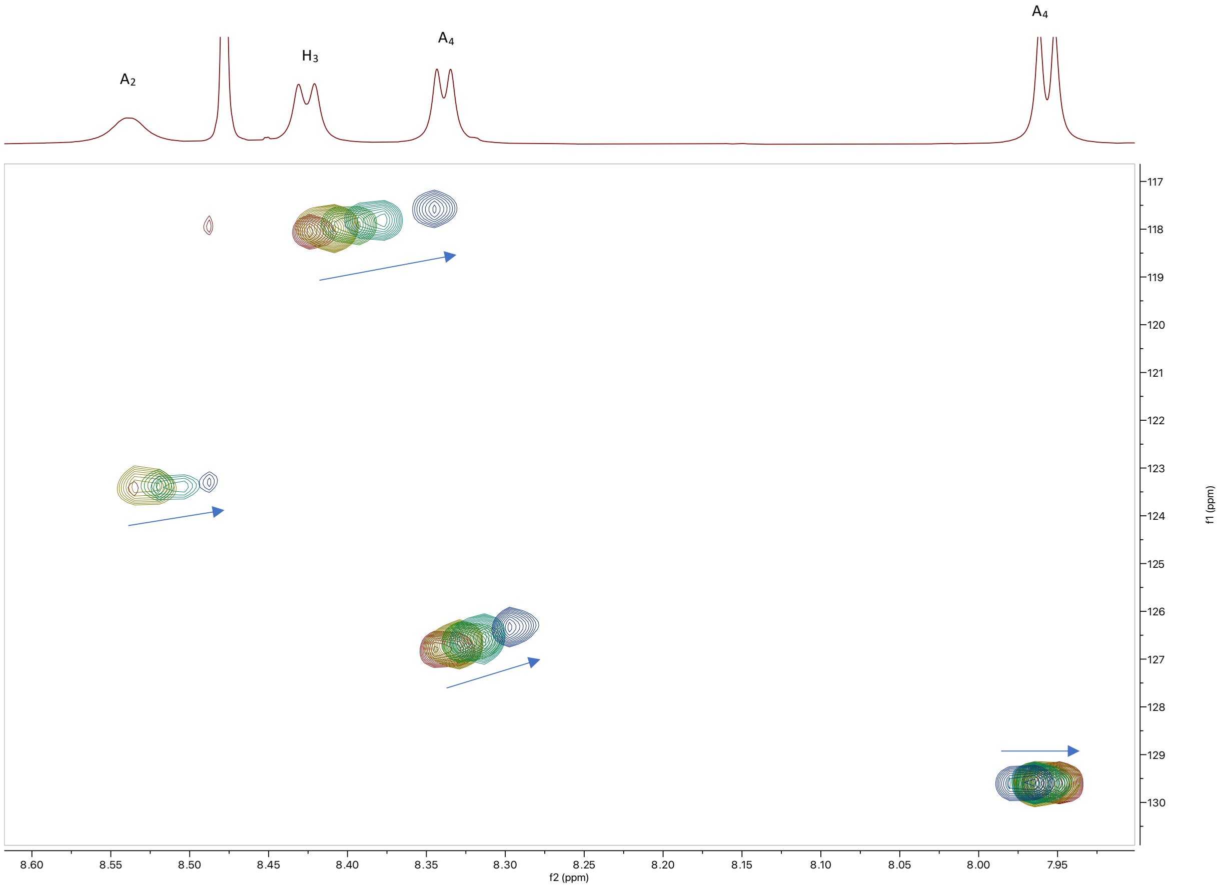

Figure S21: ^{1}H-^{15}N HSQC spectra of 5 mM AAHAA (**2**) titrated with 2M $NaClO_4$ from 0 to 500 mM. Solutions were 90% H_2O:10% D_2O, 50 mM sodium acetate, pH 5.2 (700 MHz). Identical solutions were used in Figure S20.

a) Analysis of 2D titration with peptide 2

For the ^{1}H-^{15}N HSQC titration experiments, the unreferenced shifts in both the proton and nitrogen dimensions were plotted (Figures S22 and S23). The proton dimension shifts show a clear picture of weak anion association to the amide N–H groups. Thus, whereas the C–H_α and CH_3 proton signals shift similarly downfield because of ionic strength changes, the N-H proton signals showed larger upfield shifts in the proton dimension. (NB. The N-H protons of the *N*-terminus exchanges too fast to be observed by NMR). In contrast, the Δδ of the N–H groups in the nitrogen dimension was much smaller (–0.3 ppm at 250 mM) and the data noisy (Figure S23). Figure S24 shows the combined data with the standard deviations shown. To minimize ionic strength effects, all subsequent titrations were carried up to 250 mM salt.

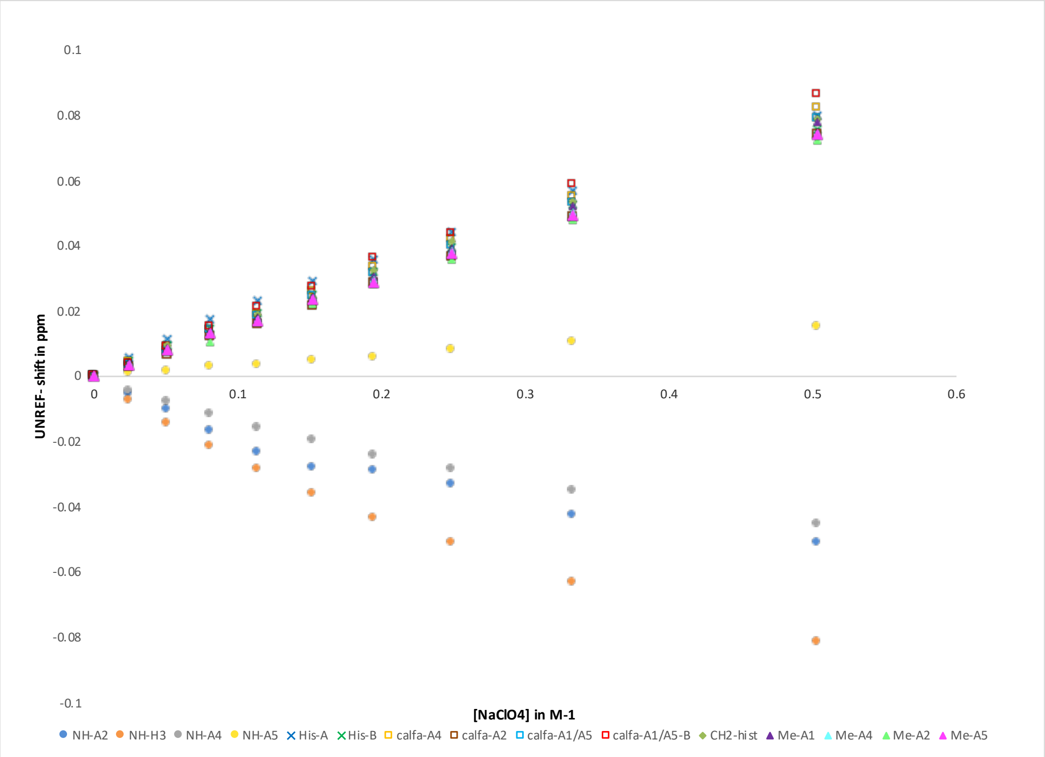

Figure S22: Unreferenced proton dimension signal shifts (ppm) of all protons for peptide **2** (see also Figures S20-S22) during titration with $NaClO_4$ up to 500 mM. Solution was 5 mM in 90% H_2O:10% D_2O, 50 mM sodium acetate, pH 5.2 (700 MHz).

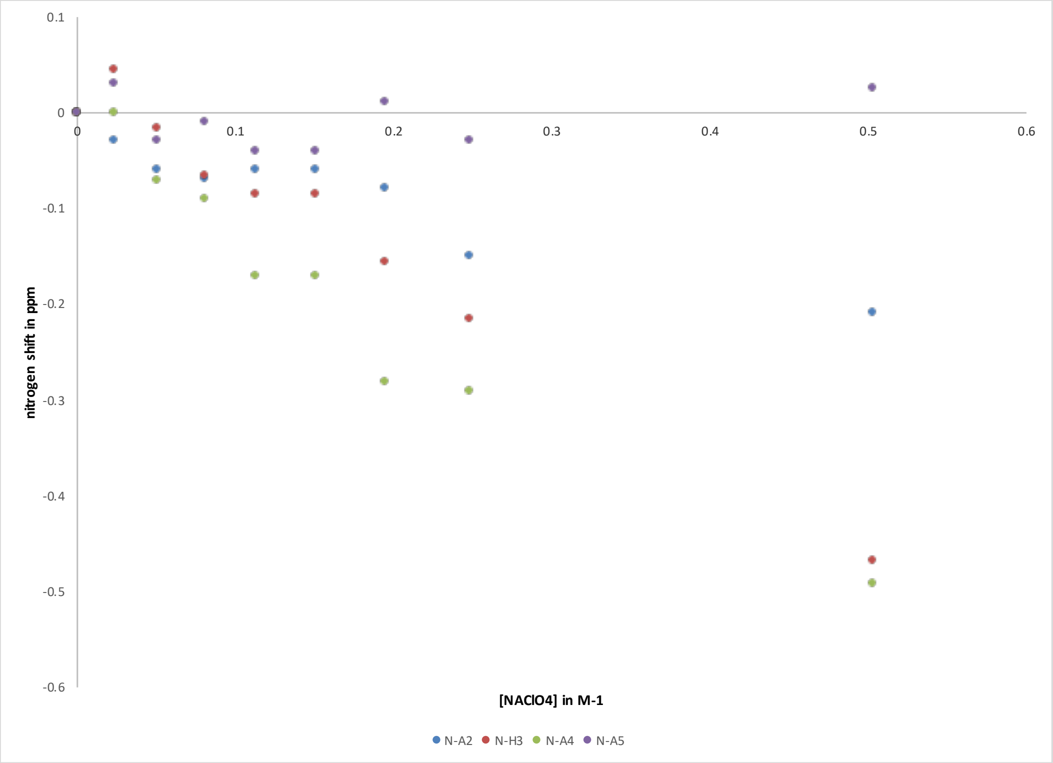

Figure S23: Unreferenced nitrogen dimension signal shifts (ppm) of all amide groups of peptide AAHAA during titration with $NaClO_4$ up to 500 mM. Solution was 5 mM in 90% H_2O:10% D_2O, 50 mM sodium acetate, pH 5.2 (700 MHz).

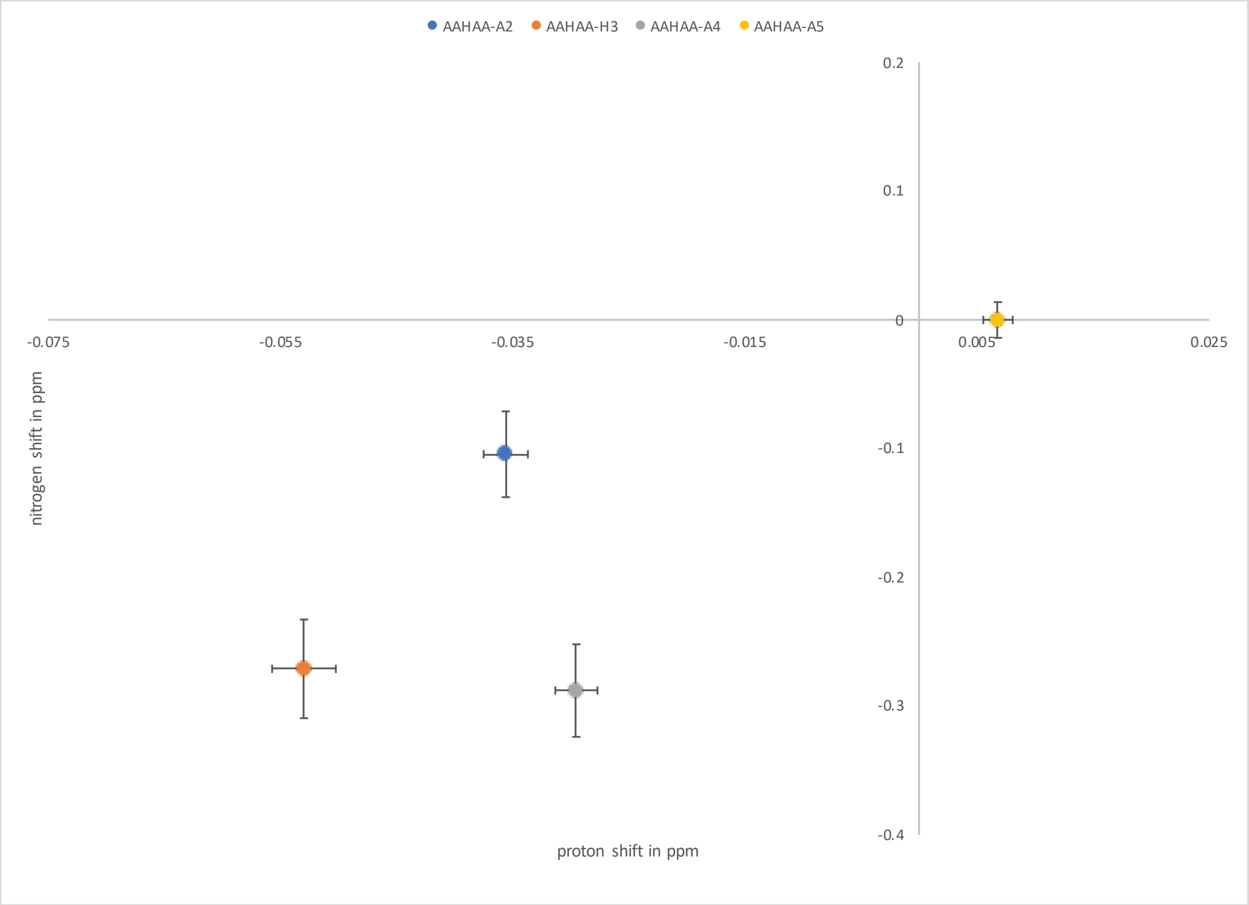

Figure S24: Unreferenced 2D shifts (ppm) for N–H groups of peptide **2** from ^{1}H-^{15}N HSQC spectra at 0 and 250 mM $NaClO_4$. Solution was 90% H_2O:10% D_2O, 50 mM sodium acetate, pH 5.2. Errors (standard deviations) represent the average of at least three titrations using fresh solutions.

b) Referencing the NMR data

Control experiments showed that at 250 mM of salt the NMR signal shifts caused by ionic strength changes were small. Thus, during titration, the signal shift for both an added standard (sodium 3-(trimethylsilyl)propane-1-sulfonate, DSS) and the *C*-terminus, 5-Ala methyl were only ~0.001 ppm. Consequently, the DSS standard was omitted, and all data referenced to the *C*-terminus methyl. As shown in Figure S25, all protons other than N-H protons underwent similar, small shifts.

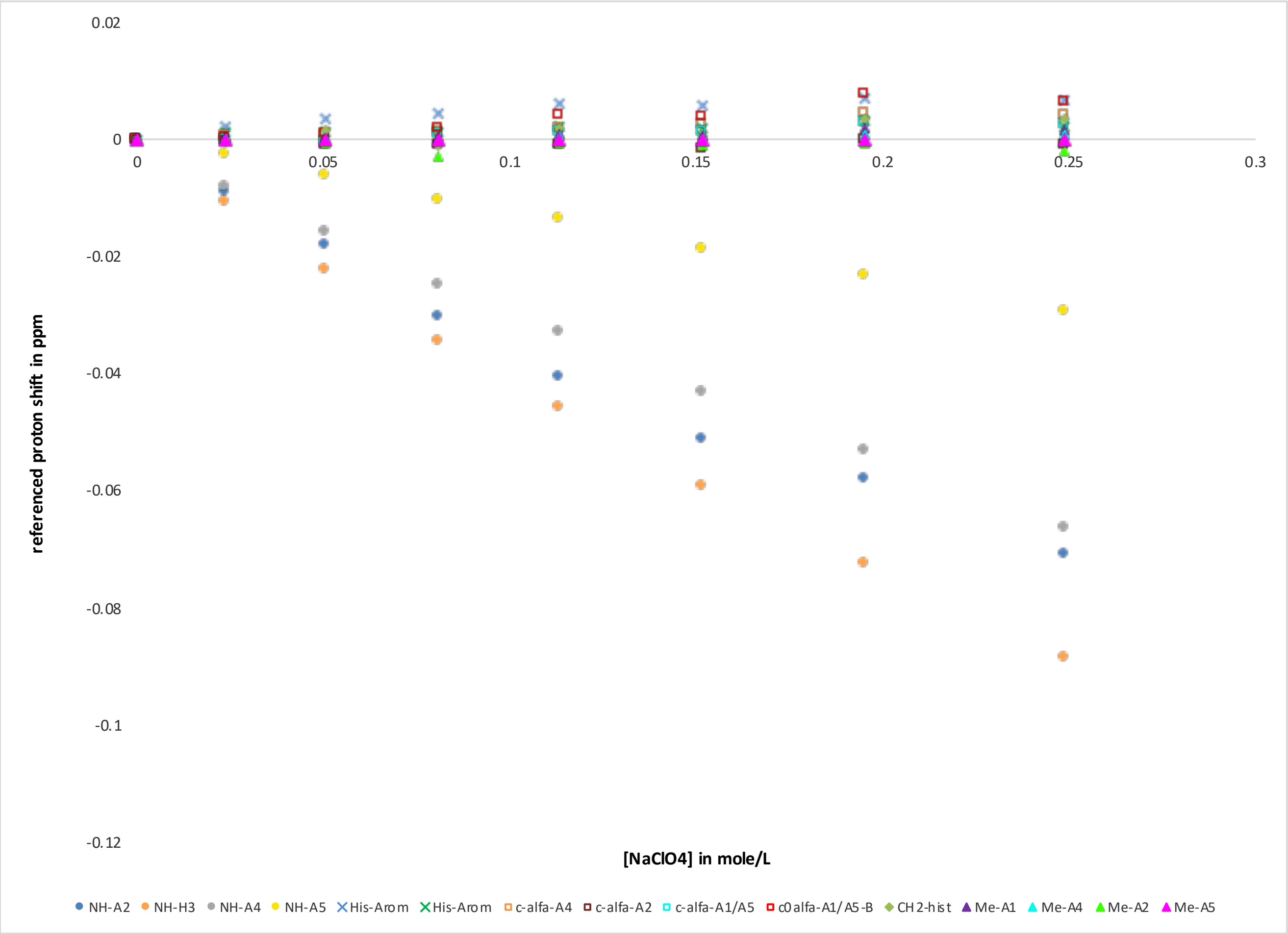

Figure S25: Referenced proton dimension signal shifts (ppm) of all protons of peptide **2**. Solution was 90% H_2O:10%D_2O, 50 mM sodium acetate, pH 5.2 (700 MHz) titrated with $NaClO_4$ to 250 mM. For comparison, the unreferenced data is shown in Figure S22.

4) Single point 2D titrations 1, Ac-**2**, Ac-**2**-NH_2, **3** and **4.**

Building on the full 2D NMR titration of peptide **2** with perchlorate, single point 2D titrations, whereby only no salt and 250 mM salt conditions were studied, were carried out for the other peptides under investigation. This allowed rapid qualification of anion binding. Shown below (Figures S26-S30) are the unreferenced 2D shifts between 0 and 250 mM $NaClO_4$ for peptides **1**, **Ac-2**, **Ac-2-NH$_2$**, **3** and **4**. Figure S31 collates the data for all six peptides.

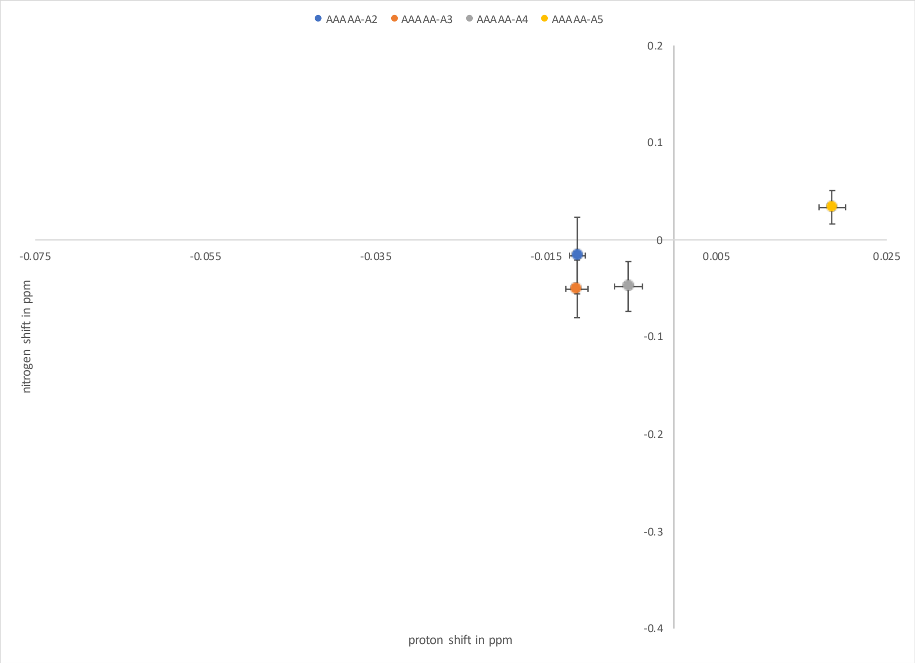

Figure S26: Unreferenced 2D shifts (ppm) for N–H groups of peptide **1** from ^{1}H-^{15}N HSQC spectra at 0 and 250 mM $NaClO_4$. All solutions were 90% H_2O:10% D_2O, 50 mM sodium acetate, pH 5.2. Errors (standard deviations) represent the average of at least three titrations using fresh solutions.

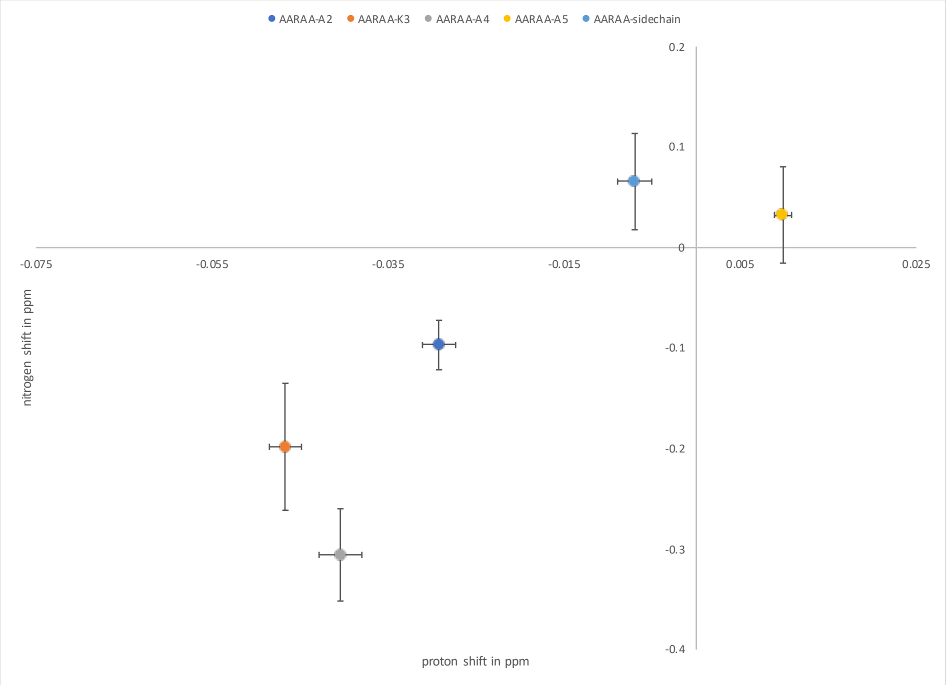

Figure S27: Unreferenced 2D shifts (ppm) for N–H groups of peptide **Ac-2** from ^{1}H-^{15}N HSQC spectra at 0 and 250 mM $NaClO_4$. All solutions were 90% H_2O:10% D_2O, 50 mM sodium acetate, pH 5.2. Errors (standard deviations) represent the average of at least three titrations using fresh solutions.

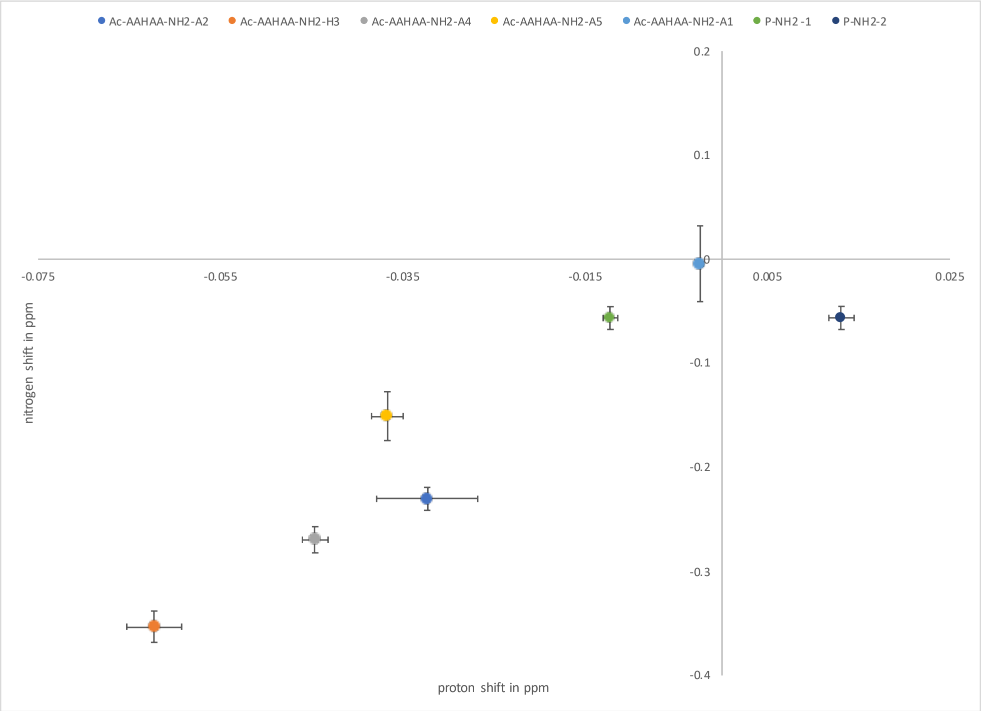

Figure S28: Unreferenced 2D shifts (ppm) for N–H groups of peptide **Ac-2-NH$_2$** from ^1H-^{15}N HSQC spectra at 0 and 250 mM $NaClO_4$. All solutions were 90% H_2O:10% D_2O, 50 mM sodium acetate, pH 5.2. Errors (standard deviations) represent the average of at least three titrations using fresh solutions.

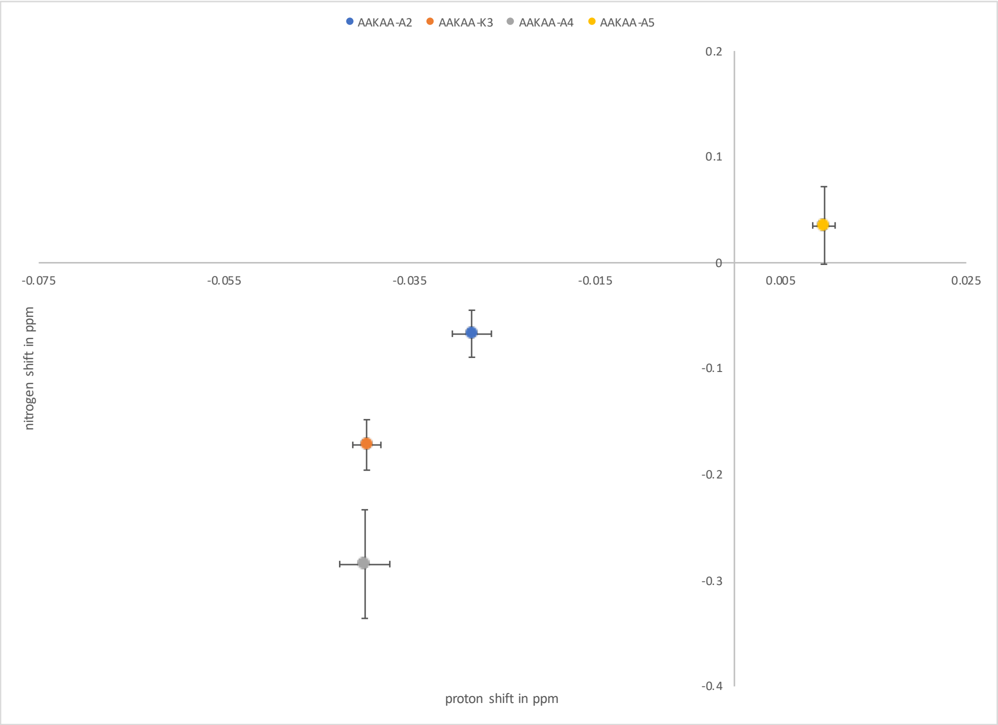

Figure S29: Unreferenced 2D shifts (ppm) for N–H groups of peptide **3** from ^1H-^{15}N HSQC spectra at 0 and 250 mM $NaClO_4$. All solutions were 90% H_2O:10% D_2O, 50 mM sodium acetate, pH 5.2. Errors (standard deviations) represent the average of at least three titrations using fresh solutions.

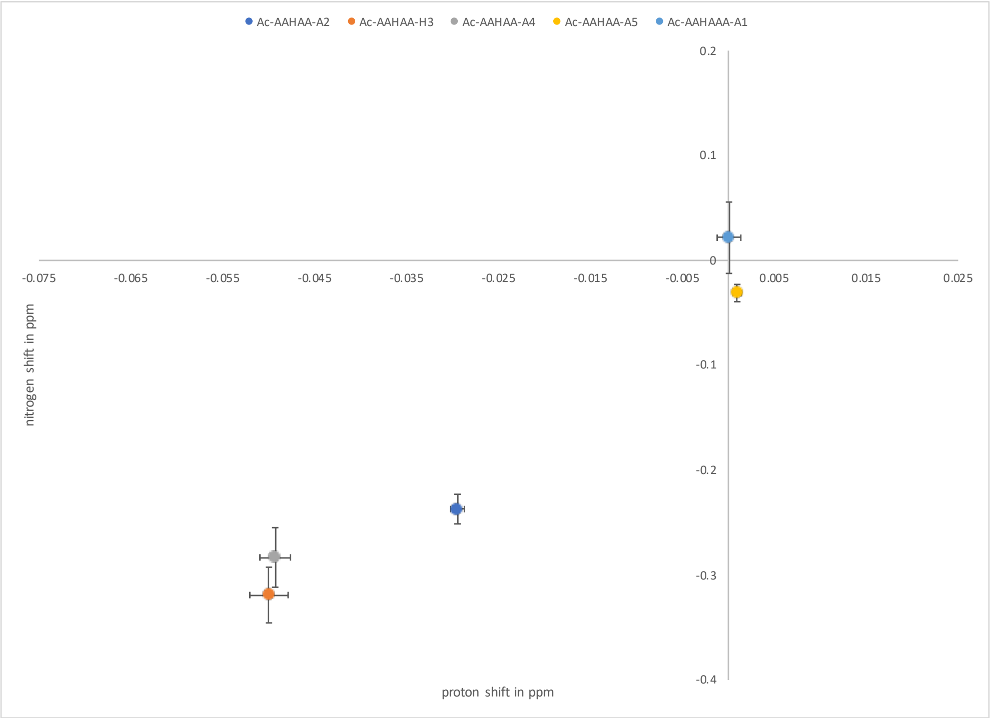

Figure S30: Unreferenced 2D shifts (ppm) for N–H groups of peptide **4** from ^{1}H-^{15}N HSQC spectra at 0 and 250 mM $NaClO_4$. All solutions were 90% H_2O:10% D_2O, 50 mM sodium acetate, pH 5.2. Errors (standard deviations) represent the average of at least three titrations using fresh solutions.

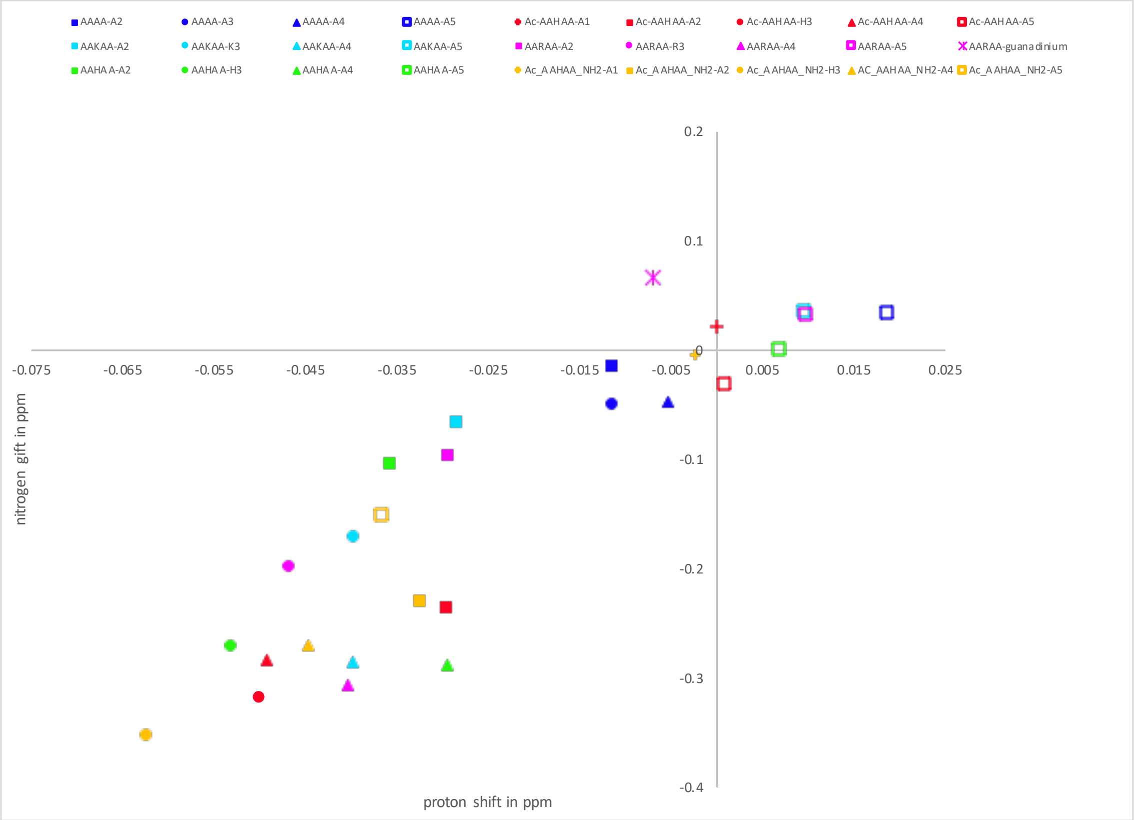

Figure S31: Unreferenced 2D shifts (ppm) for N–H groups of all peptides derived from ^{1}H-^{15}N HSQC spectra at 0 and 250 mM $NaClO_4$. All solutions were 90% H_2O:10% D_2O, 50 mM sodium acetate, pH 5.2.

5) 1D Titrations of all Peptides with Perchlorate

To compare the data obtained from single point 2D titrations with full titrations curves, all peptides were subjected to 1D titration experiments with $NaClO_4$ up to 250 mM salt concentration. These titrations allowed the concentration of the peptide to be lowered from 5 mM to 1mM and allowed the use of the 400 MHz instrument in the majority of cases (where peak overlap was not an issue). This greatly reduced sample requirements and data acquisition time. In a few cases it was necessary to use the 700 MHz instrument to provide better resolution of peaks and follow their movement during the titration. In the case of **2**, it was not possible to monitor all peaks at all salt concentrations, but here the control 2D titrations confirmed that the 1D titration had sufficient data even in the absence of all data points. All 1-D titrations were referenced to the *C*-terminus methyl alanine proton during analysis, and were performed in 90% H_2O:10% D_2O, 1 mM peptide in 10 mM sodium acetate buffer at pH 5.2. Figures S32-S37 show the amide N–H region (and in select cases the full spectrum) of each peptide during the $NaClO_4$ titration.

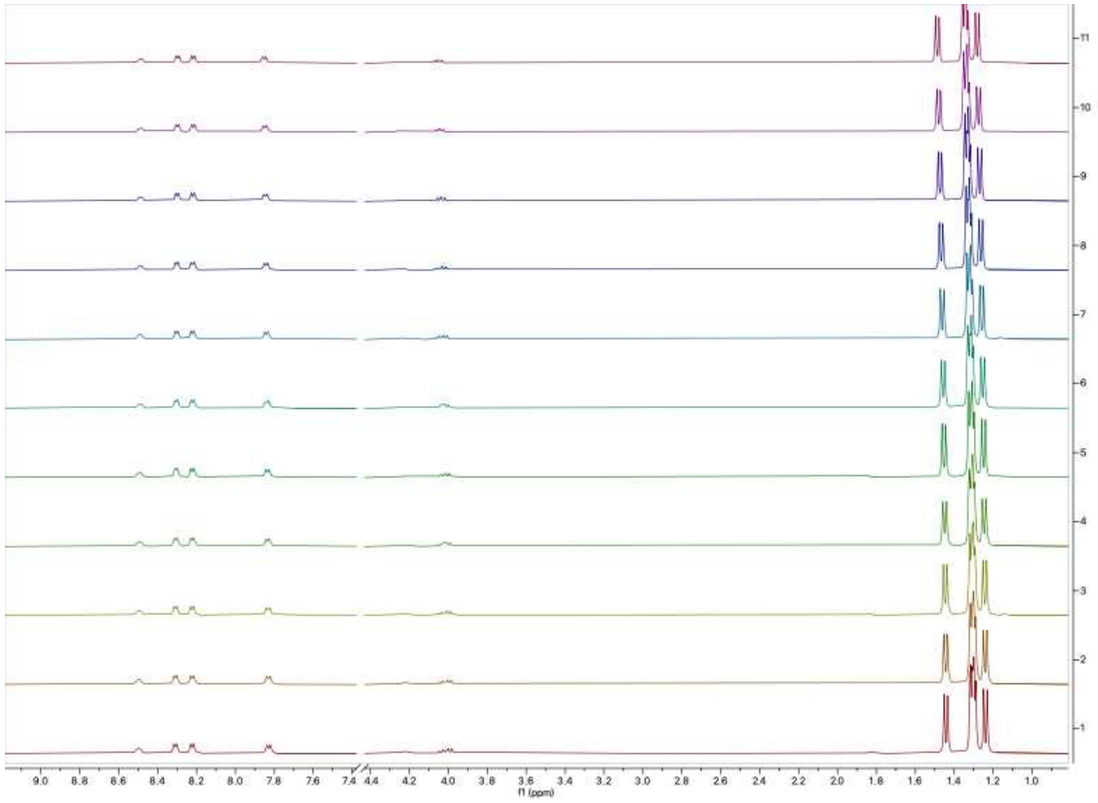

a)

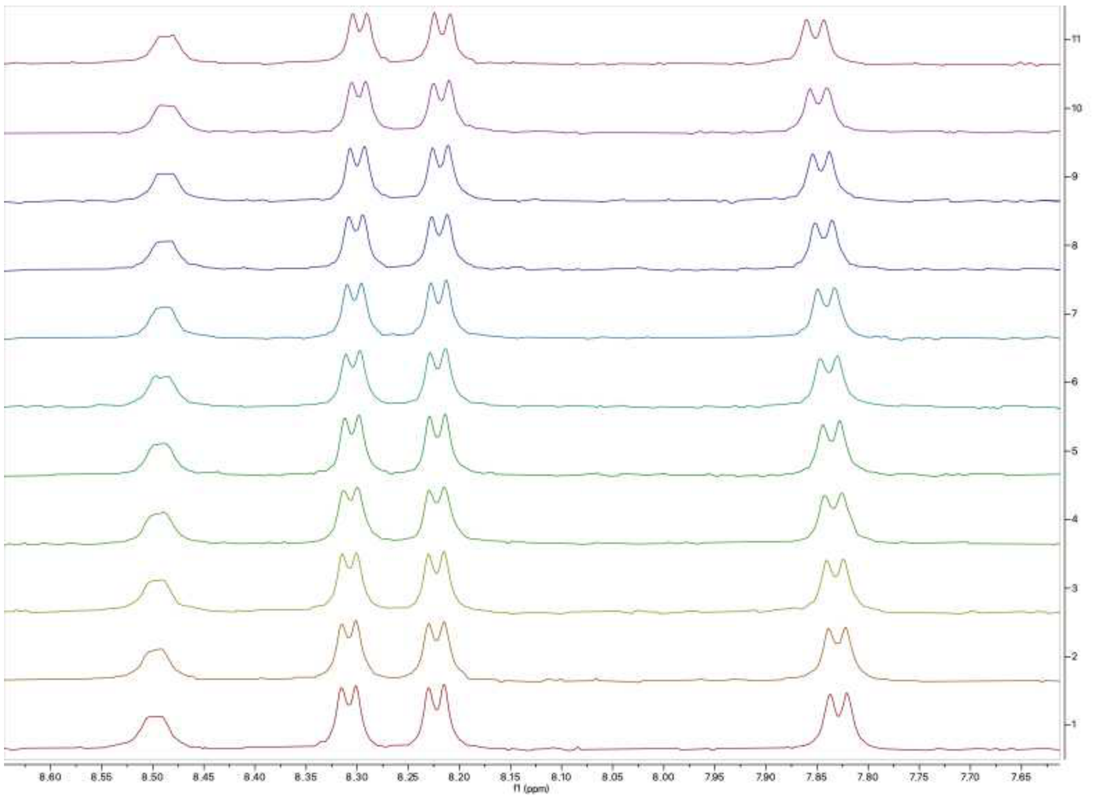

b)

Figure S32: $NaClO_4$ titration of 1 mM **1** in 10 mM acetate buffer, pH 5.2 from 0 (NMR-1) to 250 mM (NMR-11) salt (400 MHz). a) Full spectra; b) amide N-H region.

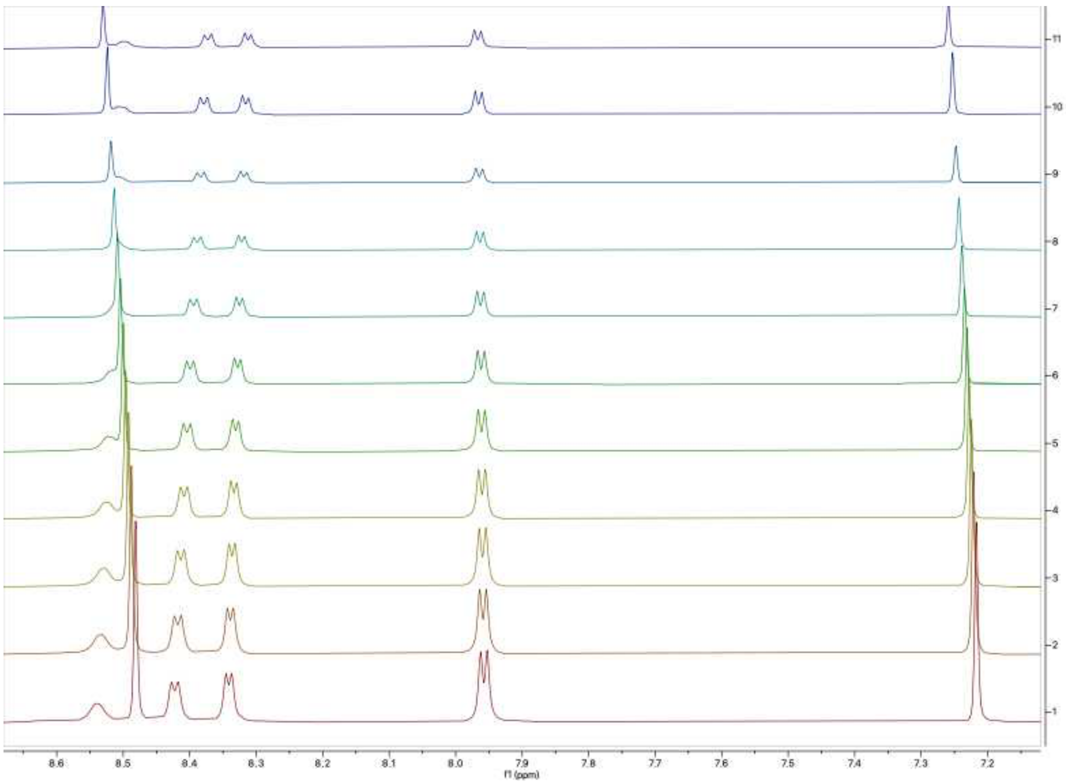

Figure S33: Amide N-H region of ^{1}H NMR spectrum of peptide **2**. The stack shows data from a $NaClO_4$ titration from 0 (NMR-1) to 250 mM (NMR-11) salt (400 MHz). All solutions, 1 mM **2** in 90% H_2O:10% D_2O, 10 mM acetate buffer, pH 5.2.

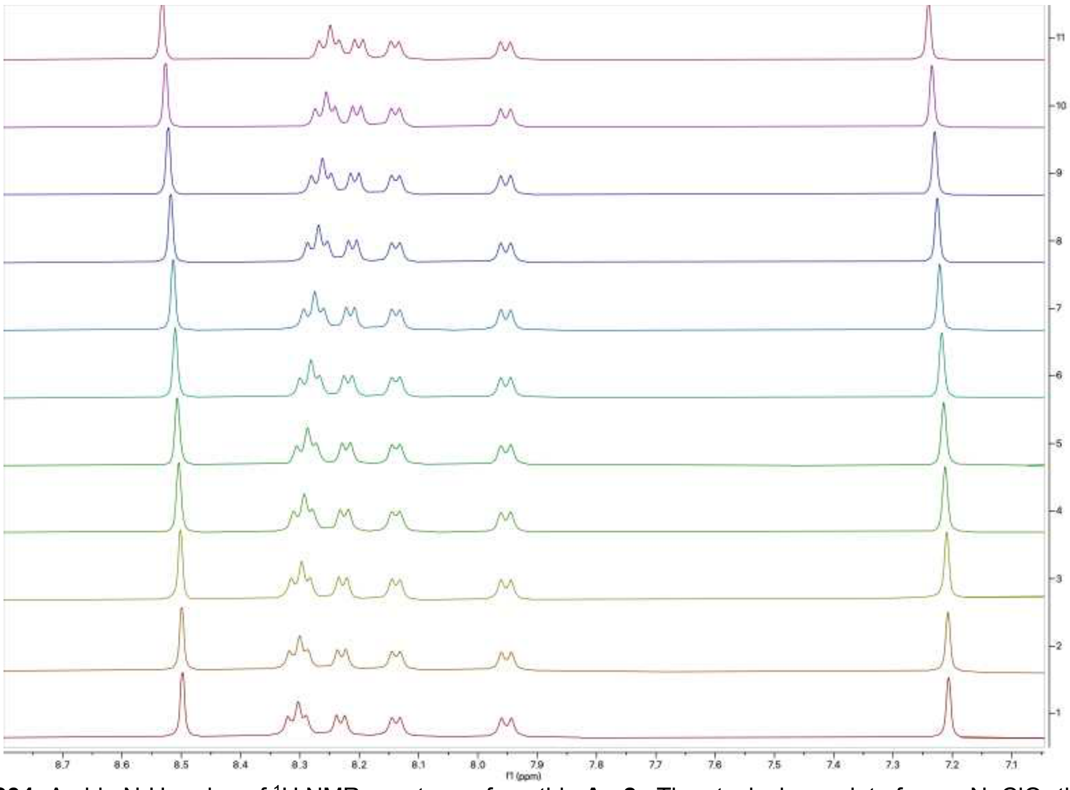

Figure S34: Amide N-H region of ^{1}H NMR spectrum of peptide **Ac-2**. The stack shows data from a $NaClO_4$ titration from 0 (NMR-1) to 250 mM (NMR-11) salt (400 MHz). All solutions, 1 mM **Ac-2** in 90% H_2O:10% D_2O, 10 mM acetate buffer, pH 5.2.

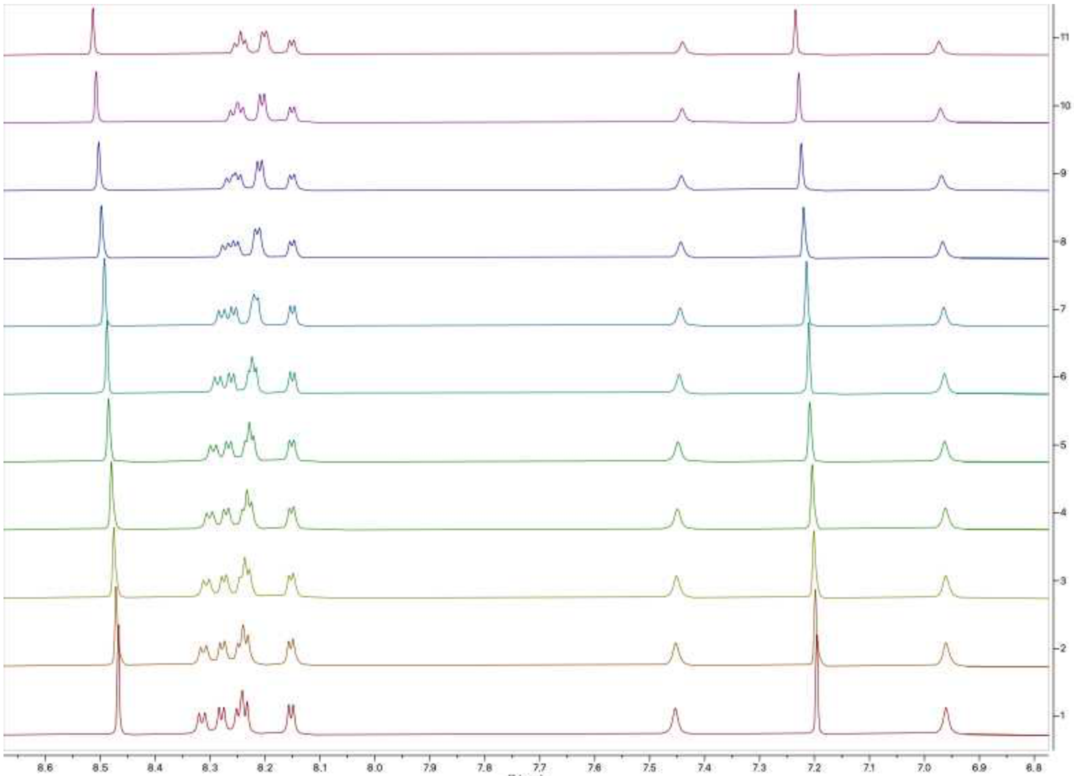

a)

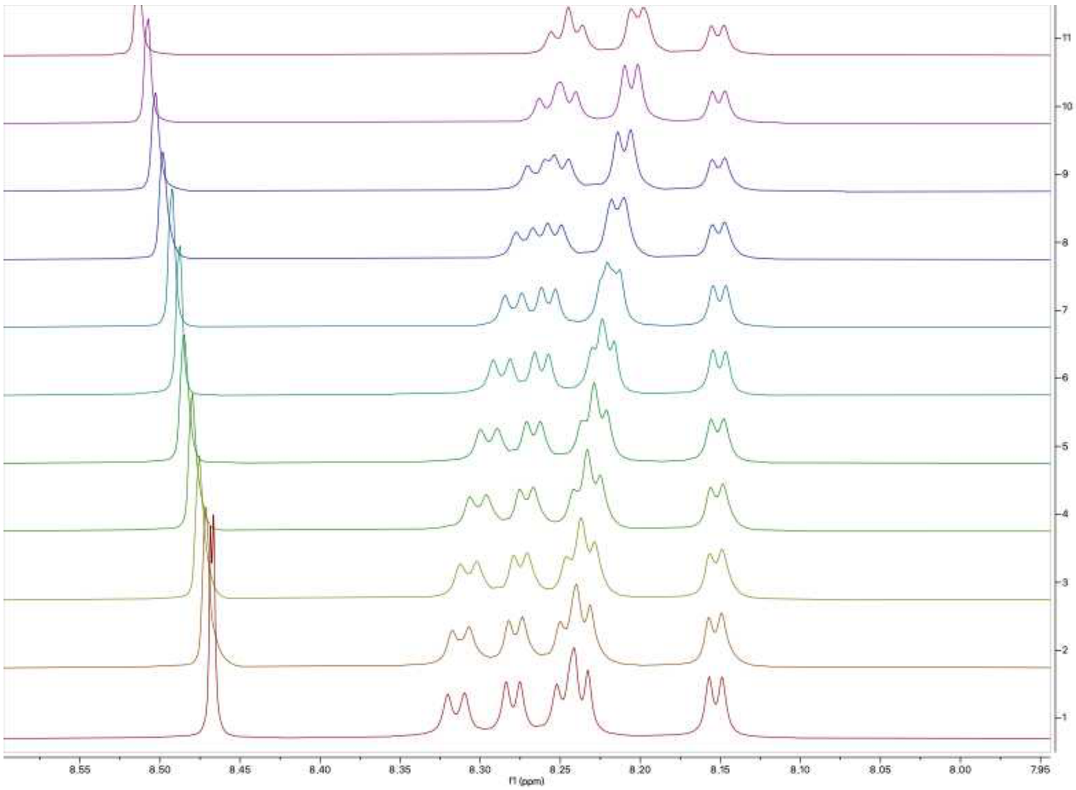

b)

Figure S35: $NaClO_4$ titration of 1 mM **Ac-2-NH$_2$** in 10 mM acetate buffer, pH 5.2 from 0 (NMR-1) to 250 mM (NMR-11) salt (400 MHz). a) Full spectra; b) amide N-H region.

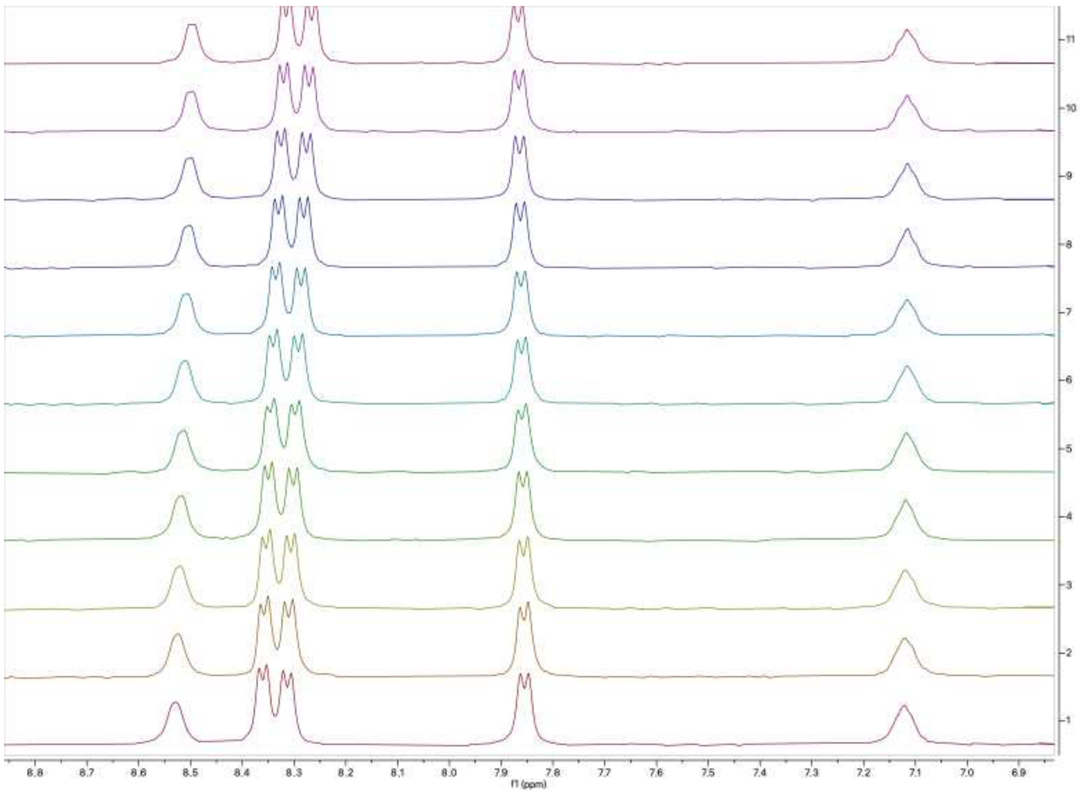

Figure S36: Amide N-H region of ^{1}H NMR spectrum of peptide **3**. The stack shows data from a $NaClO_4$ titration from 0 (NMR-1) to 250 mM (NMR-11) salt (400 MHz). All solutions, 1 mM **3** in 90% H_2O:10% D_2O, 10 mM acetate buffer, pH 5.2.

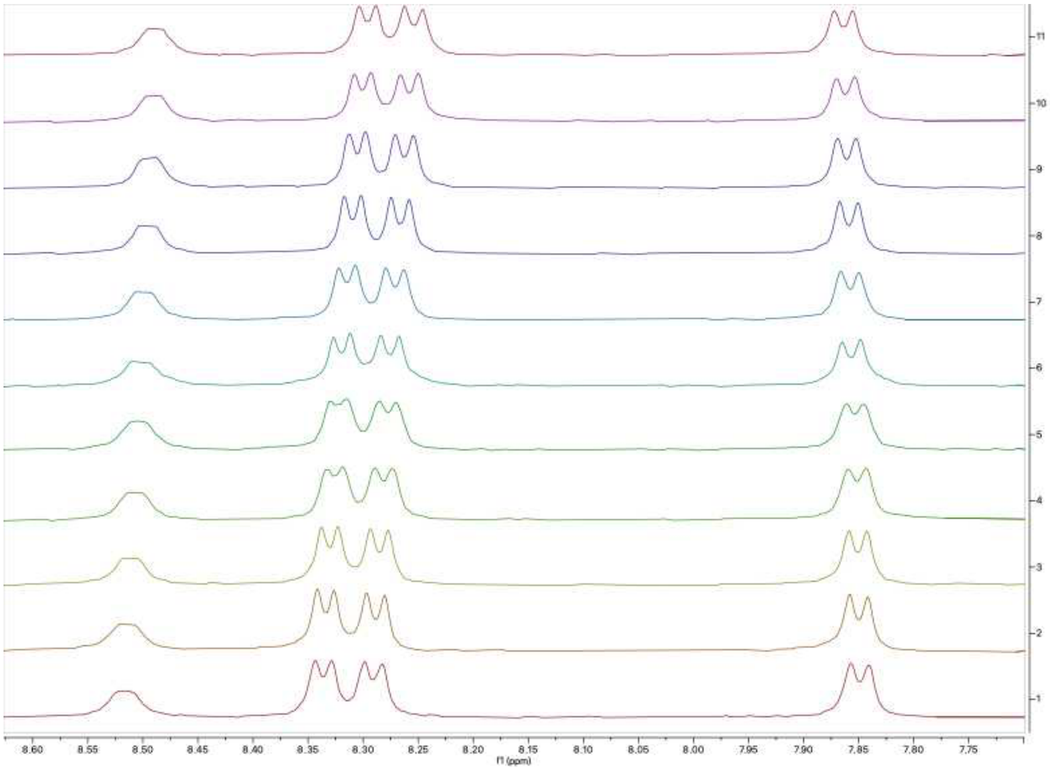

Figure S37: Amide N-H region of ^{1}H NMR spectrum of peptide **4**. The stack shows data from a $NaClO_4$ titration from 0 (NMR-1) to 250 mM (NMR-11) salt (400 MHz). All solutions, 1 mM **4** in 90% H_2O:10% D_2O, 10 mM acetate buffer, pH 5.2.

6) Fitting of 1D Titration Data

All titrations (Figures S32-S37) were plotted as the N-H proton shifts vs concentration of salt added. All data shown is referenced to the methyl alanine of the *C*-terminus shifts at each titration points, Eq. 2:

$$\Delta\delta_{(}(X) = \delta_{(X\ \mathrm{mM})} - \delta_{(0\ \mathrm{mM})} - (\delta_{\mathrm{Me\text{-}5A}(X\ \mathrm{mM})} - \delta_{\mathrm{Me\text{-}5A}(0\ \mathrm{mM})}) \qquad \text{Eq. S2}$$

The data was then fitted to a 1:1 binding isotherm.[1, 2] Figure S38-S44 show, for each peptide, data-points for all observable proton and the corresponding fitting line. Each titration was repeated a minimum of three times. The data points at 250 mM from these titrations were used for single point titration analyses.

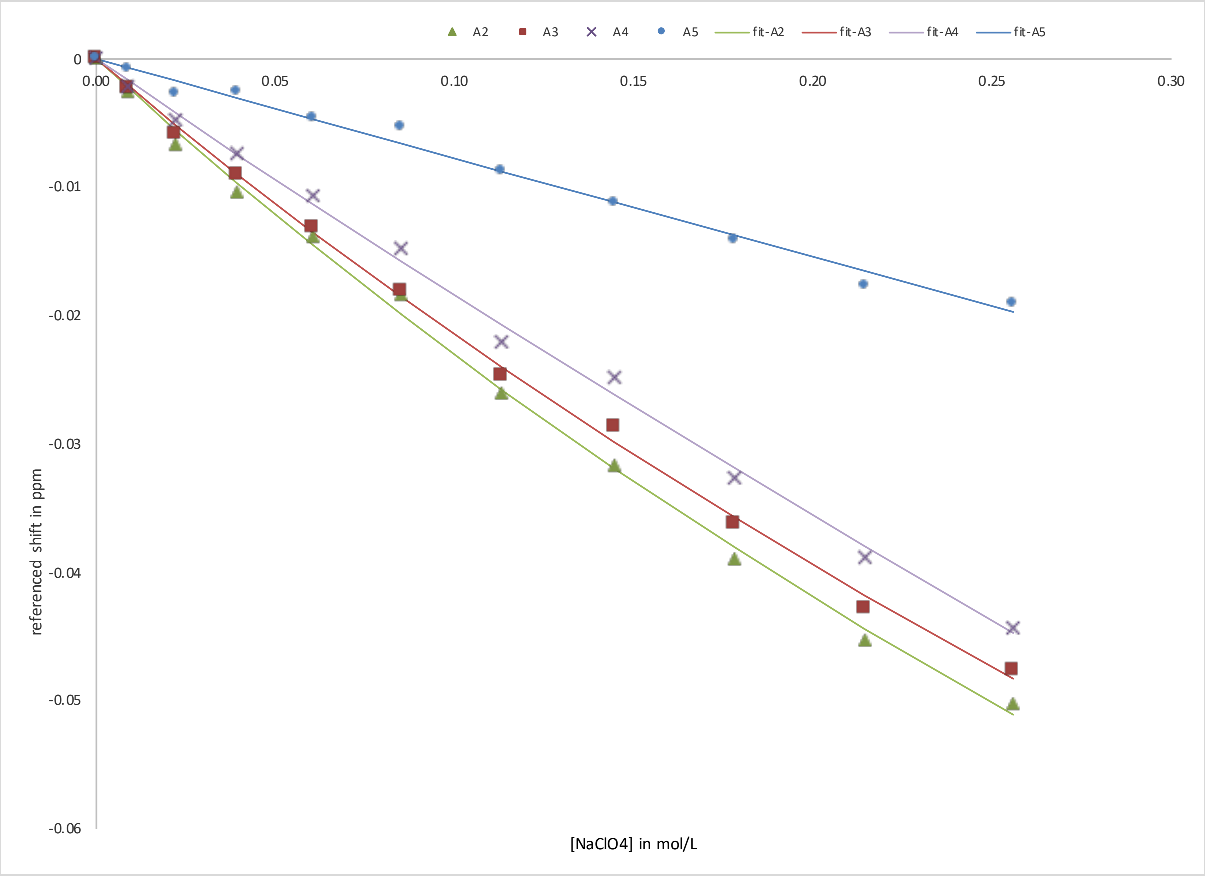

Figure S38: Fitted data from one of the titration **1** with $NaClO_4$ (0-250 mM salt). Data from Figure S32, and referenced to the 5A methyl signal. Solution was 1 mM **1** in 90% H_2O:10% D_2O, 10 mM acetate buffer, pH 5.2. Data was collected at 400 MHz.

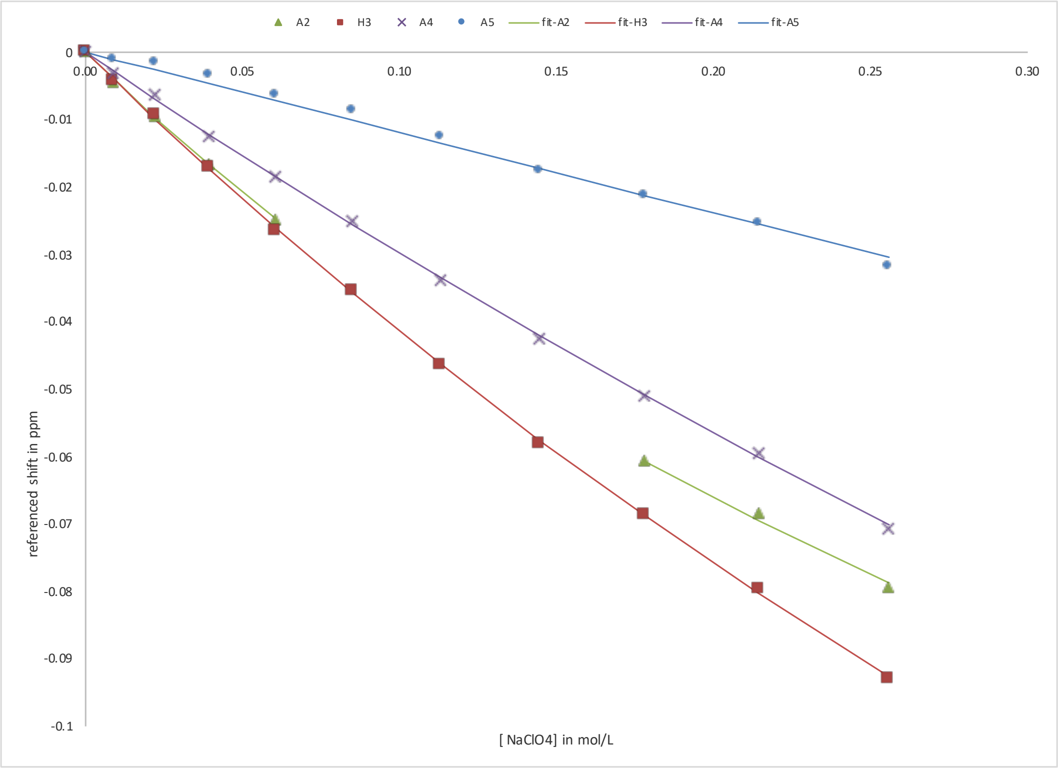

Figure S39: Fitted data from one of the titration **2** with $NaClO_4$ (0-250 mM salt). Data from Figure S33, and referenced to the 5A methyl signal. Solution was 1 mM **2** in 90% H_2O:10% D_2O, 10 mM acetate buffer, pH 5.2. Data was collected at 400 MHz.

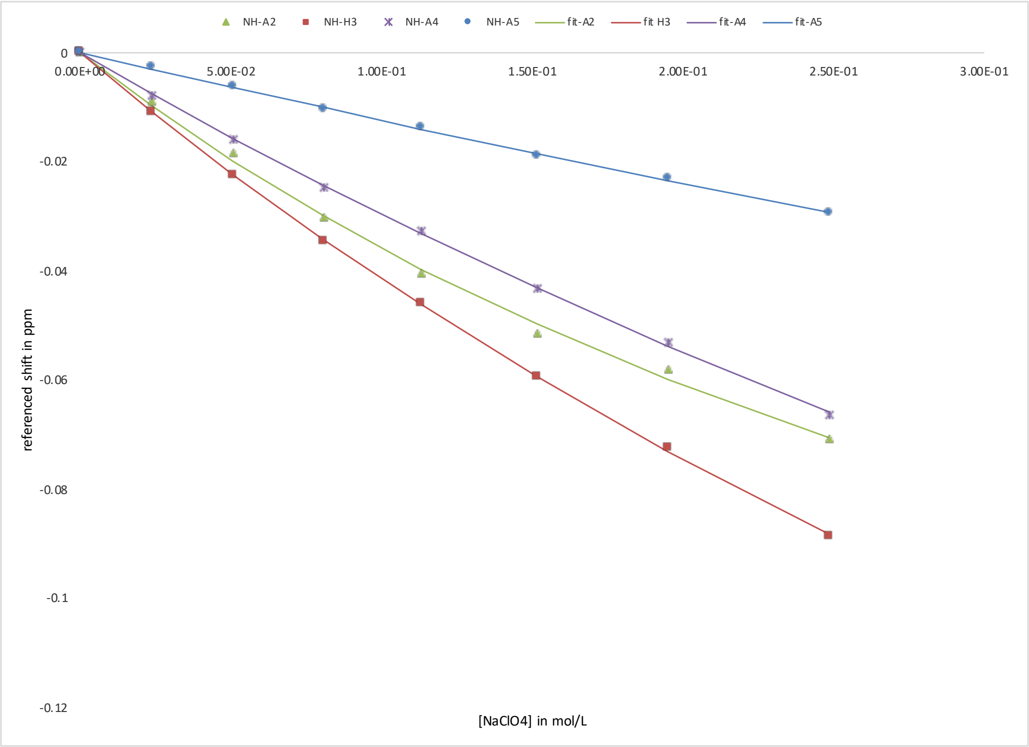

Figure S40: Fitted data from one of the titration **2** with $NaClO_4$ (0-250 mM salt). Data from Figure S33, and referenced to the 5A methyl signal. Solution was 1 mM **2** in 90% H_2O:10% D_2O, 10 mM acetate buffer, pH 5.2. Data was collected at 700 MHz.

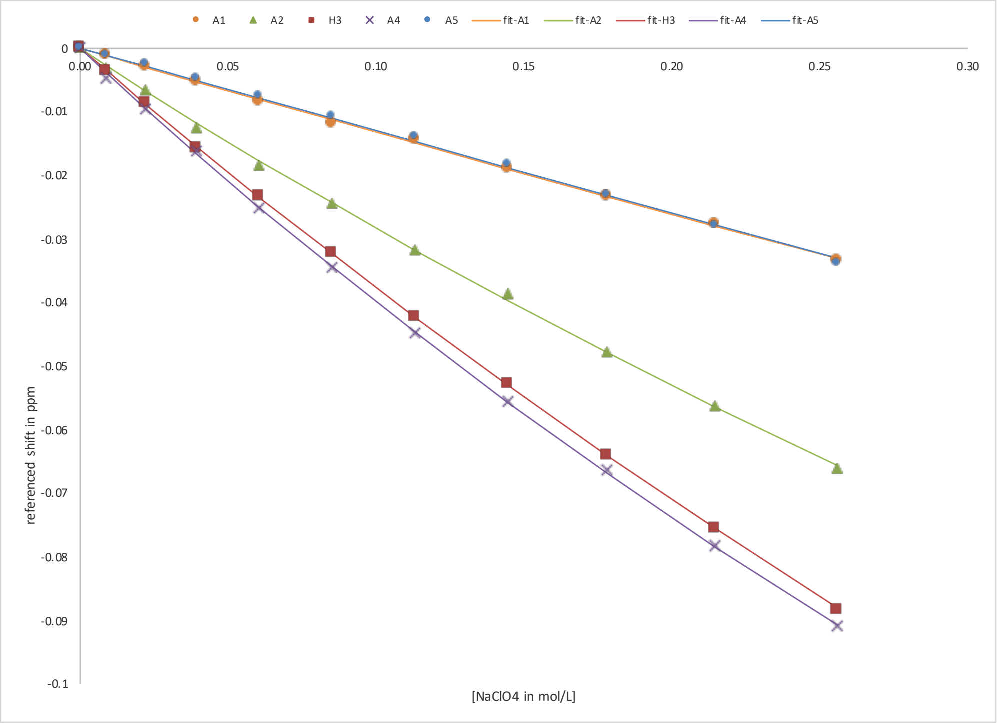

Figure S41: Fitted data from one of the titration **Ac-2** with $NaClO_4$ (0-250 mM salt). Data from Figure S34, and referenced to the 5A methyl signal. Solution was 1 mM Ac-**2** in 90% H_2O:10% D_2O, 10 mM acetate buffer, pH 5.2. Data was collected at 400 MHz.

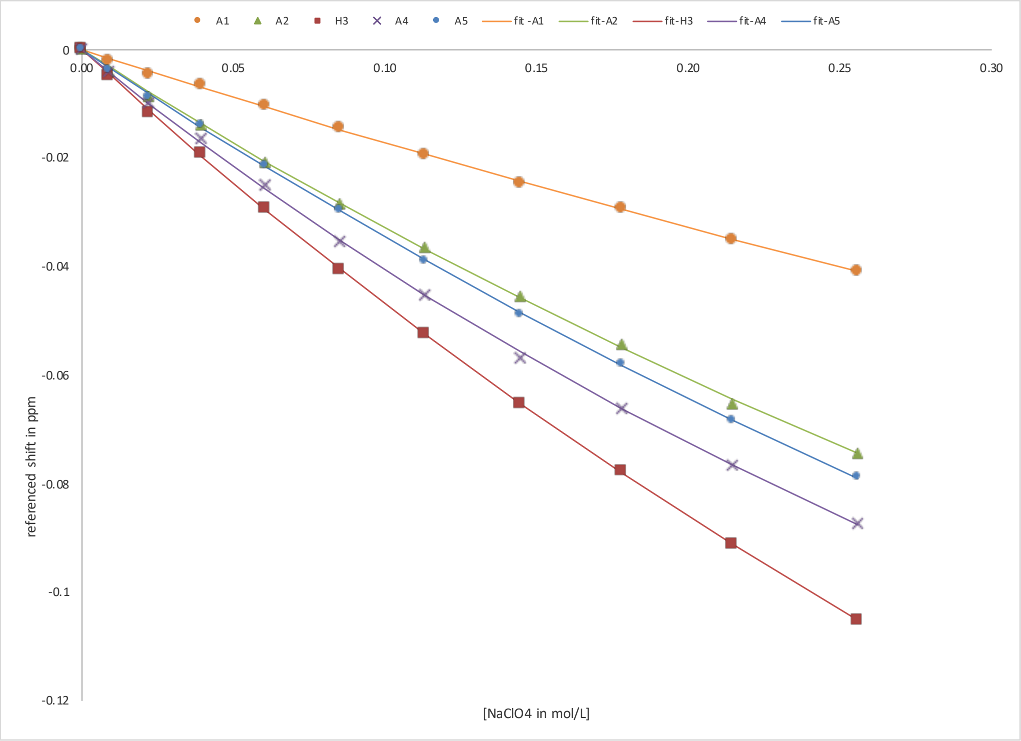

Figure S42: Fitted data from one of the titration **Ac-2-NH₂** with $NaClO_4$ (0-250 mM salt). Data from Figure S35, and referenced to the 5A methyl signal. Solution was 1 mM **Ac-2-NH₂** in 90% H_2O:10% D_2O, 10 mM acetate buffer, pH 5.2. Data was collected at 400 MHz.

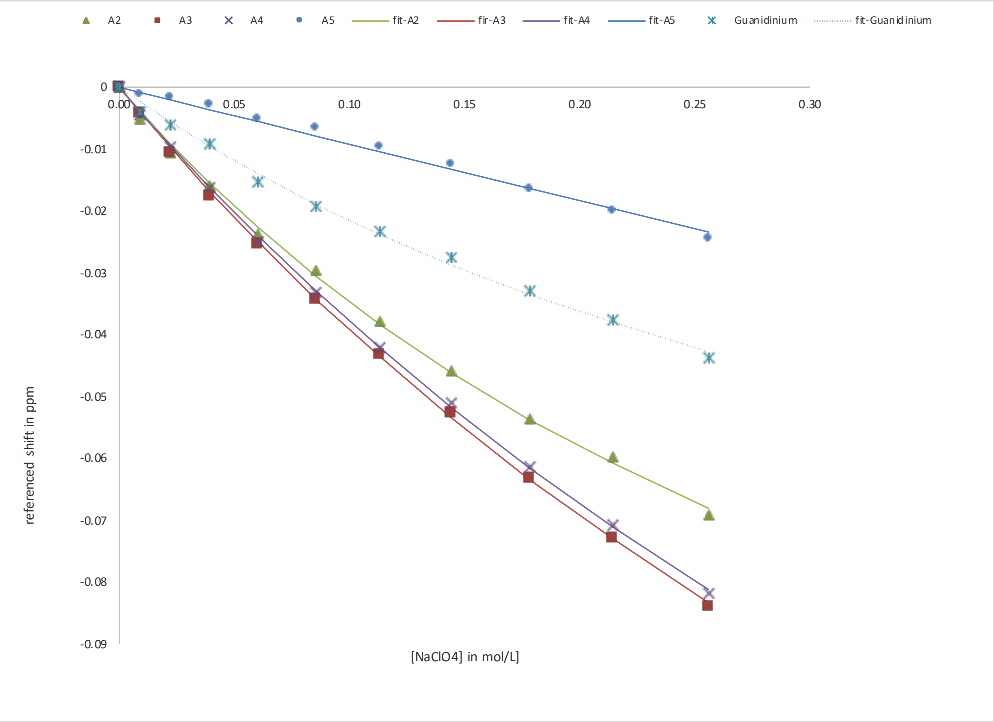

Figure S43: Fitted data from one of the titration **3** with $NaClO_4$ (0-250 mM salt). Data from Figure S36, and referenced to the 5A methyl signal. Solution was 1 mM **3** in 90% H_2O:10% D_2O, 10 mM acetate buffer, pH 5.2. Data was collected at 400 MHz.

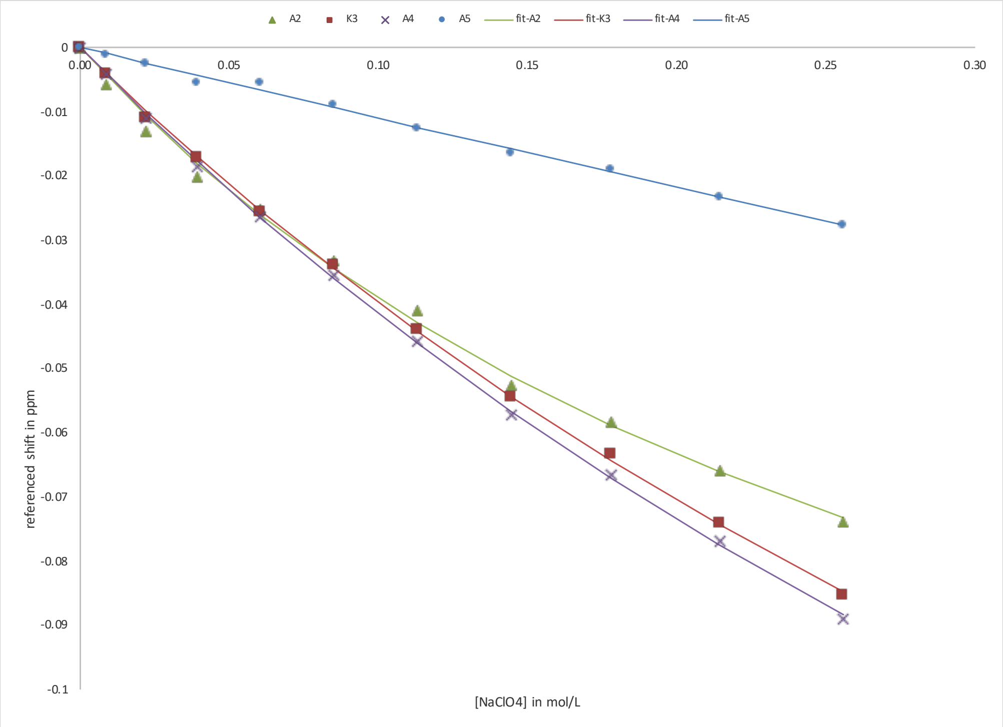

Figure S44: Fitted data from one of the titration **4** with $NaClO_4$ (0-250 mM salt). Data from Figure S37, and referenced to the 5A methyl signal. Solution was 1 mM **4** in 90% H_2O:10% D_2O, 10 mM acetate buffer, pH 5.2. Data was collected at 400 MHz.

7) Other anions binding to peptides 1 and 3

The ^{1}H NMR of peptide **3** showed the greatest signal anisotropy and was therefore selected for eleven-point titrations with NaCl and NaI to examine ion specific association. Because Cl^- and I^- induced small shifts in **1** accurate titration curves could not be obtained with this peptide. As a result, only single point titrations (250 mM) involving **1**, and NaCl and NaI were performed (see below). All titrations were performed three times.

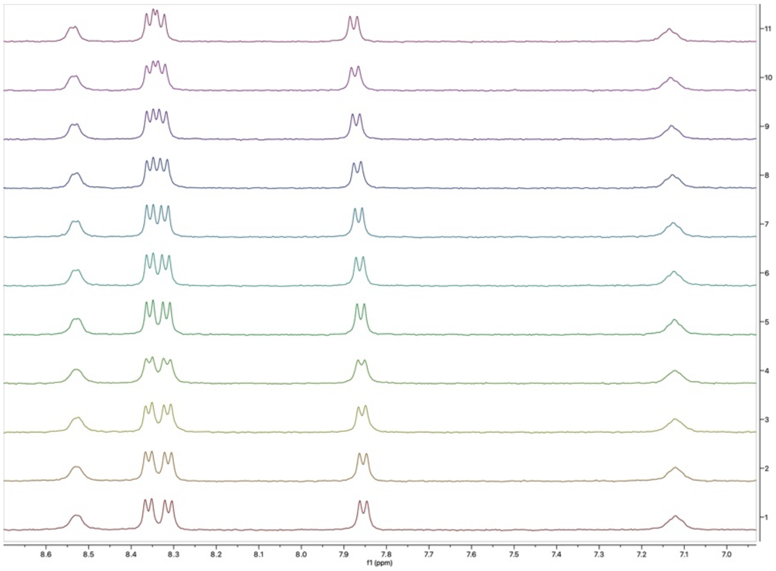

Figure S45: ^{1}H NMR (400 MHz) amide N-H region for the titration of 1 mM **3** with NaCl from 0 mM (NMR-1) to 250 mM (NMR-11). All solutions were 10 mM acetate buffer, pH 5.2.

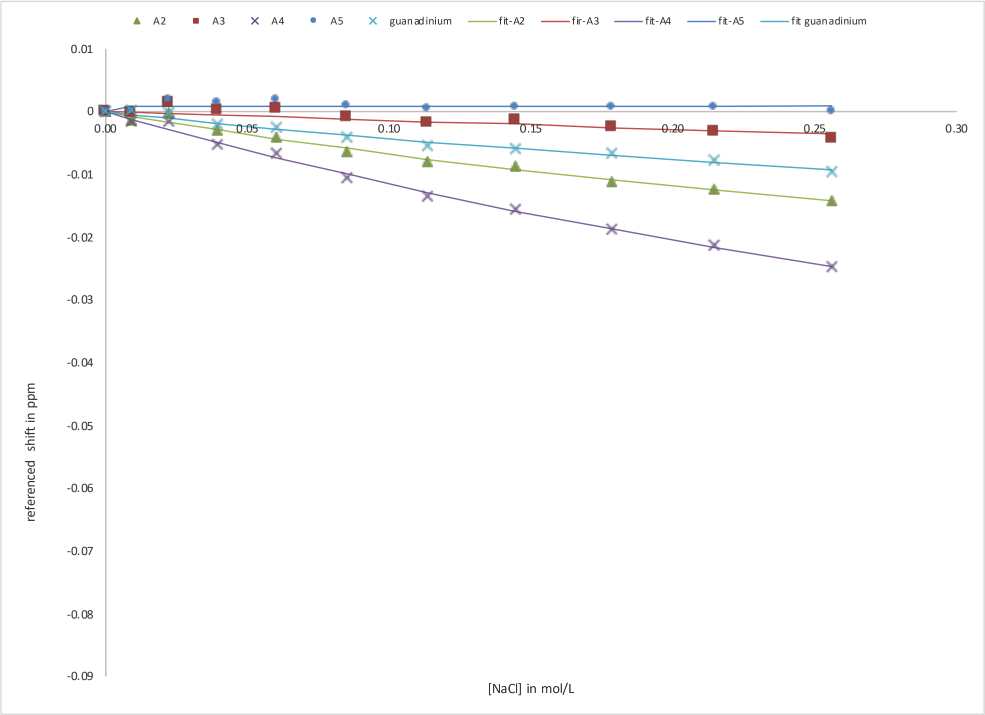

Figure S46: Fitted NMR data from one of the titrations of 1 mM **3** with NaCl (0 to 250 mM). Data from Figure S45. Proton shift referenced to methyl group of A5.

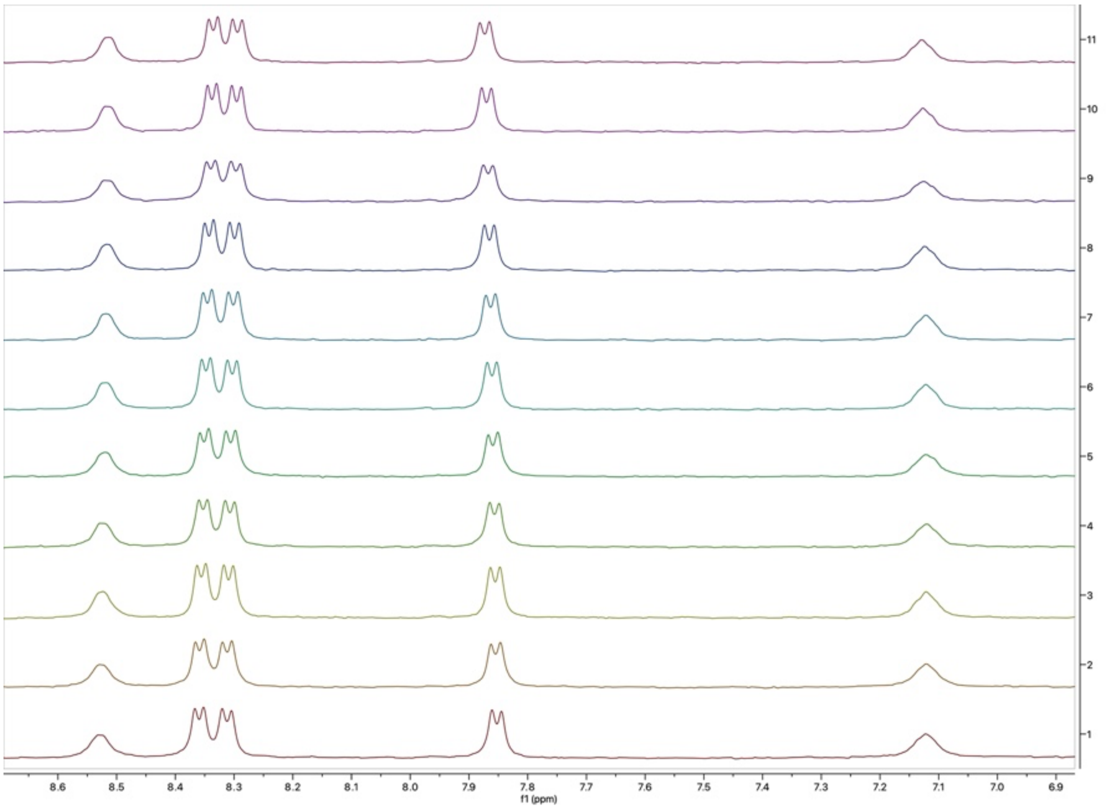

Figure S47: ^{1}H NMR (400 MHz) amide N-H region for the titration of 1 mM **3** with NaI from 0 mM (NMR-1) to 250 mM (NMR-11). All solutions were 10 mM acetate buffer, pH 5.2.

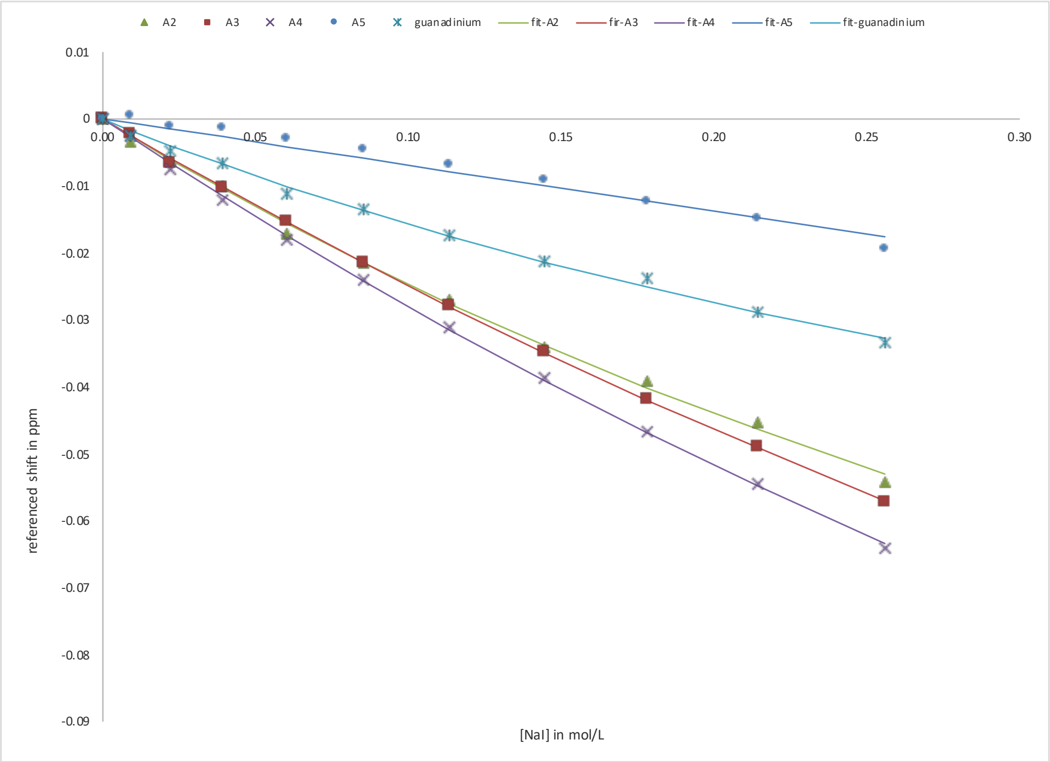

Figure S48: Fitted NMR data from one of the titrations of 1 mM **3** with NaI (0 to 250 mM). Data from Figure S47. Proton shift referenced to methyl group of A5.

8) K_a and Δδ Data Summary and Analyses

K_a Values

Table S1 shows the 1D NMR titration affinity determinations for peptides **1-4** in response to various anions. This data is also plotted in Figure S49-S51.

Peptide	Anion	Residue 1	Residue 2	Residue 3	Residue 4	Residue 5	Sidechain
**1**	ClO_4^-		1.16 (0.49)	0.41 (0.39)	0.15 (0.14)	0.02 (0.02)	
**2**	ClO_4^-		1.73 (0.48)	1.03 (0.20)	0.63 (0.23)	0.13 (0.18)	
Ac-**2**	ClO_4^-	0.34 (0.19)	0.77 (0.11)	0.62 (0.07)	1.00 (0.20)	0.12 (0.16)	
Ac-**2**-NH_2	ClO_4^-	0.69 (0.41)	0.99 (0.30)	1.16 (0.26)	1.42 (0.17)	0.98 (0.27)	
**3**	ClO_4^-		1.92 (0.64)	1.53 (0.47)	1.38 (0.13)	0.01 (0.01)	1.33 (0.78)
**3**	I^-		1.61 (0.23)	1.03 (0.19)	1.09 (0.14)	0.02 (0.01)	1.47 (0.24)
**3**	Cl^-		2.69 (1.05)	0.12 (0.03)	1.53 (0.46)	Could not fit	0.96 (0.16)
**4**	ClO_4^-		1.94 (1.22)	1.02 (0.64)	1.01 (0.63)	0.05 (0.08)	

Table S1: Average binding constant and standard deviation (in parenthesis) from 1:1 fitting of the titration data shown in Figures S32 to S48. Each titration was repeated at least in triplicate. The data in grey shaded cells was obtained from very small signal shifts; typically, less than 0.025 ppm at the end of the titration (250 mM salt). Percentage errors vary from 12% to 150%.

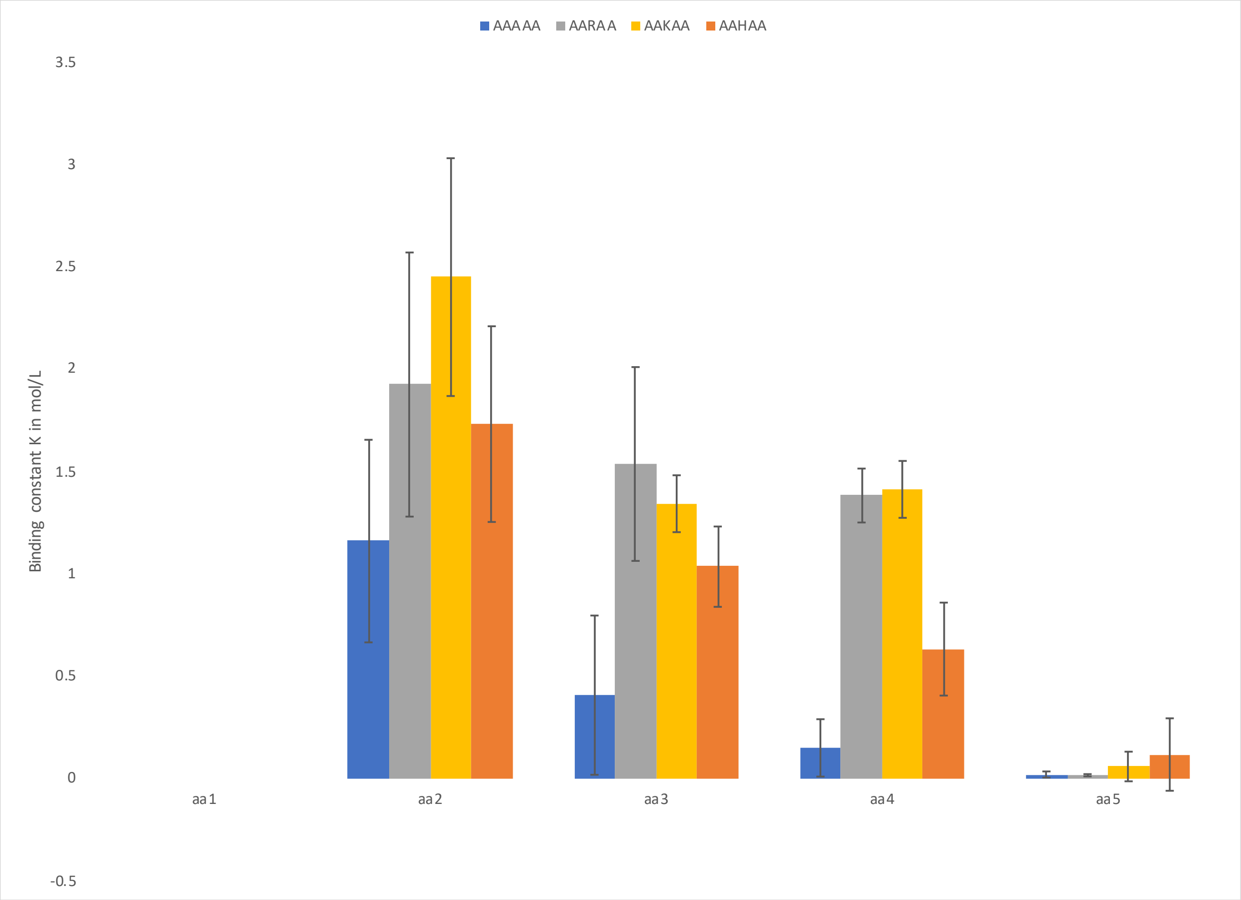

Figure S49: Average 1:1 binding constant for ClO_4^- as reported by each the N–H from each residue (see Figures S32 to S48). Error bar shows the standard deviation from at least three titrations.

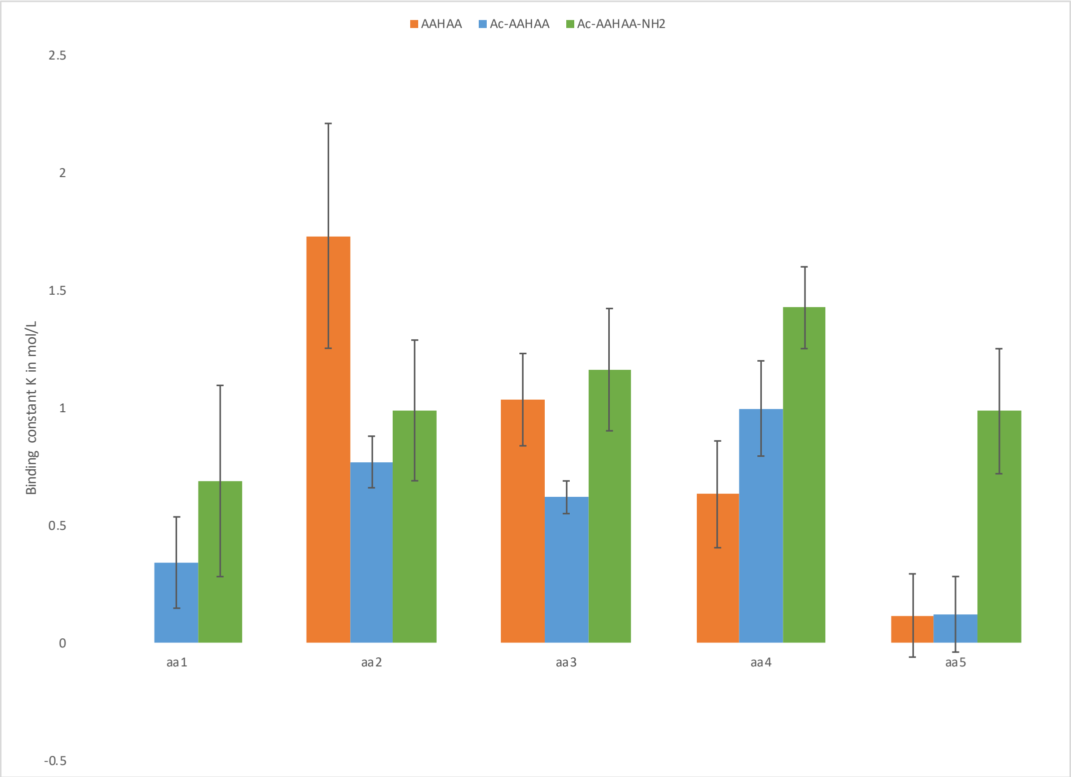

Figure S50: Average 1:1 binding constant for ClO_4^- as reported by each the N–H from each residue (see Figures S32 to S48). Error bar shows the standard deviation from at least three titrations.

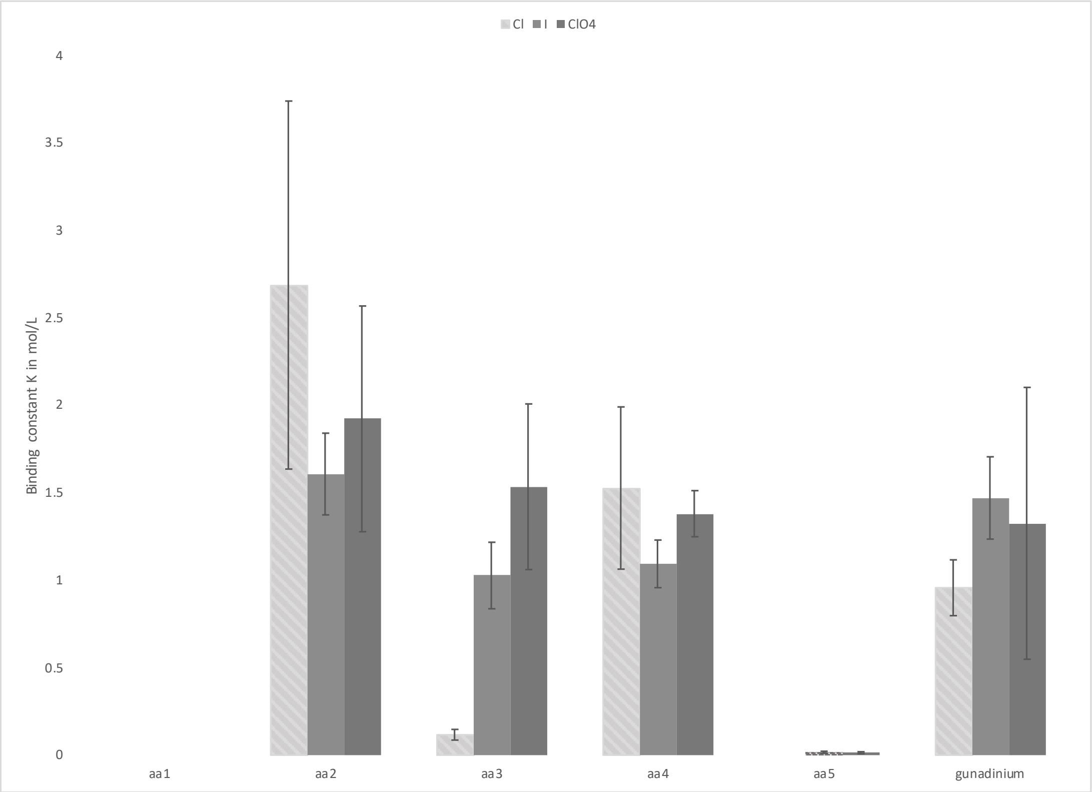

Figure S51: Average 1:1 binding constant for Cl^-, I^-, ClO_4^- as reported by each the N–H from each residue (see Figures S32 to S48). Error bar shows the standard deviation from at least three titrations involving **3**. Bars shown in stripes result from data with overall shift of < 0.025 ppm.

Signal shift (Δδ) Data

Table S2 shows the Δδ amide N–H signal shift for peptides **1-4** in response to various anions. Data from either single-point 'titrations' (0 and 250 mM salt), or (separate) formal titration experiments with incremental addition of salt to a final concentration of 250 mM, gave the same results. The data presented in Table S2 is also plotted in Figure S52-S55.

Peptide	Anion	Residue 1	Residue 2	Residue 3	Residue 4	Residue 5	Sidechain
**1**	ClO_4		-0.051	-0.049	-0.044	-0.018	
	I		-0.038	-0.035	-0.034	-0.014	
	Cl		-0.009	-0.007	-0.011	-0.001	
**2**	ClO_4		-0.078	-0.092	-0.069	-0.031	
**Ac-2**	ClO_4	-0.034 +/- 0.002	-0.067	-0.090	-0.092	-0.035	
Ac-**2**-NH_2	ClO_4	-0.040 +/- 0.001	-0.073	-0.103	-0.085	-0.077	
**3**	ClO_4		-0.071	-0.086	-0.084	-0.025	-0.043
	I		-0.053	-0.057	-0.063	-0.019	-0.033
	Cl		-0.015	-0.005	-0.025	0.000	-0.009
**4**	ClO_4		-0.069	-0.079	-0.082	-0.025	

Table S2: Referenced single point N-H proton shifts (ppm) at 250 mM salt ($_{ref}\Delta\delta_{(250mM)}$). The grey shading highlights the points where the overall shift is small <0.025 ppm. Values represent the average of at least three titrations. All standard deviations < 0.006 ppm.

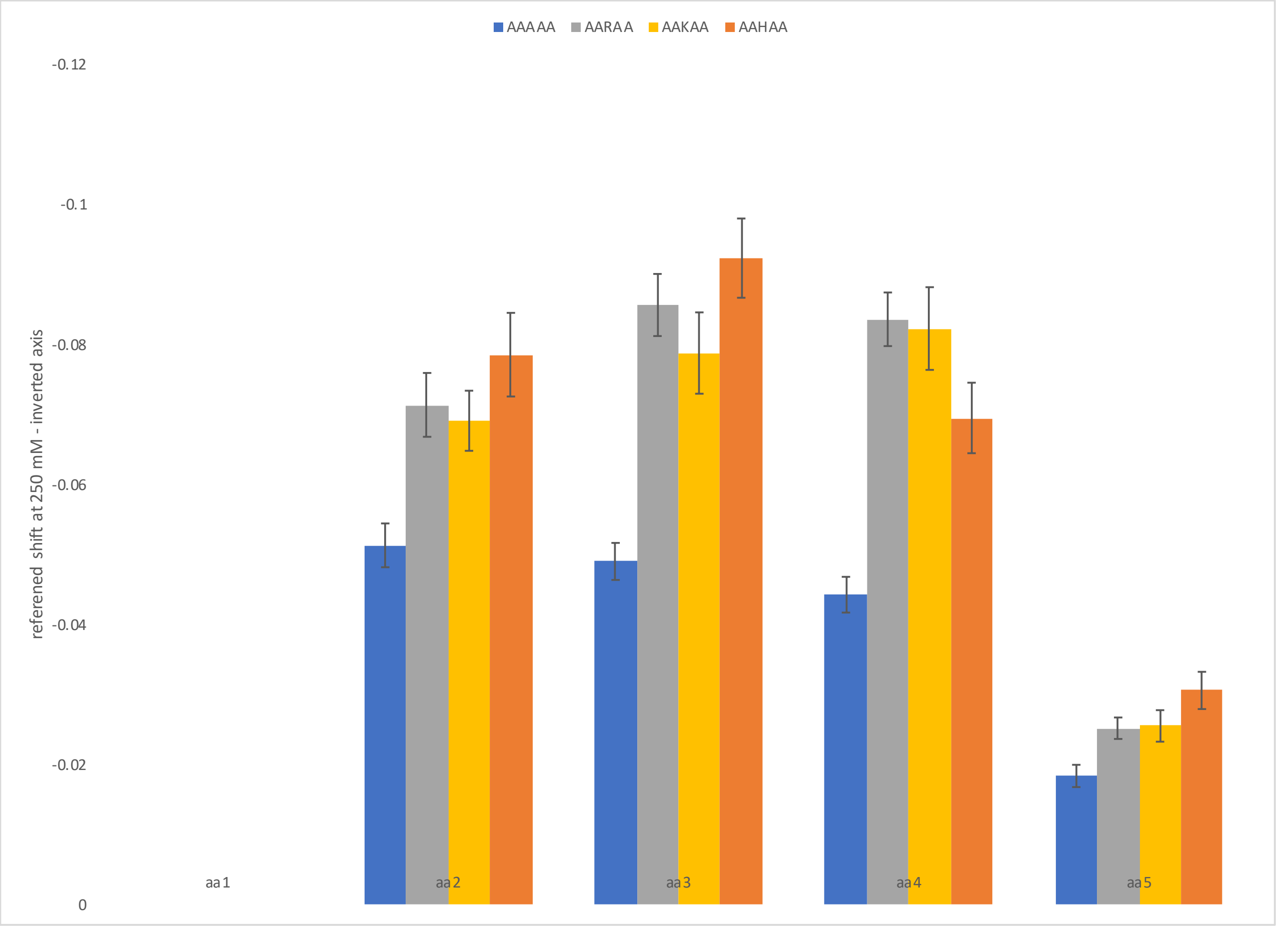

Figure S52: Referenced signal shifts at 250 mM $NaClO_4$ for each mainchain amide N-H group. Error bar shows the standard deviation from at least three titrations.

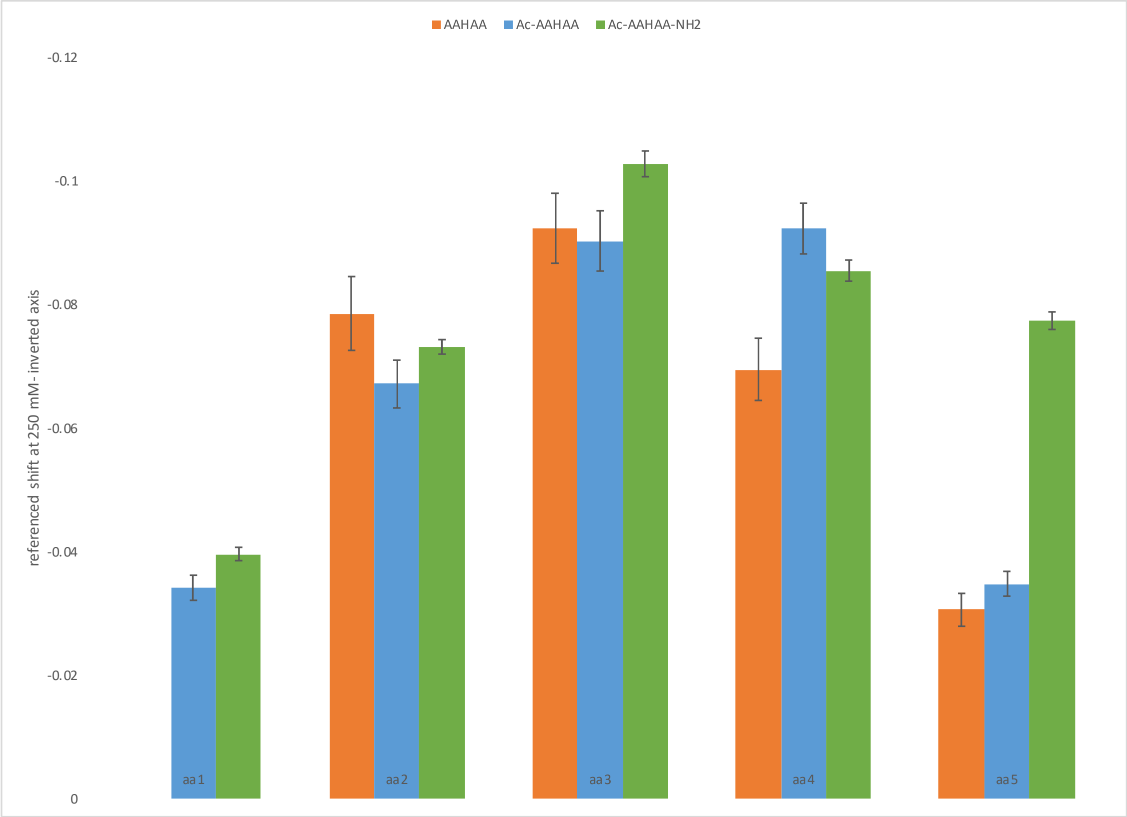

Figure S53: Referenced signal shifts at 250 mM $NaClO_4$ for each mainchain amide N-H group. Error bar shows the standard deviation from at least three titrations.

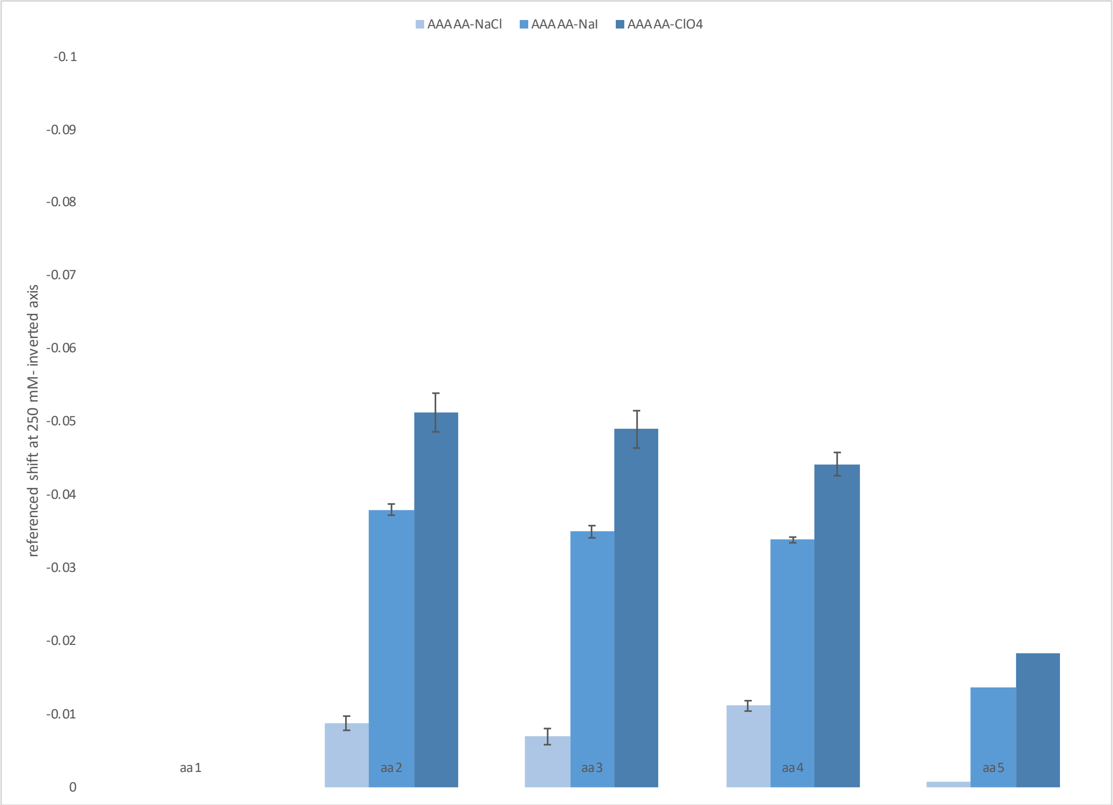

Figure S54: Referenced signal shifts at 250 mM NaCl, NaI, $NaClO_4$ for each mainchain amide N-H group of **1**. Error bar shows the standard deviation from at least three titrations.

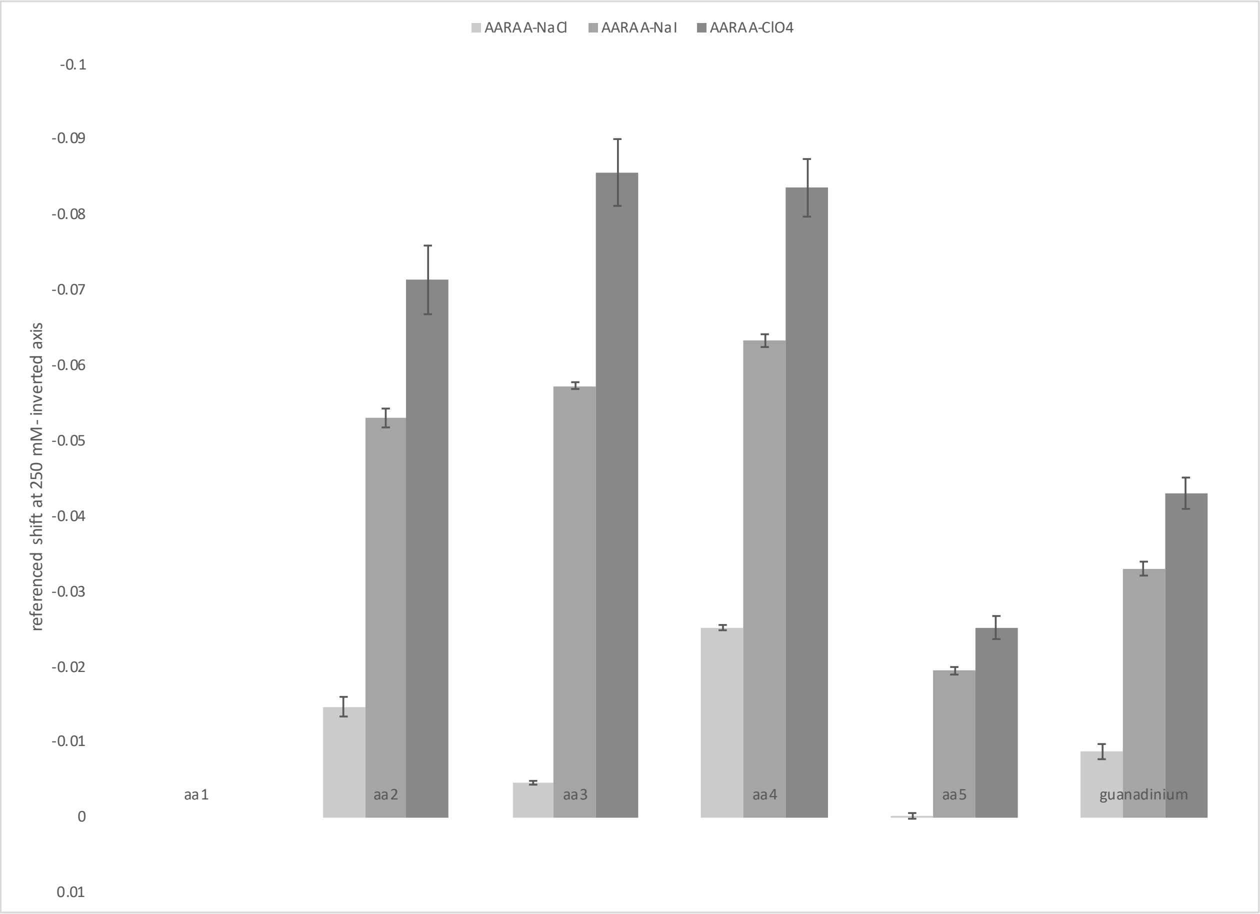

Figure S55: Referenced signal shifts at 250 mM NaCl, NaI, $NaClO_4$ for each mainchain amide N-H group of **3**. Error bar shows the standard deviation from at least three titrations.

9) Pentapeptide spatial distribution function (SDF) calculations and visualization

a) Spatial distribution functions

A series of molecular dynamics (MD) simulations were performed using the GROMACS 2016.3 package[3] on four pentapeptides of sequence AAXAA (X = Ala, Lys, Arg or His). For X = His, two conformations differing in the orientation of the imidazolium ring of His-3 relative to the mainchain: the dihedral, N_{amide}–$C_α$-$C_β$–$C_γ$ = +139° and –41° (Confo. **I** and **II** respectively). The atoms in histidine were labelled as per the force field parameters (Figure S56).

The simulations were performed in bulk water at 25 °C and 1 bar. The peptides were modelled using the Amber-ff03 all-atom force field[4, 5] the ions (chloride and perchlorate) were modelled using the generalized Amber force field (GAFF)[6] with their partial charges obtained from AM1-BCC calculations[7] while water was modelled using TIP4P2005.[8] The net charge on the peptides containing a basic residue was set to +1 to represent their protonation state at pH 5.2, while the pentapeptide containing only alanine was modeled in its neutral zwitterionic form.

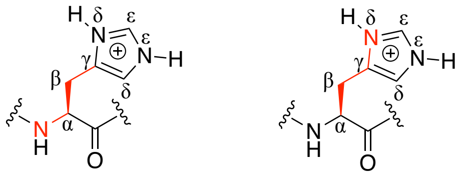

Figure S56: Dihedral angles adjusted for spatial distribution function and NMR signal shift prediction calculations.

Each simulation included one pentapeptide and thirty-three anions in a bath of three thousand water molecules. The simulations were run in the isothermal-isobaric ensemble, and the temperature and pressure maintained using the Nosé-Hoover thermostat,[9, 10] and the Parrinello-Rahman barostat[11] respectively. The equations of motion were integrated using a leapfrog algorithm with a time step of 2 fs. Electrostatic interactions were evaluated using particle mesh Ewald summation with a real space cutoff of 9 Å.[12] All simulations were performed for 200 ns in a cubic box with periodic boundary conditions. The radial distribution functions of the backbone N-H groups and the anions were calculated. The atom-atom potentials of anion (A) and nitrogen atom (N) are described as (Eq. 3):

$$\psi(r_A, r_N) = \frac{q_A q_N}{r_{AN}} + 4\varepsilon_{AN}\left[\left(\frac{\sigma_{AN}}{r_{AN}}\right)^{12} - \left(\frac{\sigma_{AN}}{r_{AN}}\right)^{6}\right] \qquad \text{Eq. 3}$$

where off-diagonal Lennard-Jones parameter σ_{AN} and ε_{AN} are given using the combination rules (Eq. 4 and 5):[13]

$$\sigma_{AN} = \frac{(\sigma_A + \sigma_N)}{2} \qquad \text{Eq. 4}$$

$$\varepsilon_{AN} = \sqrt{\varepsilon_A \varepsilon_N} \qquad \text{Eq. 5}$$

The trajectories from those simulations were then extracted and rendered as volumes – spatial distribution function using TRAVIS,[14, 15] and visualized using ChimeraX.[16] Figure S57 shows the calculated regions of anion association.

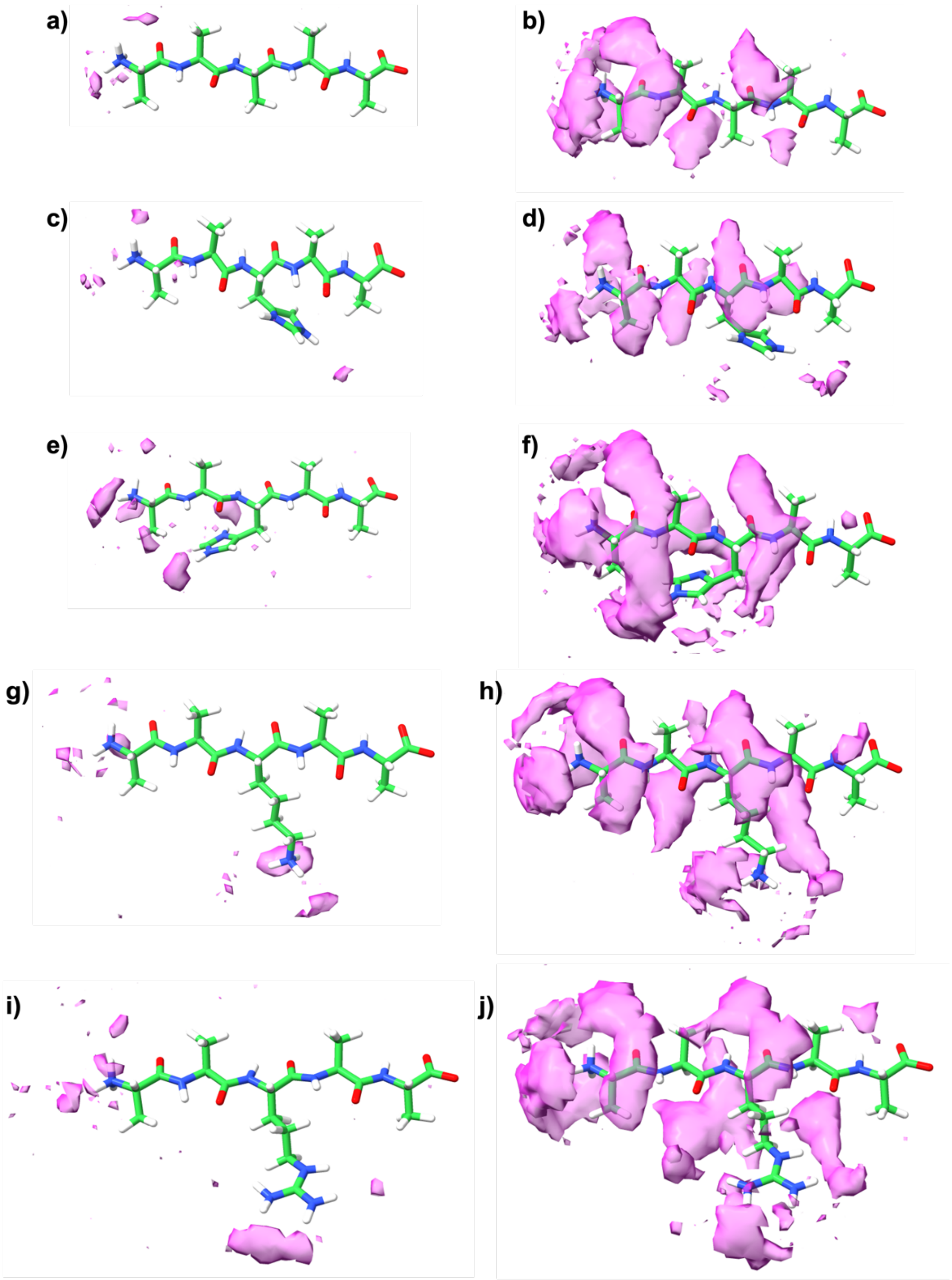

Figure S57: SDF visualization of anion association to peptides **1**-**4**. All anionic "clouds" (magenta) correspond to probability thresholds set to 12 × bulk density. The lefthand and righthand columns show Cl^- and ClO_4^- association respectively. Peptides **1** (a and b), **2** Confo. **I** (c and d), **2** Confo. **II** (e and f), **3** (g and h), and **4** (i and j).

To evaluate the influence of the positively charged *N*-terminus in anion binding, two protected peptide models were also prepared: Ac-**1** and Ac-**2** (Confo. **I**). The charge on the H3 residue of Ac-**2** was set to +1 to represent its protonation state at pH 5.2, bringing the overall charge of this peptide to 0, and the overall charge of Ac-**1** was set at -1. MD simulations of these two peptides in Cl^- and ClO_4^- were performed using the same parameters described above. Predicted anion probabilities 'clouds' are shown in Figure S58.

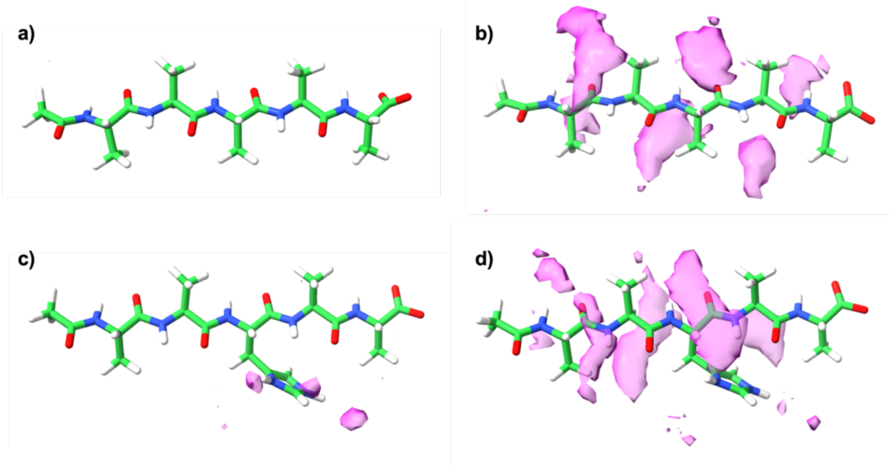

Figure S58: SDF visualization of anion association to peptides. All anionic "clouds" (magenta) are probability thresholds set to 12 × bulk density: a) **Ac-1** and Cl^-; b) **Ac-1** and ClO_4^-; c) **Ac-2** and Cl^-; d) **Ac-2** and ClO_4^-.

b) Peptide **2** NMR signal shift predictions

NMR tensors and magnetic susceptibilities of N-H signals of various conformations of peptide **2** were calculated with the Gauge-Independent Atomic Orbital (GIAO) method[17, 18] at the B3LYP/6-311+G(2d,p)[19, 20] level (Gaussian 16 package). A polarizable continuum water model was applied. Chemical shifts of a molecule of tetramethylsilane (TMS) were also calculated and used as a reference. For reference, the starting coordinates of **2** Confo. **II** (arbitrarily selected)) with a counterion Cl^- is given below in Table S3.

N	9.38220	-0.59512	-0.21069	C	-1.77138	3.70905	0.93188
C	8.15272	0.10238	0.19379	H	-2.19782	4.32121	1.75312
C	6.91309	-0.70849	-0.15599	N	-2.28228	3.57856	-0.40318
O	7.03170	-1.75712	-0.77550	C	-1.41996	2.70617	-1.09757
H	9.43818	-1.52446	0.26658	H	-1.54761	2.41357	-2.13148
H	8.17493	0.23722	1.29838	H	0.02062	2.75817	1.80451
C	8.09978	1.48731	-0.46997	H	1.92519	-1.57313	-0.55661
H	8.99844	2.07702	-0.19035	H	0.70979	0.30025	1.28010
H	8.07040	1.38598	-1.57616	N	-1.76663	-0.40237	0.26299
H	7.20724	2.05823	-0.14102	C	-2.96579	-1.19780	-0.00699
H	9.40637	-0.73525	-1.24762	C	-4.24980	-0.40958	0.23781
H	10.21847	-0.03548	0.07530	O	-4.19972	0.72282	0.69822
N	5.67643	-0.26407	0.21436	H	-1.86536	0.50798	0.72613
C	4.44245	-0.98794	-0.09845	H	-2.96788	-1.46934	-1.08586
C	3.19536	-0.17679	0.24493	C	-2.96240	-2.47036	0.85976
O	3.29959	0.89783	0.82027	H	-2.06515	-3.09183	0.67030
H	5.62705	0.63387	0.71609	H	-2.97614	-2.19567	1.93642
H	4.41540	-1.16840	-1.19580	H	-3.84517	-3.10678	0.64606
C	4.40470	-2.32934	0.65776	N	-5.45725	-0.96867	-0.06979
H	5.26869	-2.97165	0.39786	C	-6.73045	-0.27122	0.11521
H	4.42102	-2.14803	1.75392	C	-7.92342	-1.17788	-0.13117
H	3.49131	-2.90718	0.40874	O	-7.78517	-2.34227	-0.47280
N	1.96062	-0.65457	-0.09314	H	-5.45679	-1.92947	-0.44083
C	0.71531	0.06638	0.19249	H	-6.79929	0.07284	1.17051
C	-0.51244	-0.81298	-0.09027	C	-6.82596	0.93674	-0.83159
O	-0.36796	-1.89698	-0.63897	H	-6.01005	1.66486	-0.65428
C	0.67629	1.37139	-0.65426	H	-6.77161	0.59942	-1.88927
H	0.61710	1.09597	-1.73054	H	-7.78548	1.47502	-0.67739
H	1.60437	1.95846	-0.51494	O	-9.16852	-0.69407	0.04529
C	-0.44237	2.30514	-0.28781	H	-3.12219	4.03624	-0.78414
N	-0.59440	2.89153	0.98923	Cl	0.24787	-10.29924	1.94863

Table S3: Starting coordinates for **2** (Confo. **II**) with a counterion Cl^- for NMR tensors and magnetic susceptibility calculations.

To study the effects of different conformations of the histidine side chain upon the NMR signal positions of the N–H amide groups of peptide **2**, a series of input files were generated by rotating the histidine dihedral angle (N_{amide}–C_{α}-C_{β}–C_{γ}) every 45°, from Conf. **I** to Confo **II** (Figure S59). Each conformation was then subjected to identical NMR tensors and magnetic susceptibility calculations.

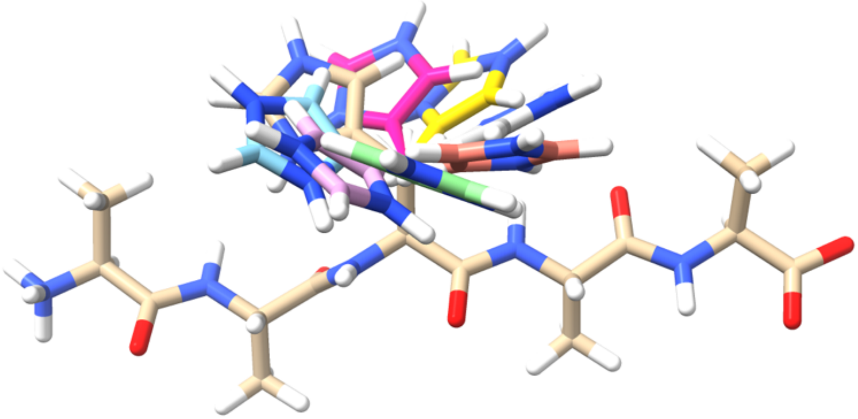

Figure S59: Peptide **2** (counterion Cl^- not shown) conformations by variation of the histidine dihedral N_{amide}–C_{α}-C_{β}–C_{γ} (See Figure S56). Shown are 45° rotations from Confo. **I** (red) to Confo. **II** (beige). Effects of histidine side chain orientation upon amide N–H NMR signal positions.

To further investigate the (de)shielding effects of the imidazole ring of His-3 on the mainchain amides N–H NMR signals of **2**, the dihedral angle C_{α}-C_{β}–C_{γ}–N_{δ} (Figure S56) was rotated every 45° in both Confo. **I** and Confo. **II** (Figure S60).

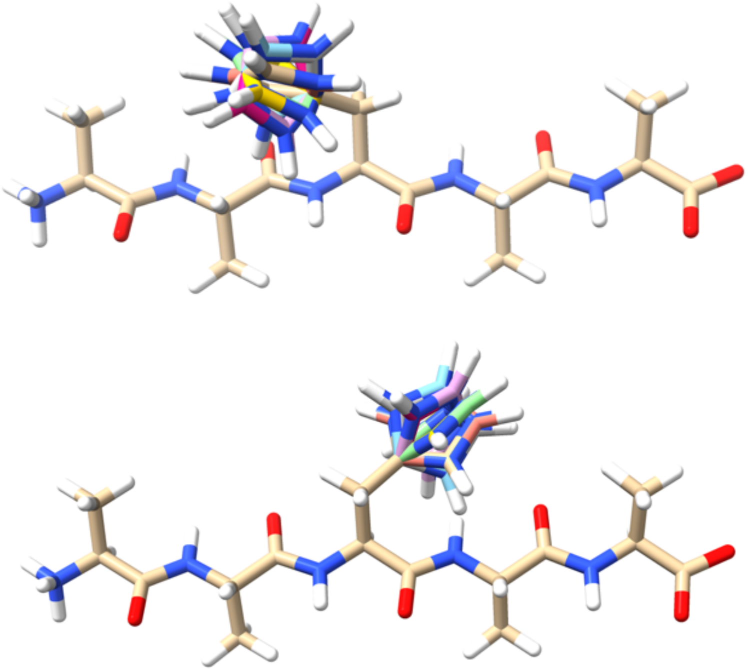

Figure S60: Peptide **2** (counterion Cl^- not shown) conformations (varying the dihedral angle C_{α}-C_{β}–C_{γ}–N_{δ}) to study the effects of imidazole ring orientation on the amide N–H NMR signals of Confo. **I** (*lower*) and Confo. **II** (*upper*).

Figure S61 shows the calculated mainchain amide proton signal positions for **2** as a function of changes in the N_{amide}–C_{α}-C_{β}–C_{γ} dihedral angle, converting Confo. **I** to Confo. **II (**N_{amide}–C_{α}-C_{β}–C_{γ} = +139° and –41° respectively). The calculations reveal that this movement of the imidazole ring has the largest effect on the amide N–H group of Ala-4.

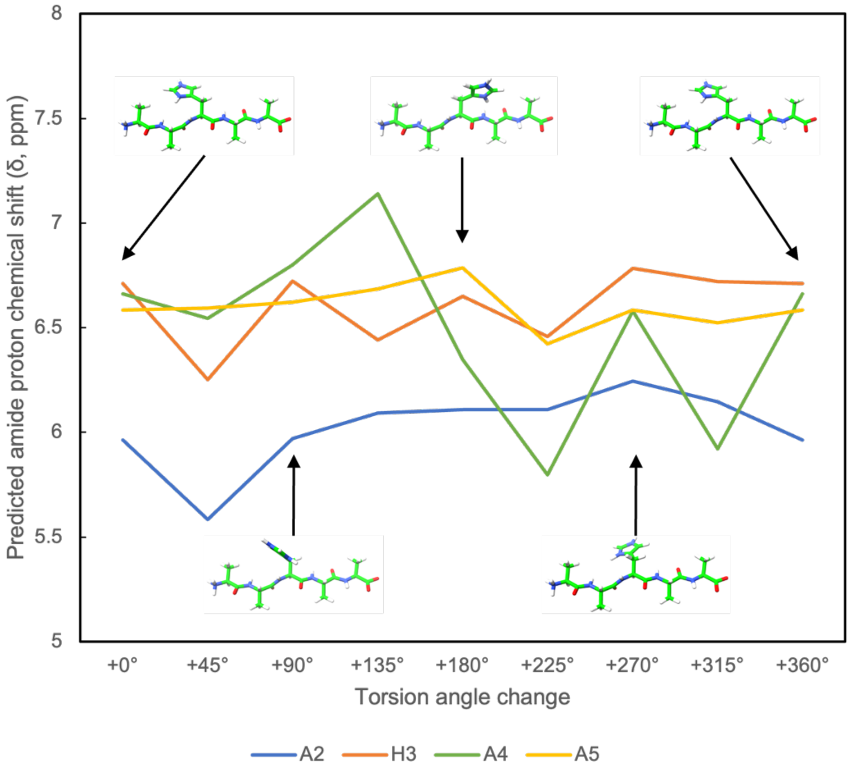

Figure S61: The calculated proton signal position (δ, ppm) of the amide N–H groups of **2**, as a function of changes in the dihedral angle N_{amide}–C_{α}-C_{β}–C_{γ} of His-3.

As Figure S62 demonstrates, changes to the C_α-C_β–C_γ–N_δ dihedral angle of Confo. **I** of peptide **2** results in the amide groups of Ala-2 and His-3 N–H undergoing small shifts, whilst Ala-4 and Ala-5 N–H undergo larger changes. The most upfield shifts of the N–H of Ala-4 is observed when the N–H bond is orthogonal to the imidazolium ring (+0°, +180°, and +225°), whilst its most downfield shifts are unrealistically high due to atom clashes.

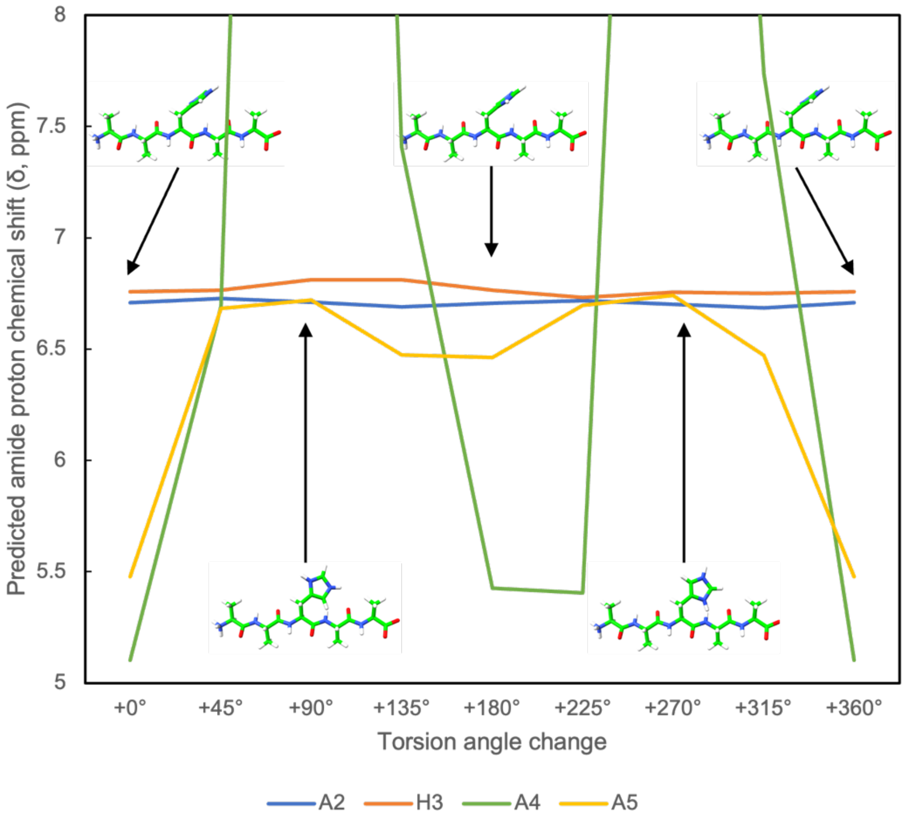

Figure S62: The calculated amide proton signal positions (δ, ppm) of Confo. **I** of **2** as a function of the dihedral angle C_α-C_β–C_γ–N_δ of His-3.

As Figure S63 demonstrates, changes to the C_α-C_β–C_γ–N_δ dihedral angle of Confo. **II** of peptide **2** results in the amide N–H signal of Ala-4 and Ala-5 N–H undergoing small shifts, whilst the corresponding shifts of Ala-2 and His-3 were more significant. The most downfield shifts of Ala-2 and His-3 N–H are observed when the imidazolium ring is perpendicular to those N–H bonds (C_α-C_β–C_γ–N_δ = +90° and +270°).

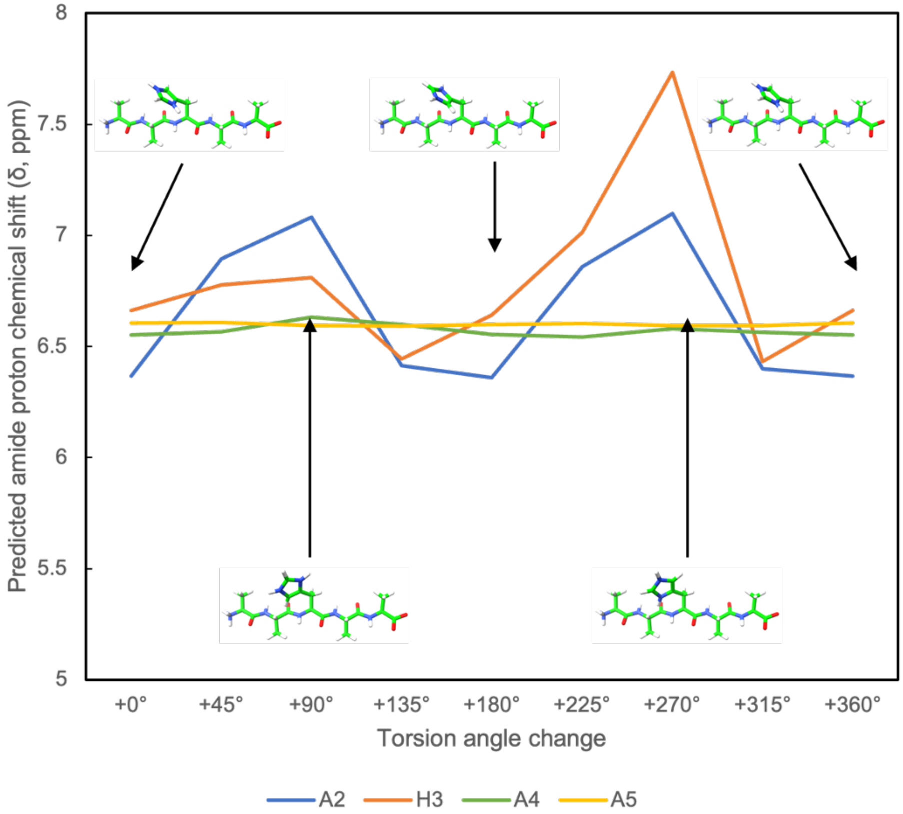

Figure S63: The calculated amide proton signal positions (δ, ppm) of Confo. **II** of **2** as a function of the dihedral angle C_{α}-C_{β}–C_{γ}–N_{δ} of His-3.

c) Electrostatic potential calculations

Electrostatic potential surfaces of two peptide series were generated using the same coordinates as used on the MD inputs. Specifically, the peptides AAXAA (X = Ala, His, Lys, Arg), and the protected peptides AcAAXAA (X = Ala, His) and Ac-AAHAA-NH_2 were investigated. Antechamber, employing the AM1-BCC technique, was used to compute partial atomic charges. The Coulombic electrostatic potential was determined based on these charges and atomic coordinates, utilizing Coulomb's law: $\varphi = \Sigma [qi / (\varepsilon di)]$. In this equation, φ represents the spatially varying potential, q denotes atomic partial charges, d corresponds to interatomic distances, and ε signifies the dielectric constant, representing medium screening effects. The resultant potential is expressed in kcal/(mol·e) units at a temperature of 298 K. The data was visualized by ChimeraX[16] and is shown in Figures S64 and S65.

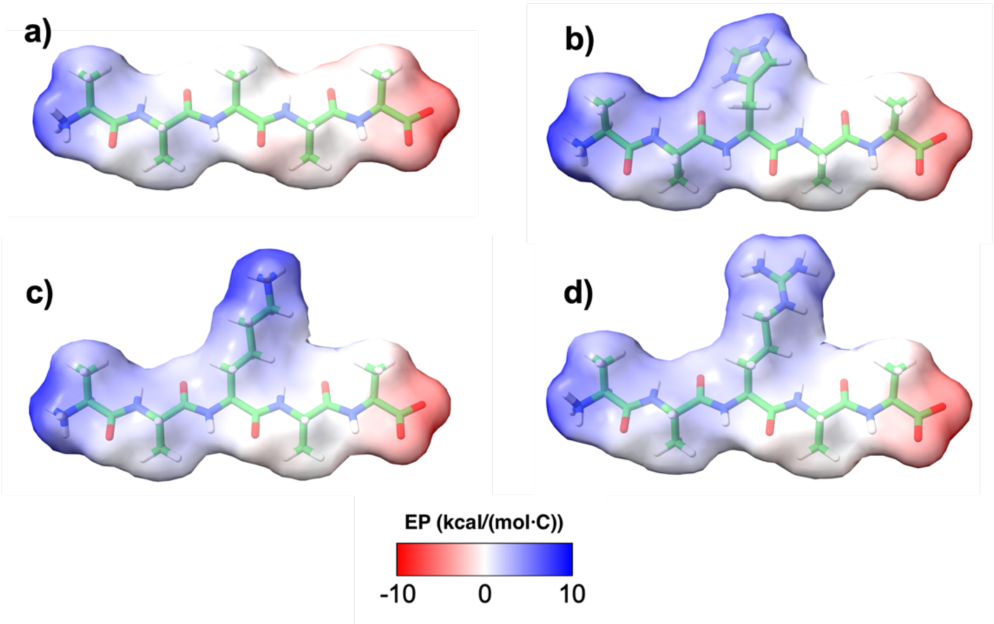

Figure S64: Electrostatic potential maps of peptides: a) **1** b) **2** c) **4**, and d) **3**.

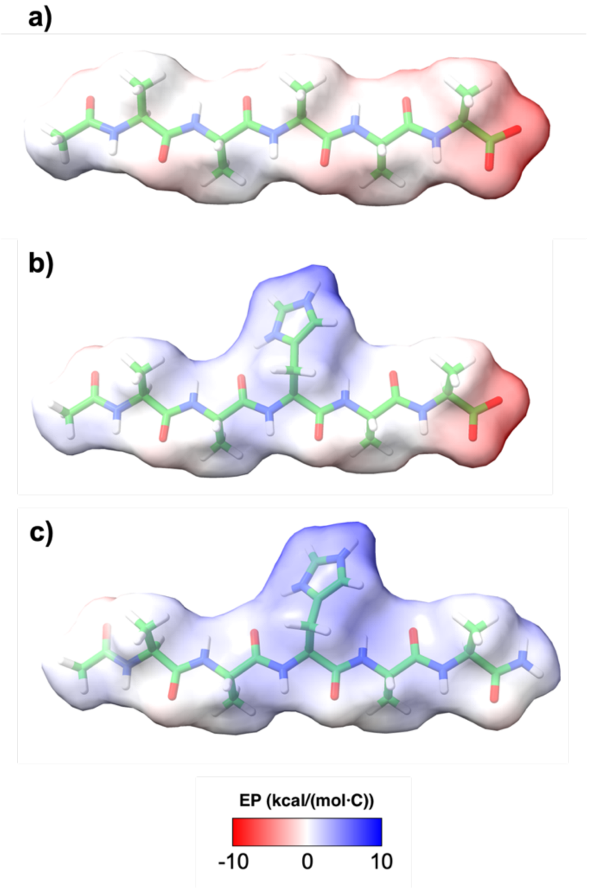

Figure S65: Electrostatic potential maps of peptides: a) **Ac-1** b) **Ac-2**, and c) **Ac-2-NH$_2$**.

10) References

(1) Gibb, C. L. D.; Gibb, B. C. The Thermodynamics of Molecular Recognition. In *Supramolecular Materials: From Molecules to Nanomaterials*, John Wiley and Sons, 2011; pp 45-66.
(2) Thordarson, P. Binding Constants and their Measurements. In *Supramolecular Chemistry: From Molecules to Nanomaterials*, Gale, P. A., Steed, J. W. Eds.; Vol. 2; John Wiley & Sons, Ltd., 2012; pp 1-20.
(3) Abraham, M. J.; Murtola, T.; Schulz, R.; Páll, S.; Smith, J. C.; Hess, B.; Lindahl, E. GROMACS: High performance molecular simulations through multi-level parallelism from laptops to supercomputers. *SoftwareX* **2015**, *1-2*, 19-25. DOI: https://doi.org/10.1016/j.softx.2015.06.001.
(4) Hornak, V.; Abel, R.; Okur, A.; Strockbine, B.; Roitberg, A.; Simmerling, C. Comparison of multiple Amber force fields and development of improved protein backbone parameters. *Proteins* **2006**, *65* (3), 712-725. DOI: 10.1002/prot.21123 PubMed.
(5) Martín-García, F.; Papaleo, E.; Gomez-Puertas, P.; Boomsma, W.; Lindorff-Larsen, K. Comparing Molecular Dynamics Force Fields in the Essential Subspace. *PLOS ONE* **2015**, *10* (3), e0121114. DOI: 10.1371/journal.pone.0121114.
(6) Wang, J.; Wolf, R. M.; Caldwell, J. W.; Kollman, P. A.; Case, D. A. Development and testing of a general amber force field. *J. Comput. Chem.* **2004**, *25* (9), 1157-1174. DOI: 10.1002/jcc.20035.
(7) Jakalian, A.; Jack, D. B.; Bayly, C. I. Fast, efficient generation of high-quality atomic charges. AM1-BCC model: II. Parameterization and validation. *J. Comp. Chem.* **2002**, *23* (16), 1623-1641, DOI: https://doi.org/10.1002/jcc.10128.
(8) Abascal, J. L. F.; Vega, C. A general purpose model for the condensed phases of water: TIP4P/2005. *J. Chem. Phys.* **2005**, *123* (23), 234505. DOI: 10.1063/1.2121687.
(9) Nosé, S. A unified formulation of the constant temperature molecular dynamics methods. *J. Chem. Phys.* **1984**, *81* (1), 511-519. DOI: 10.1063/1.447334 (acccessed 2023/01/02).
(10) Hoover, W. G. Canonical dynamics: Equilibrium phase-space distributions. *Physical Review A* **1985**, *31* (3), 1695-1697. DOI: 10.1103/PhysRevA.31.1695.
(11) Parrinello, M.; Rahman, A. Polymorphic transitions in single crystals: A new molecular dynamics method. *J. Appl. Phys.* **1981**, *52* (12), 7182-7190. DOI: 10.1063/1.328693 (acccessed 2022/06/16).
(12) Darden, T.; York, D.; Pedersen, L. Particle mesh Ewald: An N·log(N) method for Ewald sums in large systems. *J. Chem.Phys.* **1993**, *98* (12), 10089-10092. DOI: 10.1063/1.464397 (acccessed 2023/01/02).
(13) Hansen, J.-P.; McDonald, I. R. *Theory of simple liquids: with applications to soft matter*; Academic press, 2013.
(14) Brehm, M.; Kirchner, B. TRAVIS - A Free Analyzer and Visualizer for Monte Carlo and Molecular Dynamics Trajectories. *Journal of Chemical Information and Modeling* **2011**, *51* (8), 2007-2023. DOI: 10.1021/ci200217w.
(15) Brehm, M.; Thomas, M.; Gehrke, S.; Kirchner, B. TRAVIS—A free analyzer for trajectories from molecular simulation. *J. Chem. Phys.* **2020**, *152* (16), 164105. DOI: 10.1063/5.0005078.
(16) Pettersen, E. F.; Goddard, T. D.; Huang, C. C.; Meng, E. C.; Couch, G. S.; Croll, T. I.; Morris, J. H.; Ferrin, T. E. UCSF ChimeraX: Structure visualization for researchers, educators, and developers. *Protein Sci* **2021**, *30* (1), 70-82. DOI: 10.1002/pro.3943.

(17) Wolinski, K.; Hinton, J. F.; Pulay, P. Efficient implementation of the gauge-independent atomic orbital method for NMR chemical shift calculations. *J. Am. Chem. Soc* **1990**, *112* (23), 8251-8260. DOI: 10.1021/ja00179a005.
(18) Cheeseman, J. R.; Trucks, G. W.; Keith, T. A.; Frisch, M. J. A comparison of models for calculating nuclear magnetic resonance shielding tensors. *J. Chem. Phys.* **1996**, *104* (14), 5497-5509. DOI: 10.1063/1.471789.
(19) Safi, Z. S.; Wazzan, N. DFT calculations of ^{1}H- and ^{13}C-NMR chemical shifts of 3-methyl-1-phenyl-4-(phenyldiazenyl)-1H-pyrazol-5-amine in solution. *Sci. Rep.* **2022**, *12* (1), 17798. DOI: 10.1038/s41598-022-22900-y.
(20) Lodewyk, M. W.; Siebert, M. R.; Tantillo, D. J. Computational Prediction of ^{1}H and ^{13}C Chemical Shifts: A Useful Tool for Natural Product, Mechanistic, and Synthetic Organic Chemistry. *Chem. Rev.* **2012**, *112* (3), 1839-1862. DOI: 10.1021/cr200106v.
